# Viral anti‐inflammatory serpin reduces immuno‐coagulopathic pathology in SARS‐CoV‐2 mouse models of infection

**DOI:** 10.15252/emmm.202317376

**Published:** 2023-08-03

**Authors:** Liqiang Zhang, Yize (Henry) Li, Karen Kibler, Simona Kraberger, Arvind Varsani, Julie Turk, Nora Elmadbouly, Emily Aliskevich, Laurel Spaccarelli, Bereket Estifanos, Junior Enow, Isabela Rivabem Zanetti, Nicholas Saldevar, Efrem Lim, Jessika Schlievert, Kyle Browder, Anjali Wilson, Fernando Arcos Juan, Aubrey Pinteric, Aman Garg, Henna Monder, Rohan Saju, Savanah Gisriel, Bertram Jacobs, Timothy L Karr, Esther Borges Florsheim, Vivek Kumar, John Wallen, Masmudur Rahman, Grant McFadden, Brenda G Hogue, Alexandra R Lucas

**Affiliations:** ^1^ Center for Personalized Diagnostics, Biodesign Institute Arizona State University Tempe AZ USA; ^2^ Center of Immunotherapy, Vaccines and Virotherapy, Biodesign Institute Arizona State University Tempe AZ USA; ^3^ School of Life Sciences Arizona State University Tempe AZ USA; ^4^ Center of Fundamental and Applied Microbiomics Biodesign Institute, Arizona State University Tempe AZ USA; ^5^ Center for Evolution and Medicine, School of Life Sciences Arizona State University Tempe AZ USA; ^6^ Departments of Pathology & Lab Medicine Yale‐New Haven Hospital New Haven CT USA; ^7^ Neurodegenerative Disease Research Center & Proteomics Center, Biodesign Institute Arizona State University Tempe AZ USA; ^8^ New Jersey Institute of Technology Newark NJ USA; ^9^ Colt Advisors LLC Las Vegas NV USA; ^10^ Center for Applied Structural Discovery, Biodesign Institute Arizona State University Tempe AZ USA

**Keywords:** complement, inflammation, SARS‐CoV‐2, serpin, uPAR, Haematology, Microbiology, Virology & Host Pathogen Interaction

## Abstract

SARS‐CoV‐2 acute respiratory distress syndrome (ARDS) induces uncontrolled lung inflammation and coagulopathy with high mortality. Anti‐viral drugs and monoclonal antibodies reduce early COVID‐19 severity, but treatments for late‐stage immuno‐thrombotic syndromes and long COVID are limited. Serine protease inhibitors (SERPINS) regulate activated proteases. The myxoma virus‐derived Serp‐1 protein is a secreted immunomodulatory serpin that targets activated thrombotic, thrombolytic, and complement proteases as a self‐defense strategy to combat clearance. Serp‐1 is effective in multiple animal models of inflammatory lung disease and vasculitis. Here, we describe systemic treatment with purified PEGylated Serp‐1 as a therapy for immuno‐coagulopathic complications during ARDS. Treatment with PEGSerp‐1 in two mouse‐adapted SARS‐CoV‐2 models in C57Bl/6 and BALB/c mice reduced lung and heart inflammation, with improved outcomes. PEGSerp‐1 significantly reduced M1 macrophages in the lung and heart by modifying urokinase‐type plasminogen activator receptor (uPAR), thrombotic proteases, and complement membrane attack complex (MAC). Sequential changes in gene expression for uPAR and serpins (complement and plasminogen inhibitors) were observed. PEGSerp‐1 is a highly effective immune‐modulator with therapeutic potential for severe viral ARDS, immuno‐coagulopathic responses, and Long COVID.

## Graphical abstract

Here we examined a virus‐derived immune modulating serpin, PEGSerp‐1, as a treatment for severe viral infection. Later stage inflammatory and coagulation (thrombotic and thrombolytic) pathway activity cause prolonged disease. This is a new class of virus‐derived immune‐modulating protein biologic.

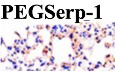

**The paper explained**

**Problem**

With many severe virus infections, such as COVID‐19, there can be an excess immune response in the body resulting in damage to the heart, lungs, and other organs. Whole‐body clotting is seen after COVID‐19 infections and that also causes damage. There are few effective treatments for this late viral inflammatory and clotting damage.

**Results**

Here, we tested a new drug, PEGSerp‐1, a protein member of the serpin family that is derived from a virus and that reduces immune and clotting damage. PEGSerp‐1 improved responses to the SARS‐CoV‐2 infection in mouse models by reducing damaging immune and coagulation enzyme activation in the lungs and heart, markers for severe long‐term virus side effects.

**Impact**

A new serpin drug targets sites of tissue damage, providing a new class of treatments for late severe and long‐term viral damage, such as seen in long COVID.

## Introduction

Excessive clotting, bleeding, and inflammation increase lung damage, hypoxia, and mortality in the severe acute respiratory distress syndrome (ARDS) associated with the later stages of Coronavirus‐2 (SARS‐CoV‐2) lung infections. Mortality rates of up to 20–45% have been reported for hypoxic patients admitted to intensive care units (ICU; Arnold *et al*, 2021; de Bruina *et al*, 2021; Keskinidou *et al*, 2021; The PHOSP‐COVID Collaborative Group, 2022). With severe viral infections, ARDS induces aggressive and uncontrolled inflammation that fails to shut off, thereby causing further damage with lung consolidation, hypoxia, coagulopathy, and increased mortality (Bradley & Bryan, 2019; Arnold *et al*, 2021; de Bruina *et al*, 2021; Jordan, 2021; Keskinidou *et al*, 2021; Kurtovic & Beeson, 2021; Perico *et al*, 2021; The PHOSP‐COVID Collaborative Group, 2022). In healthy physiologic states, there is a balance between reciprocal activation of the thrombotic and thrombolytic cascades and immune responses, providing the homeostasis that regulates uncontrolled coagulation and inflammation (Esmon *et al*, 2011; Goeijenbier *et al*, 2012; Keragala & Medcalf, 2021; Perico *et al*, 2021). ARDS with severe infections can unbalance this normal homeostasis. Disseminated intravascular coagulation in bacterial sepsis causes excessive thrombosis, sequestering clot‐forming proteases with subsequent bleeding induced by the lack of thrombotic proteases and a relative excess of clot‐dissolving, thrombolytic pathways. As with SARS‐CoV‐2, other respiratory viruses like SARS‐CoV‐1, Middle‐East respiratory syndrome (MERS) (Bradley & Bryan, 2019) and influenza (Nicholls *et al*, 2003; Kobasa *et al*, 2007; Maines *et al*, 2009; Calore *et al*, 2011; Esmon *et al*, 2011; Tate *et al*, 2011; Yu *et al*, 2011; Goeijenbier *et al*, 2012; Nakajima *et al*, 2012; von Ranke *et al*, 2013; Teijaro, 2015) can induce a damaging imbalance in immune and thrombotic/thrombolytic coagulation responses.

Current anti‐viral therapies can be highly effective as post‐exposure treatments for SARS‐CoV‐2 infections if administered in the early stages of viremia, reducing the risk of ICU admission, but the later‐stage immuno‐thrombotic complications of SARS‐CoV‐2 infections remain difficult to treat with 40–50% mortality in severe cases (Arnold *et al*, 2021; de Bruina *et al*, 2021; Keskinidou *et al*, 2021; The PHOSP‐COVID Collaborative Group, 2022). Corticosteroids are beneficial in patients with hypoxia and more advanced lung disease, but are only partially effective and are not recommended in early stages of COVID‐19 (Angus *et al*, 2020; The RECOVERY Collaborative Group, 2020; Arnold *et al*, 2021; de Bruina *et al*, 2021; Keskinidou *et al*, 2021; The PHOSP‐COVID Collaborative Group, 2022). Anticoagulants such as heparin have had variable benefits (Maldonado *et al*, 2020; Mir *et al*, 2021; Novelli *et al*, 2021; Wang *et al*, 2022). Targeted monoclonal antibodies against individual pro‐inflammatory cytokines also have limited benefits. Long‐term sequelae, termed long COVID, may be associated with the failure to turn off immune‐ and coagulation‐related complications in multiple organs, not just the lungs. There is at present no prevention or treatment for Long COVID (Acanfora *et al*, 2021; Aiyegbusi *et al*, 2021; Yong, 2021).

Serine protease cascades govern coagulation and innate immune responses and increased activation is detectable in severe SARS‐CoV‐2 lung infections, serving as markers for higher risk of ARDS, thrombosis, long‐term sequelae, and increased mortality (Bradley & Bryan, 2019; Yu *et al*, 2019; Yu & Liu, 2019; D'Alonzo *et al*, 2020; Acanfora *et al*, 2021; Arnold *et al*, 2021; de Bruina *et al*, 2021; Keskinidou *et al*, 2021; The PHOSP‐COVID Collaborative Group, 2022). Soluble urokinase‐type plasminogen activator receptor (suPAR) and activated complement are clinical markers for risk of ARDS with ICU admission in SARS‐CoV‐2 infections. The uPA/uPAR complex is positioned at the leading edge of migratory leukocytes that mediate the innate cellular immune responses and is a central mediator in inflammation, immunity, and coagulation. uPA/uPAR activates plasmin and matrix metalloproteinases, increasing inflammatory macrophage invasion, as well as growth factor activation. Soluble tPA, the principal thrombolytic protease, is also reported as increased in severe SARS‐CoV‐2 infections (Yu *et al*, 2019; Yu & Liu, 2019; D'Alonzo *et al*, 2020; Acanfora *et al*, 2021; Yong, 2021; Zuo *et al*, 2021). The complement cascade is also comprised of multiple sequentially activated serine proteases that form the complement C5b‐9 membrane attack complex (MAC), an effector for innate and acquired immune responses (Cugno *et al*, 2021). The uPA, tPA, thrombin, and complement cascades are all regulated by mammalian serpins, representing 2% or more of circulating plasma proteins (Bouton *et al*, 2023). Serpins have been examined both as biomarkers for disease as well as potential therapeutics for genetic serpinopathy disorders and for infection‐related coagulopathies.

Many large DNA viruses have evolved highly potent immune‐modulating proteins over eons of selective evolutionary pressures, as self‐protective strategies against immune cells of various classes. Virus‐derived immune‐modulating proteins provide a natural reservoir for biologics designed to block pivotal regulatory pathways in the immune response (Lucas & McFadden, 2004; Yaron *et al*, 2020; Bouton *et al*, 2023). We have investigated select members of these highly effective virus‐derived immune‐modulating proteins as a source for new anti‐inflammatory therapeutics designed to treat damaging inflammation and coagulopathy and to investigate molecular mechanisms driving ARDS (Chen *et al*, 2013; Yaron *et al*, 2020). Serp‐1 is a myxoma virus‐derived secreted 55 kDa serpin glycoprotein that operates in virus‐infected tissues by protecting the virus against activated myeloid cells (Nash *et al*, 1997). Serp‐1 treatment improves outcomes in gamma herpesvirus‐induced vasculitis and lung infection (Chen *et al*, 2013), pristane‐induced diffuse alveolar hemorrhage (DAH; Guo *et al*, 2021), and numerous animal models of vascular injury and allograft transplants (Lucas & McFadden, 2004; Chen *et al*, 2013; Yaron *et al*, 2020). Purified Serp‐1 protein is a species‐nonspecific inhibitor of systemic inflammation, binding and inhibiting multiple activated serine proteases in the thrombotic and thrombolytic (coagulation) and complement pathways (Lomas *et al*, 1993). Systemic treatment with purified Serp‐1 downregulates trafficking of migratory macrophages and lymphocytes into damaged tissues via binding to uPAR (Dai *et al*, 2006; Viswanathan *et al*, 2009). uPAR (CD87) is a glycosylphosphatidylinositol (GPI)‐linked surface protein that sits in a large lipid raft of proteins that interact with integrins, chemokine receptors, vitronectin, growth factors, low‐density lipoprotein receptor‐related protein (LRP), and complement receptors on the cell surface, altering cell activation and motility (Fleetwood *et al*, 2014). Serp‐1 also binds and inhibits tPA, thrombin, fX, and several complement proteases as determined by immunoprecipitation and mass spectrometry in a mouse model of autoimmune lupus lung hemorrhage (Guo *et al*, 2021). A Serp‐1 RCL mutant has Ala‐Ala replacing the P1‐P1′ scissile‐bond Arg‐Asn and is inactive in transplant and angioplasty models (Lucas *et al*, 1996; Dai *et al*, 2006). Bolus systemic treatment with clinical grade Serp‐1 has also proved safe and effective in a Phase IIA randomized, blinded, dose‐escalating clinical trial, performed at seven sites in the United States and Canada, in unstable coronary syndrome patients with stent implants (Tardif *et al*, 2010), reducing early markers for heart damage, troponin (TN), and creatinine kinase MB (CK‐MB), with minimal adverse events at the therapeutic dose (15 µg/kg).

A modified PEGylated serpin, PEGSerp‐1 protein therapeutic has now been developed with improved half‐life and activity (Guo *et al*, 2021). PEGSerp‐1 binds uPA, tPA, and thrombin (Appendix Fig S1; Guo *et al*, 2021), and complement pathway C1 (q, r, and s), factor B, and C3 and C4 proteases (Appendix Table S1). PEGSerp‐1 represents a new class of immune‐modulating virus‐derived biologic with proven efficacy in lupus lung hemorrhage (Guo *et al*, 2021) and muscular dystrophy (Andre *et al*, 2022). In both virus infection and lung hemorrhage models, serpin treatment altered expression of coagulation and complement pathway proteases and serpins (Chen *et al*, 2013; Guo *et al*, 2021).

Here, we assess the efficacy of systemic PEGSerp‐1 treatment to target immune and coagulation pathways and treat severe SARS‐CoV‐2 viral lung infections in two mouse‐adapted models, MA10 in BALB/c mice (Leist *et al*, 2020) and MA30 in C57BL/6 mice (Wong *et al*, 2022).

## Results

### PEGSerp‐1 treatment of MA30‐infected C57Bl/6 mice

**Figure 1:**
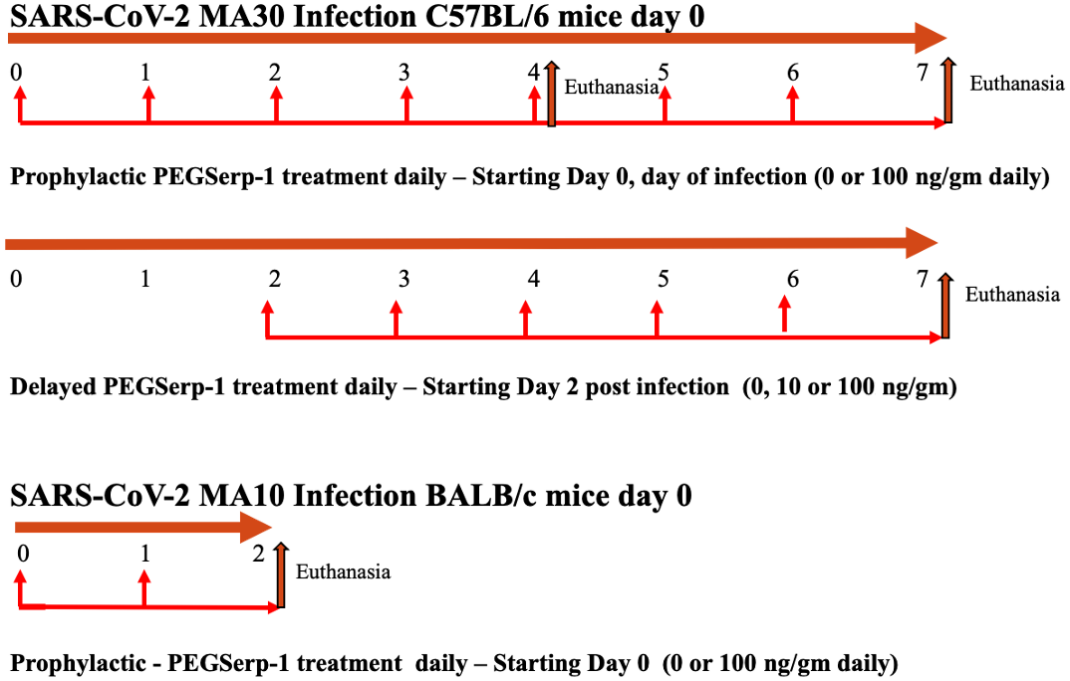
Flow chart for PEGSerp‐1 treatment in SARS‐CoV‐2‐infected mouse models Top—Flow chart for SARS‐CoV‐2 MA30 infection studies in C57BL/6 mice with prophylactic saline control or PEGSerp‐1 treatment (100 ng/gm) given daily starting on the first day of infection with 4 and 7 days follow‐up (*N* = 40). Mid—Flow chart for delayed treatments in SARS‐CoV‐2 MA30‐infected C57Bl/6 mice (0, 10, or 100 ng/gm doses) starting 2 days after infection with 7 days follow‐up (*N* = 24). Bottom—Flow chart for the MA10 SARS‐CoV‐2 infection study in BALB/c mice, with PEG‐Serp‐1 treatments given daily starting on the first day of infection and early follow‐up at 2 days post‐infection.

**Figure 2:**
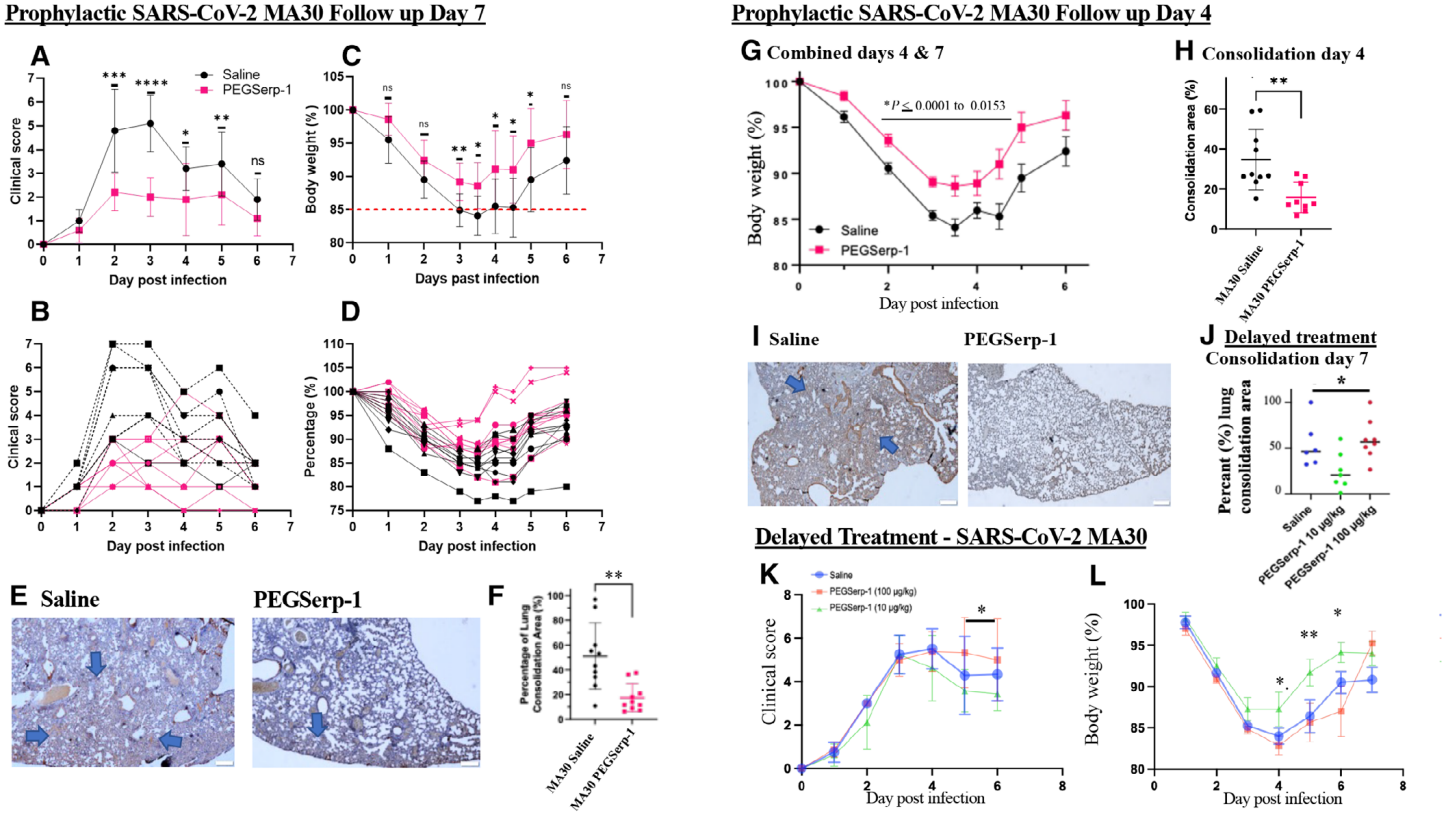
PEGSerp‐1 treatment improves clinical score and weight gain and reduces lung consolidation in SARS‐CoV‐2‐infected C57Bl/6 mouse models A–DSARSCoV‐2 MA30 infection in C57BL/6 mice 7 days follow‐up. PEGSerp‐1 treatment significantly improved mean (A) and individual (B) clinical scores (*P* < 0.045 to 0.0001, days 2–5) as well as mean (C) and individual weights (D) (*P* < 0.0481–0.0085, days 3–5; *N* = 20 mice, A, C mean ± SD).EMicrographs illustrate lung consolidation in saline and PEGSerp‐1‐treated lungs. Large blue arrows indicate areas of consolidation in H&E‐stained lung sections (Mag 2×).FGraph of (F) mean consolidation area divided by total lung area analyzed at 7 days follow‐up demonstrates reduced lung consolidation (*P* < 0.0018). PEGSerp‐1 treatment significantly improved weight gain during the second 4 day follow‐up study. (*N* = 20 mice, mean ± SE)G, HCombined weights for the 4‐ and 7‐day follow‐up studies demonstrate consistently improved mean weights (G; *P* < 0.0153–0.0001) and reduced lung consolidation (H; *P* < 0.0033) with PEGSerp‐1 treatments in MA30‐infected mice. (*N* = 39mice, mean ± SD)IMicrographs illustrate lung consolidation divided by total section analyzed in saline and PEGSerp‐1‐treated MA30‐infected mice at 4 days follow‐up. (Red—PEGSerp‐1, Black—saline) Large blue arrows indicate areas of consolidation in H&E‐stained lung sections (Mag 2×).J–LPEGSerp‐1 delayed treatment at 10 ng/gm starting 2 days after infection reduced lung consolidation (J; *P* < 0.0403), clinical scores (K, combined data days 5 and 6, *P* < 0.05), and weight loss (L; *P* < 0.001–0.05). (*N* = 24 mice, mean ± SE) Data analysis information: Mean ± SD (A, C, Mean ± SE (H, J, K, L); *P < 0.05, **P < 0.01, ***P < 0.001, ****P < 0.0001; (A–D, F) Unpaired *t*‐tests; (J) ANOVA indicated by line at top with subgroup analyses in brackets below; Prophylactic treatment (A–D) and (F); black—saline, red—PEGSerp‐1 treatments. (J–L) Blue—saline, green and blue—PEGSerp‐1. Source data are available online for this figure.

PEGSerp‐1 treatment was assessed in SARS‐CoV‐2 MA30‐infected C57Bl/6 mice using a lethal dose 50 (LD50) inoculation calculated at 1.3–1.4 × 10^3^ pfu (Fig 1; Wong *et al*, 2022). MA30‐infected mice were treated with daily intraperitoneal (IP) injections of either PEGSerp‐1 or control saline and followed for either 4 or 7 days (*N* = 40 mice, Table 1). With 7 days follow‐up (*N* = 20 mice), MA30‐infected mice treated with saline control displayed clinical symptoms of MA SARS‐CoV‐2 infection with respiratory distress (Fig 2A and B) and weight loss (Fig 2C and D) on days 2 to 5 post‐infection followed by gradual improvement on day 6 (the clinical score was a combined scoring for weight loss, hunching, ruffling/sneezing, and labored breathing; adapted from Moreau *et al*, 2020). PEGSerp‐1 treatment significantly improved systemic responses in this SARS‐CoV‐2 MA30 infection model in C57Bl/6 mice when compared to saline control treatments, with improved clinical score and reduced weight loss in infected mice on blinded analysis (Fig 2A–D; with a range of statistical scores from *P* < 0.045–0.001), together with significantly reduced weight loss on days 3–5 with PEGSerp‐1 treatments (Fig 2C and D; *P* < 0.0481–0.0083) (Fig 2A and C represent mean values for each treatment group; B and D represent the individual values measured for individual mice). Overall efficacy for PEGSerp‐1 treatment was greatest at 3–5 days after infection. Lung consolidation was significantly reduced with PEGSerp‐1 treatments (Fig 2E and F) as measured by mean area of lung consolidation divided by total lung area assessed in stained sections, calculated for multiple reads and sections per mouse (Fig 2F; *P* < 0.0018). Lung consolidation was defined as loss of open alveolar spaces due to leukocyte infiltration with associated clotting and/or bleeding.

**Table 1:** SARS‐CoV‐2 mouse infection models.

SARS‐CoV‐2 infection	Mouse strain	Mouse age	Number of mice	Infection pfu	Treatment	Follow‐up times	Loss
SARS‐C0V‐2 MA10	BALB/c	12 weeks	2	None	Saline d0	48 h	0
SARS‐C0V‐2 MA10	BALB/c	12 weeks	6	5 × 10^5^ pfu	Saline d0	48 h	0
SARS‐C0V‐2 MA10	BALB/c	12 weeks	2	None	PEGSerp‐1 d0 (100 ng/gm)	48 h	0
SARS‐C0V‐2 MA10	BALB/c	12 weeks	6	5 × 10^5^ pfu	PEGSerp‐1 d0 (100 ng/gm)	48 h	0
SARS‐C0V‐2 MA30	C57Bl/6	10 weeks	10	1.3 × 10^3^ pfu	Saline d0	4 days	0
SARS‐C0V‐2 MA30	C57Bl/6	10 weeks	10	1.3 × 10^3^ pfu	PEGSerp‐1 d0 (100 ng/gm)	4 days	1
SARS‐C0V‐2 MA30	C57Bl/6	10 weeks	10	1.3 × 10^3^ pfu	Saline d0	7 days	0
SARS‐C0V‐2 MA30	C57Bl/6	10 weeks	10	1.3 × 10^3^ pfu	PEGSerp‐1 d0 (100 ng/gm)	7 days	0
SARS‐C0V‐2 MA30	C57Bl/6	10 weeks	8	1.3 × 10^3^ pfu	Saline d2	7 days	2
SARS‐C0V‐2 MA30	C57Bl/6	10 weeks	8	1.3 × 10^3^ pfu	PEGSerp‐1 d2 (10 ng/gm)	7 days	1
SARS‐C0V‐2 MA30	C57Bl/6	10 weeks	8	1.3 × 10^3^ pfu	PEGSerp‐1 d2 (100 ng/gm)	7 days	4

The second cohort of mice, assessed at 4 days follow‐up (*N* = 20 mice) with and without PEGSerp‐1 treatment, provided a second independent analysis of PEGSerp‐1 treatment during SARS‐CoV‐2 MA30 infection, with an earlier timepoint for lung pathology assessment (Fig 2G–I). PEGSerp‐1 treatment again reduced weight loss on days 2 and 3 after infection (Appendix Fig S2; *P* < 0.0074–0.0138). To assess for variability in the model, combined data were analyzed for both day 4 and day 7 follow‐up studies and demonstrated preserved significance, further supporting a consistent benefit with PEGSerp‐1 treatment (Fig 2G; *P* < 0.0001 to 0.0153). Reduced weight loss for the day 4 follow‐up group with PEGSerp‐1 treatment was also associated with a significant reduction in lung consolidation on histopathological assessment, corroborating the prior 7‐day follow‐up study (Fig 2H and I; *P* < 0.0033). No mice died during the 7‐day follow‐up study. One mouse died immediately upon intranasal (IN) inoculation of SARS‐CoV‐2 in this second group due to anesthesia complications prior to receiving treatment.

In a third cohort of SARS‐CoV‐2 MA30‐infected C57BL/6 mice, PEGSerp‐1 treatment was given at doses of 0, 10, or 100 ng/gm (0, 10, or 100 μg/kg) starting 2 days after initial virus infection (*N* = 24, 8 mice per treatment group; Fig 2J–L). The 10 ng/gm dose is comparable to the effective 15 μg/kg (15 ng/gm) doses given in the Phase 2 clinical trial of Serp‐1 treatment in unstable coronary patients post‐stent implant (Tardif *et al*, 2010). With this delayed PEGSerp‐1 treatment, 10 ng/gm doses demonstrated greater benefit with significantly improved weight and clinical scores at 4–6 days post‐infection (Fig 2K and L; *P* < 0.0351 to 0.0421). Higher‐dose PEGSerp‐1 (100 ng/gm) did not significantly affect weight loss although there was a trend toward increased weight gain in the last 2 days (*P* = 0.07, ns). Area of lung consolidation was reduced on histologic analysis at the effective treatment dose with improved clinical score and weight loss (Fig 2J, *P* < 0.0403). Although the MA30 infection was initiated with an intended LD50 inoculum, infection‐related deaths were only observed in the third study with delayed treatment, 7/24 mice died with SARS infection in this group beginning at 4 days post‐infection. One mouse in the prophylaxis group died on day 0 when first inoculated with virus due to excess volume and respiratory distress (1/40). Overall, there was no significant change in mortality with treatment in either early or delayed PEGSerp‐1 treatment (*P* = 0.28).

The results overall from the two cohorts of MA30‐infected mice with PEGSerp‐1 prophylactic treatment starting on the day of infection indicate consistent, significantly improved weight gain and clinical scores at days 2–5 and at days 4 to 6 post‐infection for delayed treatment. With delayed treatment in infected mice with established viremia, the lower‐dose PEGSerp‐1 treatment significantly improved weight gain and reduced the clinical score. Lung consolidation area was reduced in mice where PEGSerp‐1 improved weight gain and clinical score.

### Inflammatory cell invasion in lungs—effects of PEGSerp‐1 treatment in SARS‐CoV‐2 MA30‐infected C57BL/6 mice

**Figure 3:**
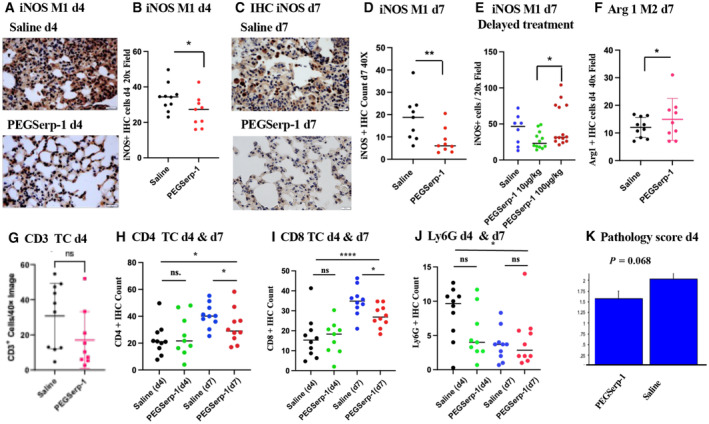
PEGSerp‐1 treatment significantly reduced immune cell infiltrates in SARS‐CoV‐2 MA30‐infected C57BL/6 mouse models at 4 and 7 days follow‐up (*N* = 39 mice) A, BMicrographs illustrating iNOS‐positive M1 macrophage cell infiltrates on IHC‐stained lung sections in saline and PEGSerp‐1 (A)‐treated mice with significantly reduced IHC‐positive cell counts at 4 days follow‐up after PEGSerp‐1 prophylactic treatment (B; *P < 0.045). IHC‐stained lung sections (Mag 40×).C, DiNOS‐positive infiltrates in IHC‐stained lung sections with saline or PEGSerp‐1 (C) treatment with significantly reduced IHC‐positive cell counts at 7 days follow‐up with prophylactic PEGSerp‐1 treatment (D; **P < 0.0052). IHC‐stained lung sections (Mag 40×).EDelayed PEGSerp‐1 10 ng/gm dose treatment given 2 days after infection also reduced iNOS‐positive cell counts (*P < 0.0368).FArginase1‐positive M2 macrophage IHC cell counts demonstrate a trend toward an increase at 4 days (*P* = 0.2921) and a significant increase at 7 days (*P < 0.0414) with PEGSerp‐1 treatment.GCD3^+^ T cell counts were not significantly altered at 4 days (*P* = 0.1058).HCD4^+^ T cell counts are not reduced at 4 days (*P* = 0.5031) but are reduced at 7 days (*P < 0.0454) with PEGSerp‐1 treatments.ICD8‐positive T cell counts on IHC‐stained sections have a trend toward an increase at 4 days (*P* = 0.1503) and a significant reduction at 7 days (*P < 0.0303) with PEG Serp‐1 treatments.JIHC for neutrophil marker Ly6G did not detect significant changes in PEGSerp‐1 treatment at days 4 (*P* = 0.0860) and 7 (*P* = 0.6570).KIndependent blinded pathology score indicates a nonsignificant trend toward reduced inflammation at 4 days follow‐up (*P* = 0.068). Data information: Mean ± SE; *P < 0.05, **P < 0.01, ***P < 0.001, ****P < 0.0001; ANOVA indicated by line at top with subgroup analyses in brackets below; back and blue circles—saline, red and green circles—PEGSerp‐1 treatments. Source data are available online for this figure.

Cellular immune responses were examined in MA30 SARS‐CoV‐2 infection models by immunohistochemical (IHC) analysis to measure neutrophil, macrophage and T cell infiltrates in infected lung sections. Pro‐inflammatory M1 macrophage iNOS‐positive cell counts were significantly reduced with prophylactic PEGSerp‐1 treatments on day 4 (Fig 3A and B; *P* < 0.045) and day 7 (Fig 3C and D; *P* < 0.0052) follow‐up, as measured on lung micrograph sections. A greater reduction in iNOS‐positive cell counts was detected at 7 days. With delayed PEGSerp‐1 treatments starting 2 days post‐infection, iNOS‐positive macrophage counts were similarly reduced at the effective 10 ng/gm daily dose (Fig 3E; *P* < 0.0368). Arg1‐positive M2 macrophage cell counts were not altered on day 4 (*P* = 0.2921), but were significantly increased on day 7 (Fig 3F; *P* < 0.0414) in MA30‐infected mice with prophylactic PEGSerp‐1 treatment, consistent with a progressive anti‐inflammatory effect. With delayed treatments, Arg 1 counts were not significantly increased with PEGSerp‐1 treatments (*P* = ns). CD3‐positive T cell staining indicated a non‐significant trend toward a reduction (Fig 3G; *P* = 0.1058). CD4 staining (Fig 3H; ANOVA *P* < 0.0138) was not altered on day 4 with PEGSerp‐1 treatment (*P* = 0.5013), but CD4^+^ T cells were significantly reduced by PEGSerp‐1 treatment on day 7 in MA30‐infected mice (*P* < 0.0454). There was a nonsignificant trend toward an increase in CD8‐positive T cells (Fig 3I, ANOVA *P* < 0.0001) at day 4 (*P* = 0.1503), but at 7 days follow‐up, CD8 T cells were significantly reduced in PEGSerp‐1‐treated infected mice (*P* < 0.0303), similar to trends observed with CD4‐stained cells. With delayed PEGSerp‐1 treatments, CD3‐positive cell counts were not significantly altered (*P* = 0.2717). Neutrophil counts, measured by the Ly6G marker, had a non‐significant decrease in PEGSerp‐1‐treated infected mice on day 4 (Fig 3J, *P* = 0.086), and no change on day 7 (*P* = 0.6570). An independent blinded histopathological analysis detected a trend toward a decrease in the overall pathology score at day 4 follow‐up (Fig 3K, *P* = 0.068).

### Inflammatory cell invasion in lungs—effects of PEGSerp‐1 treatment in SARS‐CoV‐2 MA10‐infected BALB/c mice

**Figure 4:**
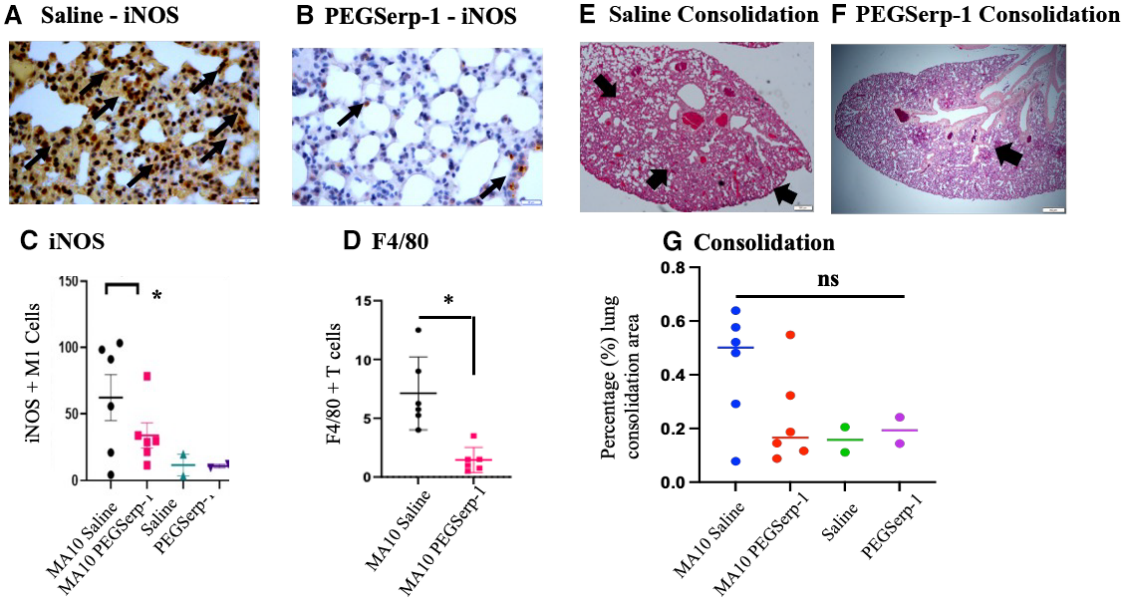
SARS‐CoV‐2 MA10‐infected BABLB/c mice at 48 h follow‐up (*N* = 16 mice) A–GPEGSerp‐1 treatment reduced lung consolidation and iNOS + macrophage infiltration. iNOS M1‐stained macrophage invasion in saline control‐treated mice (A) is significantly reduced with PEGSerp‐1 treatment (B). Graphs illustrate significant reductions in mean iNOS‐positive M1 macrophage (C; *P < 0.05) and F4/80 macrophage (D; *P < 0.012)‐positive stained cell counts. H & E‐stained histology sections of lung illustrating consolidation in saline‐treated mice (E) is reduced by PEGSerp‐1 treatment (F). Graph of measured consolidation area divided by total lung area (G; *P* = 0.1073). PEGSerp‐1‐treated and saline‐treated uninfected mouse tissues had similar levels of inflammation and consolidation (E, G). Mean for positively stained cell counts in three high‐power fields (HPF) per mouse. Black arrows point to brown iNOS‐positive macrophage on IHC‐stained lung sections (Mag 40×). Data information: (Mean ± SE; *P < 0.05; ANOVA indicated by line at top with subgroup analyses in brackets below; back and blue circles—saline, red and green circles—PEGSerp‐1 treatments). Source data are available online for this figure.

C57Bl/6 and BALB/c mice have differing immune responses; C57BL/6 mice are TH1 polarized and BALB/c are TH2 polarized, with associated change in macrophage responses (Watanabe *et al*, 2004). Serpin treatment has been previously associated with altered macrophage and T cell responses, and in some cases with M2 polarization (Munuswamy‐Ramanujam *et al*, 2010; Zhang *et al*, 2019). Thus, as a secondary screen, PEGSerp‐1 treatment was assessed in SARS‐CoV‐2 MA10‐infected BALB/c mice to assess serpin treatment efficacy in a second mouse strain with differing immune responses (*N* = 16, Table 1; Leist *et al*, 2020). PEGSerp‐1 treatment was given prophylactically on the day of infection with 48 h follow‐up. iNOS‐positive M1 macrophage cell counts were significantly reduced in the MA10 SARS‐CoV‐2‐infected mice treated with PEGSerp‐1 at 48 h follow‐up (Fig 4A–C; *P* < 0.05). On IHC analysis, both iNOS‐positive M1 macrophage counts (Fig 4C; *P* < 0.05) and F4/80‐positive macrophage counts (Fig 4D; *P* < 0.012) were significantly reduced in infected lungs by PEGSerp‐1 treatment. Lung sections from uninfected controls with saline or PEGSerp‐1 treatments had minimal areas of iNOS‐positive macrophage cell counts (Fig 4E). Lung consolidation was reduced in uninfected mice and SARS‐CoV‐2 MA10‐infected mice with PEGSerp‐1 treatments (Fig 4F and G), but this reduction at 2 days follow‐up, albeit a trend toward a reduction, was not significant (*P* = 0.1073, Fig 4G). PEGSerp‐1‐treated mice with SARS‐CoV‐2 MA10 infection had similar mean levels of consolidation as uninfected mice (Fig 4G). At this early follow‐up time, the SARS‐CoV‐2 MA10 model in BALB/c mice produced histological evidence for lung infection but did not produce significant weight loss or clinical symptoms (scores) (Leist *et al*, 2020).

### Inflammatory cell invasion in myocardium and vasculature—effects of PEGSerp‐1 treatment in SARS‐CoV‐2‐infected mice

**Figure 5:**
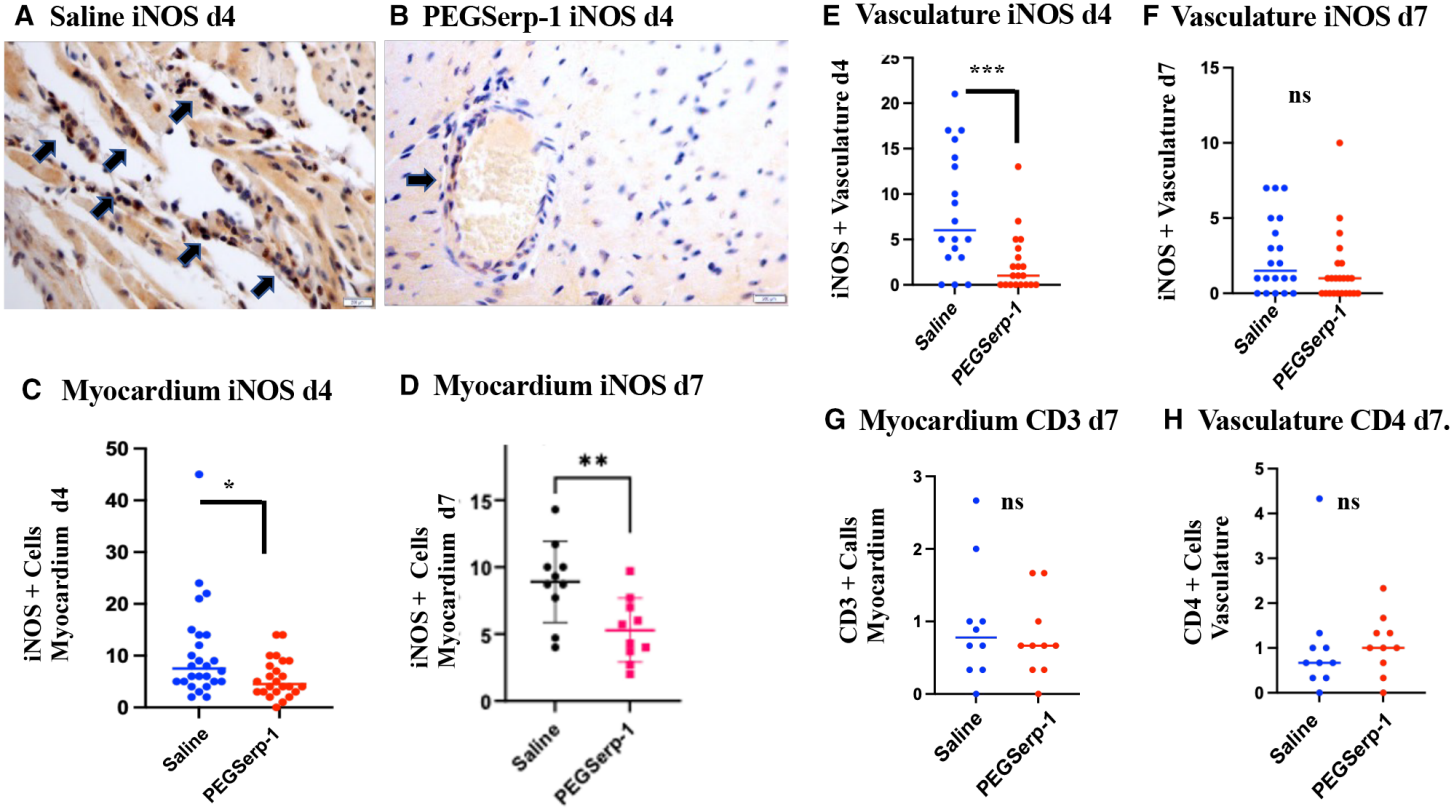
Myocardium and vascular staining detect reduced overall iNOS‐positive M1 macrophage cell counts on IHC micrographs of myocardium in PEGSerp‐1‐treated SARS‐CoV‐2 MA30‐infected C57BL/6 mice A, BMicrographs illustrating iNOS‐positive cells in saline‐ (A) and PEGSerp‐1‐ (B) treated mice at 4 days follow‐up. IHC‐stained sections 40×.C–HGraphs illustrate significantly reduced mean iNOS‐positive cell counts in the myocardium at 4 days (C; *P < 0.0238) and 7 days (D; **P < 0.0082). iNOS‐positive perivascular cell counts are significantly reduced at 4 days (***E; *P* < 0.001), but not 7 days follow‐up (F; *P* = 0.156). CD3 IHC staining in the myocardium (G; *P* = 0.5481) and the vasculature (H; *P* = 0.9404) is not significantly changed with PEGSerp‐1 treatments. Graphs illustrate mean values for positively stained cells counts on IHC‐stained sections Data information: Mean ± SE; *P < 0.05, **P < 0.01, ***P < 0.001; Student's *t*‐test; back and blue circles—saline, red and green circles—PEGSerp‐1 treatments). Source data are available online for this figure.

The entire cardiovascular system has been associated with widespread inflammatory immune cell responses and micro‐thrombotic vascular occlusions during SARS‐CoV‐2 infections in humans. We examined the effects of SARS‐CoV‐2 MA30 infections in hearts isolated from infected C57Bl/6 mice, with and without PEGSerp‐1 treatments, at days 4 and 7 post‐infection. Inflammation was detected in the myocardium and associated heart vasculature, but was less prominent than inflammation detected in the lungs of SARS‐CoV‐2‐infected mice with saline control treatments (Fig 5). Inflammatory cell infiltrates were detectable in the myocardium in isolated pockets often in the pericardium or leaflets and perivascular spaces.

PEGSerp‐1 significantly reduced detectable iNOS‐positive M1 macrophage cell counts in the myocardium at 4 (Fig 5A–C; *P* < 0.0238) and 7 days (Fig 5D; *P* < 0.0082) follow‐up. In the small vessels in the myocardium, iNOS‐positive M1 macrophage counts were significantly decreased at day 4 (Fig 5E and F; *P* < 0.001), but not on day 7 (Fig 5F, *P* = 0.156). CD3‐positive T cell counts were not significantly reduced by PEGSerp‐1 at day 7 in the myocardium (Fig 5G, *P* = 0.5482) or in the vasculature (*P* = 0.113) of virus‐infected mice. CD4^+^ T cell counts were also not significantly altered by PEGSerp‐1 in the myocardium or vasculature (Fig 5H; *P* = 0.6408 and 0.9404, respectively). Ly6G‐positive cell counts were not significantly changed by PEGSerp‐1 in the myocardium or vasculature.

### Immunohistochemical analyses of uPAR, fX, fibrinogen, and complement in the lungs, heart, and vasculature

As a serpin, PEGSerp‐1 binds to the uPA/uPAR complex (Appendix Fig S1) as well as factor X and fibrinogen in the clotting cascades (Lomas *et al*, 1993; Nash *et al*, 1997; Dai *et al*, 2006; Viswanathan *et al*, 2009; Guo *et al*, 2021). PEGSerp‐1 also binds a wide range of complement cascade proteases, as demonstrated on mass spectrometry pull down in a hemorrhagic lung model (Guo *et al*, 2021; Appendix Table S1). uPAR is commonly upregulated and expressed on the leading edge of migratory leukocytes and can also be cleaved and released into the blood as soluble uPAR (suPAR; Dai *et al*, 2006; Viswanathan *et al*, 2009; Fleetwood *et al*, 2014; Yu *et al*, 2019; Yu & Liu, 2019; D'Alonzo *et al*, 2020; Acanfora *et al*, 2021; Arnold *et al*, 2021; de Bruina *et al*, 2021; Guo *et al*, 2021; Keskinidou *et al*, 2021; Zuo *et al*, 2021; The PHOSP‐COVID Collaborative Group, 2022) and has been reported as a marker for severe COVID‐19 with higher mortality. Thus, increased uPAR expression at the cell surface may be reflected in elevated cleaved or soluble (suPAR). Changes in detectable uPAR and C5b‐9 membrane attack complex (MAC) levels were measured by IHC analysis of lung sections from SARS‐CoV‐2‐infected mice treated with PEGSerp‐1.

**Figure 6:**
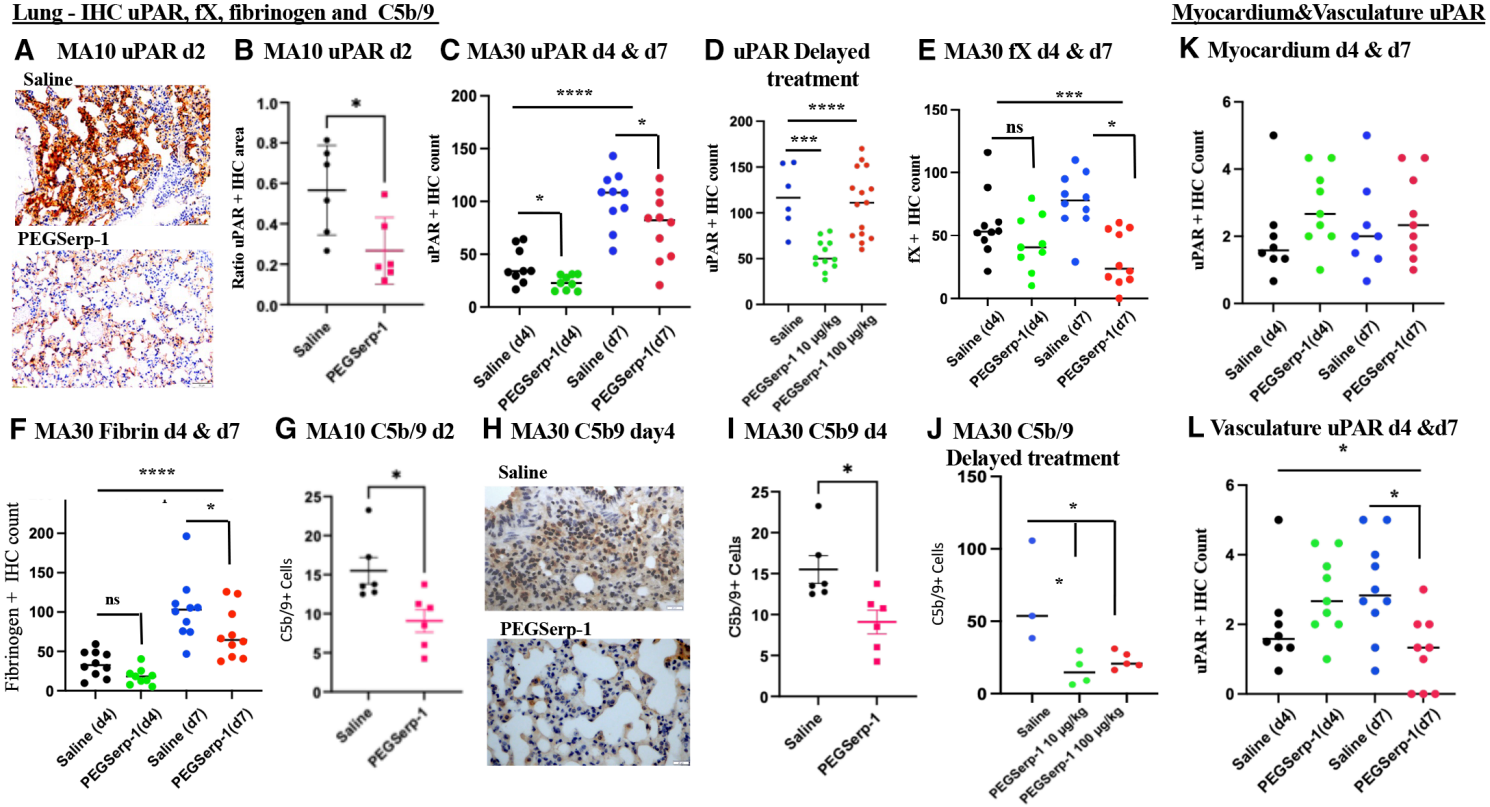
IHC‐stained sections demonstrate reduced uPAR, fX, fibrinogen, and C5b‐9 (MAC) staining with PEGSerp‐1 treatment in MA10‐infected BALB/c (*N* = 16 mice) and MA30‐infected C57BL/6 mouse models (*N* = 39 mice prophylactic treatment, *N* = 24 mice delayed treatment) A–GPEGSerp‐1‐treated mouse lung sections from MA10 at day 2 follow‐up have significant reduction in uPAR‐positive stained areas (A) with reduced positively stained hemorrhagic areas in lungs at 2 days in MA10‐infected BALB/c (B; *P* < 0.041) and reduced uPAR + stained cell counts at 4 days (C; *P* < 0.0202) and 7 days (C in alveoli; *P* < 0.0148) in MA30‐infected C57BL/6 mice. With delayed treatments, uPAR is reduced at 7 days follow‐up (D; ANOVA *P* < 0.0001; *P* < 0.0002 for PEGSerp‐1 at 10 μg/kg). FX staining was reduced with prophylactic PEGSerp‐1 at day 7 but not at day 4 follow‐up (E; ANOVA *P* < 0.0008; *P* < 0.0001 for day 7, and *P* = 0.5148 for day 4). Fibrinogen was similarly reduced at day 4 with PEGSerp‐1 treatment but not at day 7 (F; ANOVA *P* < 0.0002; *P* = 0.1653 day 4; *P* < 0.019 day 7). C5b‐9 MAC‐positive staining is reduced with PEGSerp‐1 treatment in MA10‐infected BALB/c mice (G; *P* < 0.0165).H–LC5b‐9 staining is reduced in prophylactic PEGSerp‐1 treatment in MA30‐infected C57BL/6 mice on IHC‐stained sections (H) with significant reductions at day 4 (I; *P* < 0.0114), and a nonsignificant trend at day 7 follow‐up (*P* = 0.5694). With delayed PEGSerp‐1 treatment, C5b/9 detection on IHC is significantly reduced by both PEGSerp‐1 doses (J; *P* < 0.0131). In the myocardium, uPAR detection at day 4 (K; *P* = 0.2675) and 7 (K; *P* = 0.2002) is not significantly changed with PEGSerp‐1 treatment. Whereas in the small vessels in the heart, uPAR has a trend toward a reduction at day 4 (L; *P* = 0.0847) and a significant reduction at day 7 (L; *P* < 0.0082). Data information: Mean ± SE. *P < 0.05, ***P < 0.001, ****P < 0.0001; ANOVA indicated by line at top with subgroup analyses in brackets below; back and blue circles—saline, red and green circles—PEGSerp‐1 treatments. Source data are available online for this figure.

Detectable uPAR was reduced with PEGSerp‐1 treatments in the lungs of MA10‐infected BALB/c mice at 2 days follow‐up (Fig 6A and B; *P* < 0.041) and at days 4 and 7 in SARS‐CoV‐2 MA30‐infected C57Bl/6 mice (Fig 6C; *P* < 0.0202 on day 4 and *P* < 0.010 on day 7). Comparison of alveolar and bronchial staining was equivalent for the inhibitory effects of PEGSerp‐1 treatment on detected levels of cellular uPAR (*P* < 0.001 for reduced uPAR in bronchi). The hemorrhagic areas of lung consolidation in control virus‐infected mice were also heavily stained for uPAR and this was similarly reduced by PEGSerp‐1 treatment, as might be expected given the fact that PEGSerp‐1 binds uPA as a competitive inhibitor. uPAR detection was significantly reduced with delayed treatments with 10 ng/gm PEGSerp‐1 doses (Fig 6D; ANOVA, *P* = 0.0001; *P* = 0.0002 for PEGSerp‐1 10 ng/gm compared to saline). Detectable changes in thrombotic pathway proteases were also assessed. No change in factor X (fX) staining (Fig 6E; ANOVA *P* < 0.0008) was seen at 4 days with prophylactic PEGSerp‐1 treatments (*P* = 0.5148), but fX was significantly reduced at 7 days (Fig 6E, *P* < 0.0001). A reduction in detectable fibrinogen was also seen at days 4 and 7 with PEGSerp‐1 treatment, with significance at 7 days (Fig 6F; *P* = 0.1653 day 4; *P* < 0.019 day 7).

At days 2 and 4 follow‐up in MA10 and MA30 SARS‐CoV‐2‐infected mice, respectively, there was also a significant reduction in detected C5b‐9 membrane attack complex (MAC) on IHC staining in infected mice receiving prophylactic PEGSerp‐1 treatment (Fig 6G, *P* < 0.0165) on day 2 in MA10 infection and on day 4 in MA30 infection (Fig 6H and I; *P* < 0.0056). At day 7 in MA30‐infected mice, however, the reduction in C5b/9 MAC staining with prophylactic PEGSerp‐1 treatment in MA30‐infected mice was no longer significant (*P* = 0.1201). With delayed PEGSerp‐1 treatment, a significant reduction in detectable C5b/9 was seen at both doses (Fig 6J; ANOVA *P* < 0.0001; *P* < 0.0131 for 10 μg/kg doses).

uPAR and C5b‐9 staining were similarly assessed in the myocardial and vascular tissues in prophylactic treatment of SARS‐CoV‐2‐infected mice. There is a nonsignificant trend toward an increase in detectable uPAR staining at day 4 and day 7 follow‐up in the myocardium (Fig 6K; *P* = 0.2675 and *P* = 0.2002, respectively). There is a nonsignificant trend toward a decrease in uPAR in the small vessels (Fig 6L, ANOVA *P* = ns), in myocardial sections at d4 (Fig 6L; *P* = 0.0687), however, uPAR detection was significantly reduced in the myocardial vessels at day 7 (Fig 6L; *P* < 0.0295). On analysis of C5b9 detection in the myocardium of SARS‐CoV–infected mice, PEGSerp‐1 treatment was associated with significantly reduced positive staining at day 4 (*P* < 0.0114), but not at 7 days (*P* < 0.5694). This suggests that reductions in uPAR occur later in the myocardium and associated vessels, than in the lungs as might be predicted based on the known route of SARS‐CoV‐2 infection. Detected change in C5b9 MAC IHC staining was reduced earlier in the myocardium at 4 days with PEGSerp‐1 treatment than at 7 days.

### Immunohistochemical staining for viral N and S proteins in mice infected with MA10 or MA30

**Figure 7:**
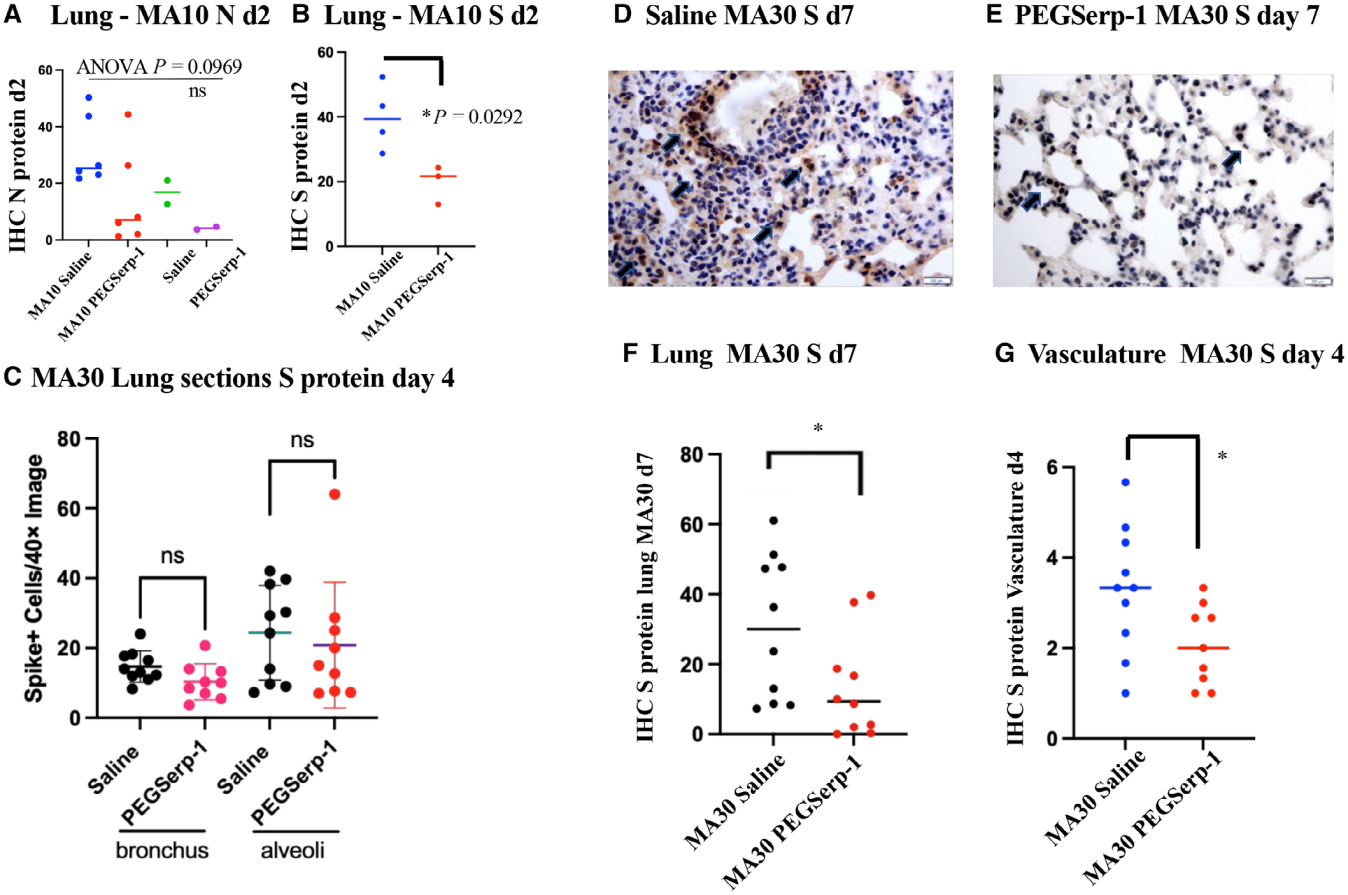
Reduced S protein was detected on IHC of lung samples with PEGSerp‐1‐treated SARS‐CoV‐2 MA10‐infected BALB/c mice (*N* = 16) and SARSCoV‐2 MA30 (*N* = 39)‐infected C57BL/6 mice AN protein is increased in MA10‐infected, saline control‐treated mice with a nonsignificant trend toward a reduction with PEGSerp‐1 treatment (*P* = 0.0969).BS protein is significantly reduced in MA10‐infected mice after PEGSerp‐1 prophylactic treatment (*P* < 00292)CPEGSerp‐1 treatment is not associated with reduced detectable S protein on IHC staining at 7 days in MA300infected mice (C; *P* < 0.5105).D–GIn MA30‐infected C57BL/6 mice, increased S protein is seen on IHC staining seen in lung sections from saline‐treated mice (D). S protein staining is reduced with PEGSerp‐1 treatment at 7 days follow‐up (E, F; *P* < 0.001) and in the small vessels (G; *P* < 0.0364). Data information: Mean ± SE. *P < 0.05, **P < 0.01, ***P < 0.001, and ****P < 0.0001; ANOVA indicated by line at top with subgroup analyses in brackets below. Source data are available online for this figure.

The effect of PEGSerp‐1 treatment on detectable virus after infection was also examined by IHC staining for SARS‐CoV‐2 spike (S) and nucleocapsid (N) proteins in lung tissues in infected mice. As expected, detectable S and N protein levels on IHC‐stained sections were increased in virus‐infected mice when compared to uninfected control mice (Fig 7). N protein levels were not reduced with prophylactic PEGSerp‐1 treatment in MA10‐infected mice at 2 days follow‐up, although there was a nonsignificant trend toward a reduction (Fig 7A; *P* = 0.0969). Significant reductions in staining for S protein were detected in MA10‐infected mice at 2 days (Fig 7B; *P* < 0.0292) and in MA30‐infected mice at day 7 in lung sections, but not at day 4 (Fig 7C–F; *P* = 0.5101 at day 4 and *P* < 0.05 at day 7) with PEGSerp‐1 treatments. Detectable S protein staining was not reduced in the myocardium of SARS‐CoV‐2 MA30‐infected mice (*P* = 0.8666), however, detected S was significantly reduced in the vasculature on IHC analysis at 4 days follow‐up (Fig 7G; *P* < 0.0364).

### Analysis of gene expression in the lung during SARS‐CoV2 infections

**Figure 8:**
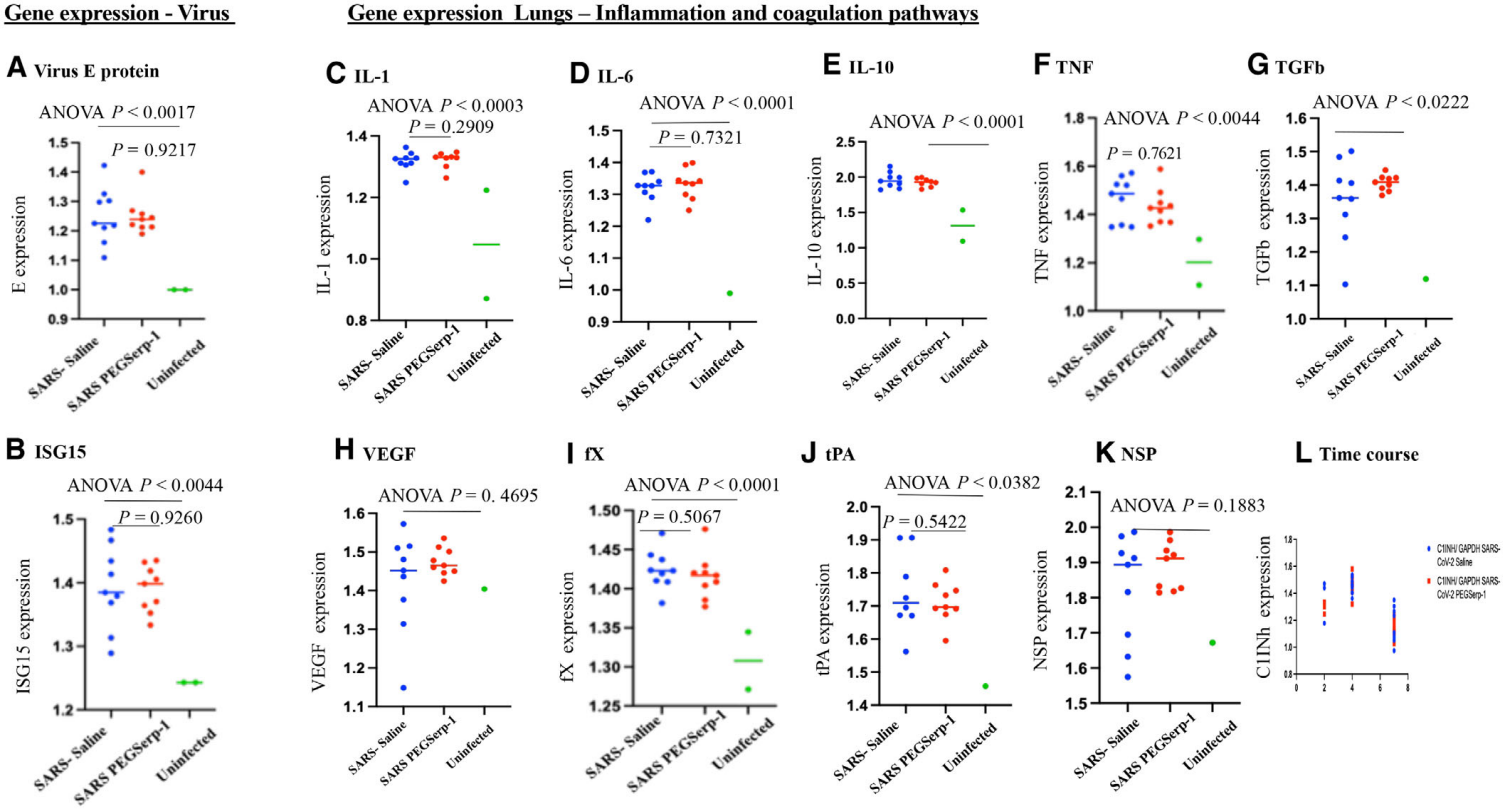
Gene expression—qPCR virus, inflammatory, and coagulation pathways. Relative changes in gene expression for SARS‐CoV‐2 infections and associated modulation in inflammatory and coagulation responses are increased after SARS‐CoV‐2 MA30 infection in lung and heart tissues (SARSCoV‐2 MA30 infection—*N* = 18 mice 4 days follow‐up; *N* = 20 mice 7 days follow‐up; uninfected mice—*N* = 2). PEGSerp‐1 treatment significantly modified gene expression for uPAR and complement pathways A, BRelative gene expression is significantly increased for SARS‐CoV‐2 MA30 viral E gene (A; *P* < 0.0017) and interferon response ISG15 (B; *P* < 0.0044) in saline‐treated infected lung extracts at 4 days follow‐up, when compared to uninfected mouse lungs.C–LInflammatory cytokines and growth factors are significantly increased with infection; IL‐1 (C; *P* < 0.0003), IL‐6 (D *P* < 0.0001), IL‐10 (E; *P* < 0.0001), TNF (F; *P* < 0.0044), and TGFb (G; *P* < 0.0022) are increased with SARS‐CoV‐2 infection at 4 days follow‐up; with VEGF as the exception (H; *P* = 0.4695). Factors in the coagulation pathway, fX (I; *P* < 0.0001), tPA (J; *P* < 0.0382), and neuroserpin (K; *P* = 0.1883) are also increased. None of these markers are modified by PEGSerp‐1 treatments. Data information: Gene expression is normalized to GAPDH expression. Y‐axes vary depending on the detected relative expression levels after normalizing data to the internal control. (ANOVA indicated by line at top with subgroup analyses below; infected mice—blue circles saline, red circle—PEGSerp‐1 treatments, and green circle—uninfected mice.) Source data are available online for this figure.

The transmembrane protease, serine 2 (TMPRSS2) protein cleaves the S protein on SARS‐CoV‐2 after binding to the ACE2 receptor, enabling viral fusion and entry at the cell surface. To examine the effects of PEGSerp‐1 treatments on SARS‐CoV‐2 virus infection, gene expression was measured by quantitative RT–PCR (qRT–PCR) array for SARS‐CoV‐2 viral envelope (E) and nucleocapsid (N) gene expression in extracts from frozen mouse lung tissue at day 4 in MA30‐infected mice. Increased expression of E (Fig 8A; *P* < 0.0017) and N genes (*P* < 0.0044) were detected in all SARS‐CoV‐2 MA30‐infected mice at 4 days follow‐up, when compared to uninfected lung isolates. There was a corresponding significant increase in the canonical interferon‐stimulated gene‐15 (ISG15) in MA30 infected mice compared to uninfected lung tissues (Fig 8B; *P* < 0.0044). However, PEGSerp‐1 treatment did not alter detectable levels of SARS‐CoV‐2 E gene expression (Fig 8A; *P* = 0.9217) or N (*P* = 0.4138), or the virus‐induced upregulation of ISG15 gene expression at 4 days follow‐up (Fig 8B; *P* = 0.9260). This suggests that PEGSerp‐1 is not directly impacting the virus cell entry and replication, but instead targeting, as expected, the inflammatory response to infection.

To assess gene expression in the acute response coagulation and inflammatory pathways, further qRT–PCR array analysis was performed for representative genes in SARS‐CoV‐2 MA30‐infected lung samples, in parallel with uninfected lung tissues. There was a general increase in expression of inflammatory response genes at 4 days after SARS‐CoV‐2 MA30 infection when compared to uninfected mouse lung samples. Gene expression in inflammatory cell response pathways was significantly elevated for interleukin‐1 (IL‐1; Fig 8C; *P* < 0.0003), IL‐6 (Fig 8D; *P* < 0.0001), IL‐10 (Fig 8E; *P* < 0.0001), tumor necrosis factor (TNF; Fig 8F; *P* < 0.0044), and transforming growth factor beta (TGFb; Fig 8G; *P* < 0.0222), but not for vascular endothelial growth factor (VEGF; Fig 8H; *P* = 0.4695) in infected mouse lung tissues when compared to uninfected mice. In the coagulation pathways, significant increases were seen for factor X (fX; Fig 8I; *P* < 0.0001) and tPA (Fig 8J; *P* < 0.0382), but not for neuroserpin (NSP, Fig 8K; *P* = 0.1883) in the lungs of virus‐infected mice. When examining qPCR at 2, 4 and 7 days follow‐up, the greatest increase in gene expression with viral infection alone is seen at 4 days (Fig 8L, C1Inh illustrated).

**Figure 9:**
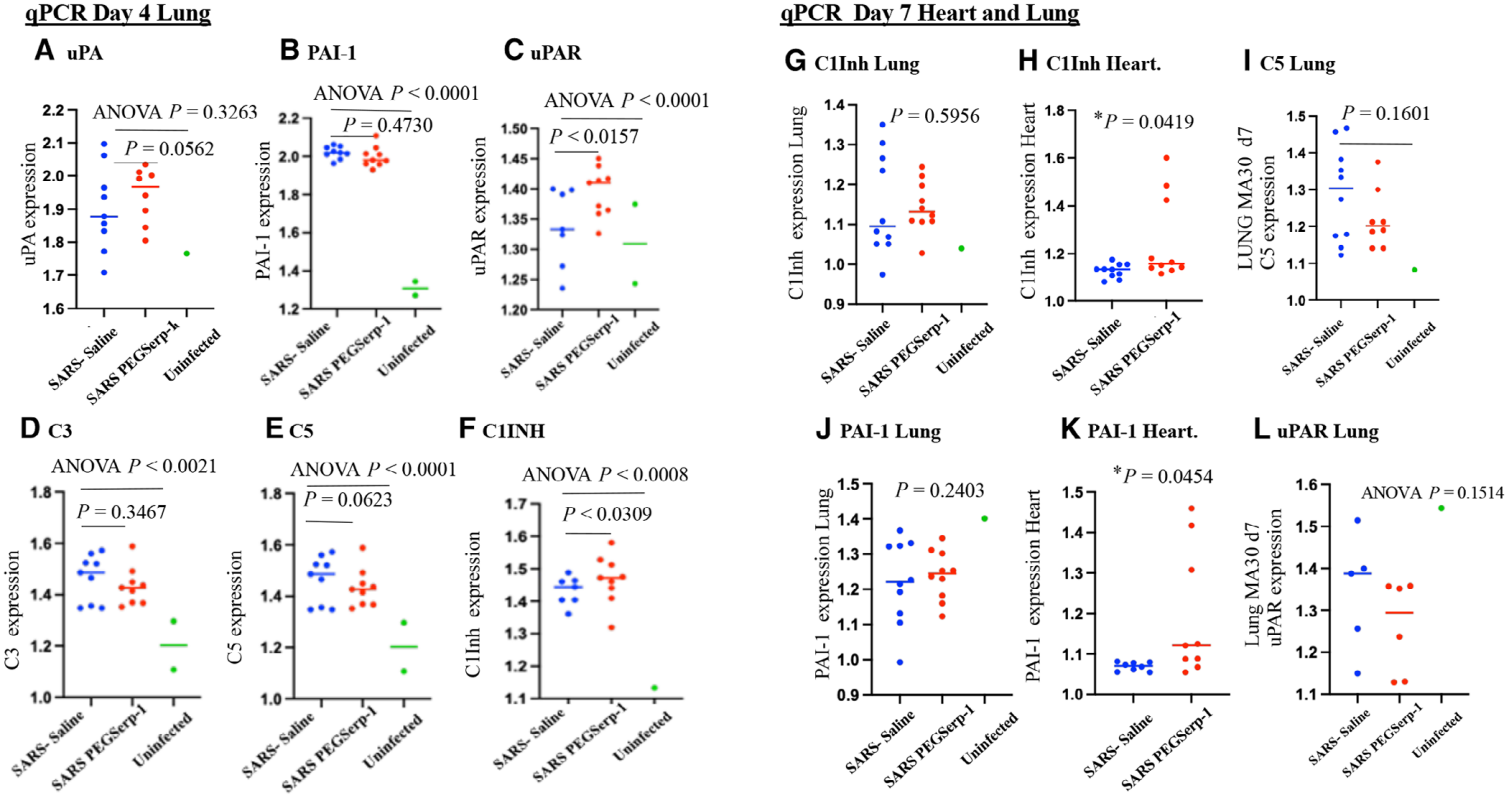
Gene expression—qPCR uPA/uPAR and complement pathways. Relative changes in gene expression for SARS‐CoV‐2 infections and associated modulation in coagulation and immune responses are increased after SARS‐CoV‐2 MA30 infection in lung and heart tissues (SARSCoV‐2 MA30 infection—*N* = 18 mice 4 days follow‐up; *N* = 20 mice 7 days follow‐up; Uninfected mice—*N* = 2). PEGSerp‐1 treatment significantly modified gene expression for uPAR and complement pathways A–CGene expression for PEGserp‐1 targets are increased after infection and modified by PEGSerp‐1 treatments. uPA (A; *P* = 0.3263), PAI‐1 (B; *P* < 0.0001), and uPAR (C; *P* < 0.0001) were increased in infected mouse lung extracts at 4 days follow‐up when compared to uninfected lungs with SARS‐CoV‐2 MA30 infection. PEG Serp‐1 treatment produced a significant increase in uPAR (C; *P* < 0.0157).D–FComplement pathway genes‐encoding C3 (D; *P* < 0.0021), C5 (E; *P* < 0.001), and C1Inh (F; *P* < 0.0008) were also significantly increased with infection. PEG Serp‐1 treatment produced a borderline decrease in C5 (E; *P* = 0.0623) and a significant increase in C1Inh (F; *P* < 0.0309).G–IAt 7‐day follow‐up, relative gene expression is no longer increased for C1Inh with PEGSerp‐1 treatment in MA30‐infected mouse lungs (G; *P* = 0.5956), but C1Inh was significantly increased in myocardial extracts with PEGSerp‐1 treatment at 7 days (H; *P* < 0.0419). C5 was not significantly increased with infection (I; *P* = 0.1601)J–LPAI‐1 gene expression was not increased in lung extracts with PEGSerp‐1 treatment at 7 days follow‐up in MA30‐infected mouse lungs (J; *P* = 0.2403), but PAI‐1 expression was significantly increased at 7 days follow‐up in heart extracts (K; *P* < 0.0454). uPAR was not significantly increased in lung extracts at 7 days follow‐up (*P* = 0.1514). A time course for gene expression illustrates a peak time for changes in C1Inh gene expression demonstrates greater changes at 4 days when compared to day 2 and day 15 (L). Data information: *N* = 19 mice at 4 days follow‐up; *N* = 20 mice at 7 days follow‐up. ANOVA indicated by line at top with subgroup analyses below; infected mice—blue circles, saline; red circle, PEGSerp‐1 treatments; and green circle, uninfected mice). Source data are available online for this figure.

To further assess known targets for PEGSerp‐1 in the thrombotic, thrombolytic, and immune pathways, the uPA/uPAR and complement pathways, where reductions were detectable on IHC for PEGSerp‐1 treatment, gene expression for uPA, uPAR, and plasminogen activator inhibitor‐1 (PAI‐1, SERPINE1), as well as C3, C5, and complement 1 inhibitor (C1Inh, SERPING1), were assessed on qPCR (Fig 9). No significant change was detectable in uPA (Fig 9A; *P* = 0.3263), but significant increases in PAI‐1 (Fig 9B; *P* < 0.0001) and uPAR (Fig 9C; *P* < 0.0029) gene expression were detected with viral infection in control‐treated samples at day 4. With viral infection alone, in control samples, C3 (Fig 9D; *P* < 0.0021), C5 (Fig 9E; *P* < 0.0001), and C1Inh (Fig 9F; *P* < 0.0008) were significantly increased.

PEGSerp‐1 significantly and unexpectedly increased uPAR gene expression (Fig 9C; *P* < 0.0157), produced a borderline decrease in C3 (Fig 9D; *P* = 0.3467) and C5 (Fig 9E; *P* = 0.0623), and significantly increased C1Inh (Fig 9F; *P* < 0.0309). PEGSerp‐1 treatment was not associated with a change in fX gene expression (Fig 8I; *P* = 0.5067). Nonsignificant inhibitory trends were observed for TNF and other inflammatory markers with PEGSerp‐1 treatments (Figs 8 and 9). The increased uPAR and the reductions in C3 and C5 with increases in C1Inh (Fig 9F; *P* < 0.0309) support a greater effect of PEGSerp‐1 treatment on the uPAR and complement pathways in the lungs of virus‐infected mice.

Formalin‐fixed paraffin‐embedded (FFPE) lung samples from the SARS‐CoV2 MA10 (48 h) and the SARS‐coV‐2 MA30 (7 day) were available and were also assessed for gene expression changes with SARS‐CoV‐2 MA10 (48 h) and SARS‐CoV‐2 MA30 (Day 7) infections. Heart samples were additionally available for the day 7 SARS‐CoV‐2 MA30 samples. The MA10 SARS‐CoV‐2 MA10 infection lung samples, 48 h, were limited and sample numbers too small to provide statistically meaningful findings in the MA10 infection samples. However, there is an apparent association between inflammatory markers and time of follow‐up with greater increases overall at day 4 over day 2 and then a subsidence of levels by day 7 for several markers, consistent with the overall changes in clinical score and weight loss with SARS‐CoV‐2 infections (Fig 8L). For the day 7 FFPE samples, we saw similar trends in gene expression as seen in the day 4 samples with PEGSerp‐1 treatments in MA30‐infected mice. C5 was again reduced with PEGSerp‐1 treatments at day 7, with a nonsignificant trend (Fig 9I; *P* = 0.1601), whereas there was a borderline significant change in day 4 follow‐up samples (Fig 9E; *P* = 0.0623). uPAR, which was significantly increased at 4 days with PEGSerp‐1 treatment (Fig 9C, *P* < 0.0157), was no longer increased at 7 days follow‐up (Fig 9L; *P* = 0.1514). C1Inh, which was significantly increased with PEGSerp‐1 treatments at 4 days in the lungs (Fig 9F; *P* < 0.0309), was no longer significantly increased at 7 days in lung sections (Fig 9G; *P* = 0.5956).

Heart tissues in contrast had a significant increase in day 7 cardiac samples for serpin markers after MA30 infection while lung samples showed a reduction (Fig 9G–L). In the cardiac samples, and of interest for serpin treatments, in the day 7 follow‐up, heart tissue samples C1Inh was significantly increased on qPCR array analysis with PEGSerp‐1 treatments (Fig 9H; *P* < 0.0419). Similarly, the PAI‐1 mammalian serpin, while no longer increased in lung samples (Fig 9J; *P* = 0.2403), was now increased in cardiac samples at day 7 follow‐up (Fig 9K; *P* < 0.0454). uPAR and C5 were not significantly altered in the heart sections on day 7 (*P* = 0.2184 and *P* = 0.1617, respectively). This suggests that PEGSerp‐1 treatment modified gene expression of uPAR and complement pathways with associated changes in regulatory serpins in both lung and heart tissues with differing activation time windows.

## Discussion

With this study, we demonstrate a significant improvement in clinical scores, weight gain, and lung damage during systemic treatment with PEGSerp‐1, a myxoma virus‐derived serpin, in two different mouse‐adapted models of SARS‐CoV‐2. This unique serpin targets activated serine proteases in both coagulation and complement pathways, reducing immune cell invasion in the lung and heart. SARS‐CoV‐2 infections begin in the nasopharynx and quickly migrate to the lungs with subsequent invasion of other visceral organs, with a damaging immuno‐coagulopathic reaction. Parallel changes in both heart and lungs of PEGSerp‐1‐treated mice indicate a systemic and sequential reduction in immune responses to SARS‐CoV‐2 infection. Inflammatory M1 macrophage infiltrates were significantly reduced with PEGSerp‐1 treatments during infection in both MA10‐infected BALB/c mice (Leist *et al*, 2020) and in MA30‐infected C57Bl/6 mice (Wong *et al*, 2022) when given prophylactically or as delayed treatments. M2‐activated macrophages as well as CD4 and CD8 T cell responses were modified later during MA30 infections. C57BL/6 and BALB/c mice have marked differences in their immune response patterns to virus infections (i.e., C57‐derived mice tend to be TH1 polarized, whereas BALB/c‐derived mice are more TH2 polarized) (Watanabe *et al*, 2004). Thus, PEGSerp‐1 suppressed SARS‐CoV‐2 virus‐induced pathologic immune reactions in multiple mouse genetic backgrounds. These findings are consistent with prior reported improved lung inflammation, vasculitis, and associated improved survival in Serp‐1‐treated MHV68 gamma herpesvirus‐infected mouse model (Lomas *et al*, 1993; Nash *et al*, 1997; Chen *et al*, 2013; Yaron *et al*, 2020). Additionally, in a pristane‐induced lupus alveolar hemorrhage model, PEGSerp‐1 treatment improved pathologic lung hemorrhage and reduced macrophage invasion (Guo *et al*, 2021) when given 7 days after inducing lung damage. Purified, unmodified Serp‐1 protein expressed in CHO cells has also been tested and proven safe and effective in a randomized Phase IIa clinical trial for treating cardiovascular patients with unstable coronary disease and stent implant. In this clinical study markers for heart damage, troponin T and creatinine kinase MB were significantly reduced (MACE of zero and minimal antigenicity with no detected significant neutralizing antibodies; Tardif *et al*, 2010). PEGSerp‐1 treatment thus has improved outcomes in models for both RNA and DNA viral lung infections as well as for immune‐coagulopathic lung damage.

Balancing activation of the thrombotic/thrombolytic and complement pathways via serpin treatment has the potential to restore homeostasis in severe viral infections where there is aggressive and damaging activation of immune and coagulation proteases. PEGSerp1 is a myxoma poxvirus‐derived serpin developed to high efficacy through millions of years of virus evolution to protect the virus from activated inflammatory cellular attack by the host (Lomas *et al*, 1993; Nash *et al*, 1997; Lucas & McFadden, 2004; Dai *et al*, 2006; Tardif *et al*, 2010; Chen *et al*, 2013; Zhang *et al*, 2019; Yaron *et al*, 2020; Guo *et al*, 2021). Serp‐1 targets multiple thrombotic, thrombolytic, and immune pathways activated in severe viral lung infections and septic states with the potential to restore homeostatic balance in excessively activated uPAR and complement pathways. Key serine proteases in each of these molecular pathways are regulated by endogenously expressed mammalian serpins (Lomas *et al*, 1993; Nash *et al*, 1997; Bouton *et al*, 2023). Serpins are suicide inhibitors that target activated serine proteases and act as enzyme pseudo‐substrates at sites of immune and coagulation pathway activation. These protease pathways are routinely activated during severe virus infections (Papageorgiou *et al*, 2018; Levi *et al*, 2020; Levi & Thachil, 2020; Cugno *et al*, 2021;Wettstein *et al*, 2021; Karnaukhova, 2022). Two mammalian serpins, alpha 1 antitrypsin (A1AT, SERPINA1), and complement 1 inhibitor (C1Inh, SERPING1) are in clinical trials as potential treatments for SARS‐CoV‐2 ARDS (Wettstein *et al*, 2021; Karnaukhova, 2022). Anti‐thrombin III (AT, SERPINC1) was previously investigated as a treatment for bacterial sepsis and associated disseminated intravascular coagulation (DIC). AT treatment exhibited a trend toward improved outcomes in bacterial sepsis, but did not result in statistical significance, whereas C1Inh treatment has early benefit (Levi *et al*, 2020; Levi & Thachil, 2020; Karnaukhova, 2022).

Inflammatory cell invasion and increased pro‐inflammatory gene expression were both detected in SARS‐CoV‐2 virus‐infected mouse lung tissue when compared to normal uninfected lung tissues. PCR array analyses demonstrated a clear and significant increase in viral gene expression, as well as interferon mediators such as ISG15 and a range of both thrombotic/thrombolytic and immune pathways in the lungs of MA SARS‐CoV‐2‐infected mice. Both IHC and qRT–PCR analyses indicated that PEGSerp‐1‐targeted uPAR in the thrombolytic pathway, and C5 and C1Inh in the complement pathway which were significantly altered.

Mechanistically, Serp‐1 anti‐inflammatory activity is dependent on the uPA receptor (uPAR) (Lomas *et al*, 1993; Macen *et al*, 1993; Lucas *et al*, 1996; Dai *et al*, 2006; Viswanathan *et al*, 2009; Munuswamy‐Ramanujam *et al*, 2010; Zhang *et al*, 2019; Yaron *et al*, 2021) and Serp‐1 loses all therapeutic effects in uPAR‐knockout mice in transplant models (Dai *et al*, 2006) and with antibody to uPAR given topically to dermal wounds in mice (Zhang *et al*, 2019). The uPA/uPAR complex activates plasmin and matrix metalloproteinases (MMPs), acting both as a thrombolytic protease and an immune modulator that regulates macrophage and neutrophil activation and tissue invasion (Yu *et al*, 2019; Yu & Liu, 2019; D'Alonzo *et al*, 2020; Acanfora *et al*, 2021; Zuo *et al*, 2021). Of interest, the effects of PEGSerp‐1 treatment on gene expression in the lungs of MA SARS‐Cov‐2‐infected mice were more diverse than expected: uPAR and C1Inh both increased, while C5 was borderline reduced. In the heart, both PAI‐1, a uPA‐regulating mammalian serpin, and C1Inh were increased with PEGSerp‐1 treatment. In prior work with the MHV‐68 gamma herpesvirus infection model, there was also significant increase in serpins that target the uPA pathway (Chen *et al*, 2013). Serp‐1 binding to uPAR on the surface of activated and mobilized macrophages has been demonstrated with potential to alter cellular activation through interactions with uPAR lipid rafts, perhaps accounting for a feedback‐mediated increase in uPAR gene expression (Viswanathan *et al*, 2009). The PEGSerp‐1‐induced changes in complement gene expression may be more consistent with reduced levels of lung inflammation and tissue damage. These analyses were performed on whole lung isolates, inclusive of lung epithelium, arterial endothelium, and invasive immune cells, and may or may not represent changes in protein expression and activity in either infiltrating immune cells or resident lung cells (Lomas *et al*, 1993, Appendix Table S1).

The SARS‐CoV‐2 spike (S) protein binds to cellular ACE2 receptors and during entry at the cell surface is cleaved at the S2’ site by the surface transmembrane protease serine 2, TMPRS2, which promotes fusion of the viral envelope with the cell membrane (Yu *et al*, 2019; Yu & Liu, 2019; D'Alonzo *et al*, 2020; Leist *et al*, 2020; Keragala & Medcalf, 2021; Li *et al*, 2021; Zuo *et al*, 2021; Wong *et al*, 2022). The S protein may be cleaved by circulating thrombotic proteases, thrombin, and factor X, as well as the thrombolytic protease plasmin (Yu *et al*, 2019; Yu & Liu, 2019; D'Alonzo *et al*, 2020; Acanfora *et al*, 2021; Zuo *et al*, 2021). A reduction in S protein was observed on IHC‐stained sections in PEGSerp‐1‐treated MA‐infected mice. Our qRT–PCR analyses demonstrated a clear increase in SARS‐CoV‐2 E and N gene expression in the lungs of SARS‐CoV‐2 MA30‐infected C57Bl6 mice, but no change was seen in PEGSerp‐1‐treated animals. Thus, reduced detectable S protein with IHC staining is likely an indirect result of the serpin‐mediated amelioration of lung damage and inflammation and we postulate that PEGSerp‐1 therapeutic effect is primarily anti‐inflammatory and not anti‐viral.

Severe SARS‐CoV‐2 viral lung infection is associated with ARDS, especially when patients reach the ICU. The mammalian serpin, plasminogen activator inhibitor‐1 (PAI‐1, SERPINE1) binds uPA and tPA and levels are elevated in SARS‐CoV‐2‐infected patients (Cugno *et al*, 2021; Zuo *et al*, 2021). Anti‐thrombin (AT, SERPINC1) has been investigated as a treatment for severe bacterial sepsis and DIC (Papageorgiou *et al*, 2018; Levi *et al*, 2020; Levi & Thachil, 2020). AT produced a trend toward benefit, but did not achieve significance for bacterial sepsis. Other clinical trials are in progress examining the mammalian serpins, alpha1 antitrypsin (A1AT, SERPINA1) (Wettstein *et al*, 2021), and C1 esterase inhibitor (C1‐Inh, SERPING1) (Karnaukhova, 2022) as treatments for severe SARS‐CoV‐2, both with a range of serine protease inhibitory functions. A later reduction in fibrinogen and fX was detected in lung sections on IHC analysis at 7 days follow‐up with PEGSerp‐1 treatment, but not at earlier 4 days follow‐up.

Immune‐mediated damage and widespread micro‐thrombotic occlusions (coagulopathy) cause severe lung and multiorgan damage in severe respiratory or mosquito‐borne viral pandemics, including dengue, influenza, Ebola, and SARS‐CoV‐2. Treatments for the early viremic phase of COVID‐19 have advanced with selectively targeting monoclonal antibodies and anti‐viral therapeutics, but there is a need for more effective immune modulators during the later‐stage pro‐inflammatory phase of the disease (Nicholls *et al*, 2003; Kobasa *et al*, 2007; Maines *et al*, 2009; Memoli *et al*, 2009; Bunce *et al*, 2011; Calore *et al*, 2011; Tate *et al*, 2011; Yu *et al*, 2011, 2019; Nakajima *et al*, 2012; von Ranke *et al*, 2013; Teijaro, 2015; Papageorgiou *et al*, 2018; Bradley & Bryan, 2019; Yu & Liu, 2019; Angus *et al*, 2020; D'Alonzo *et al*, 2020; Levi *et al*, 2020; Levi & Thachil, 2020; Maldonado *et al*, 2020; The RECOVERY Collaborative Group, 2020; Acanfora *et al*, 2021; Aiyegbusi *et al*, 2021; Arnold *et al*, 2021; Cugno *et al*, 2021; de Bruina *et al*, 2021; Jordan, 2021; Keskinidou *et al*, 2021; Kurtovic & Beeson, 2021; Mir *et al*, 2021; Novelli *et al*, 2021; Perico *et al*, 2021; Wettstein *et al*, 2021; Yong, 2021; Zuo *et al*, 2021; Karnaukhova, 2022; The PHOSP‐COVID Collaborative Group, 2022; Wang *et al*, 2022). Drugs targeting pro‐inflammatory cytokines such as IL‐1 and IL‐6, as well as JAK pathway inhibitors, are effective, but mortality for high‐risk, hypoxic patients remains high.

Dysregulation of both the thrombotic and thrombolytic serine protease pathways co‐activates excessive innate immune responses and complement cascades further damaging cells and increasing systemic thrombosis. Activation of uPAR (especially as assessed by the upregulation of circulating cleaved suPAR) and the complement cascade are markers for progression‐to‐severe COVID‐19 complications. Mammalian serpins represent 2–10% of circulating blood proteins, including AT, AAT, and C1Inh, all of which regulate thrombosis and inflammation. Highly potent serpin‐based immune regulators have also evolved in viruses over millions of years selectively targeting sites of host serine protease activation that normally help combat pathogens during infection (Lomas *et al*, 1993; Nash *et al*, 1997; Lucas & McFadden, 2004; Yaron *et al*, 2020). Of interest, the serpins are inhibitors that target active proteases, honing to sites of protease activation allowing for lower effective doses (μg/kg) with postulated targeting to sites of immune and coagulation dysfunction (Bouton *et al*, 2023). Thus, the poxvirus‐derived Serp‐1 protein evolved in myxoma virus as a secreted inhibitor of activated myeloid cells. Activated macrophages more effectively recognize and clear myxoma virus from infected tissues when the viral Serp‐1 gene is deleted (Macen *et al*, 1993; Lucas & McFadden, 2004; Bouton *et al*, 2023).

Modified expression of detectable uPAR and complement on the IHC analyses, as well as in analysis of gene expression for uPA and uPAR in the lungs and C5 complement together with C1Inh in lung sections and C1Inh and PAI‐1 in heart samples, suggests that these may be targeted pathways for PEGSerp‐1 efficacy in the SARS‐CoV‐2 model. A reduction in fX and fibrinogen in the clotting cascades is seen but occurs at later times suggesting again uPAR and complement as PEGSerp‐1 targets. With delayed PEGSerp‐1 treatment, reduced uPAR is only detected at doses effective in reducing clinical findings, whereas C5b‐9 staining was reduced at both doses potentially suggesting a closer correlation for uPAR than complement MAC inhibition with clinical benefits in mice. The protein interactome as demonstrated in prior work in a lung hemorrhage model with MS analysis supports a central role for these pathways as targets for PEGSerp‐1‐mediated immuno‐coagulopathic modulation and efficacy (Guo *et al*, 2021) (Guo *et al*, 2021; Appendix Table S1, Fig S1). The increases in gene expression are interesting and may represent a feedback mechanism. These findings suggest a potential for broad interaction with serine proteases in the coagulation and complement pathways but will require more extensive analyses in the SARS‐CoV‐2 mouse models.

Treatments given prophylactically on the day of infection demonstrated improved weight gain and clinical score. With PEGSerp‐1 treatment delayed for 2 days, benefit was again detected at the lower dose, comparable to the effective dose given in a prior clinical trial. To fully assess the potential benefit for treatment in clinical ARDS and for PEGSerp‐1 to restore homeostasis and reduce immune coagulopathic complications will require longer‐term follow‐up with survival analysis as the murine models used compress the early viremic stage with the later inflammatory phase seen in humans, as well as assessment of a broader range of doses. To fully investigate the mechanism of action of PEGSerp‐1 will also require further studies with mouse models deficient in uPAR and complement. This work is nevertheless supported by prior work with uPAR‐deficient aortic allograft transplant models in mice where Serp‐1 activity was blocked when uPAR was deleted.

In this study, we have examined treatment of two mouse‐adapted SARS‐CoV‐2 models using PEGSerp‐1, a pegylated version of the viral Serp‐1 protein, a serpin that targets multiple serine proteases in the coagulation and complement protease cascades. Treatment significantly improved clinical scores, decreased lung inflammation and consolidation, and modified uPAR, thrombotic proteases, and complement levels. Overall, the study described here for SARS‐CoV‐2, and with earlier studies in other models for severe viral infection, strongly support further studies and development of PEGSerp‐1 as a new anti‐inflammatory therapeutic or biologic for advanced virus‐induced lung and vascular damage, designed to target coagulation disorders, micro‐thrombi, hemorrhage, and damaging inflammation. Longer‐term studies are needed to assess the impact of PEGSerp‐1 treatment on severe viral ARDS, immune‐coagulopathic complications, and chronic infections that may contribute to chronic debilitating disease, as seen in long COVID following SARS‐CoV‐2 infection.

## Materials and Methods

### SARS‐CoV‐2 infection

All animal studies conformed to local and national guidelines for animal care and experimentation and were approved by the Institutional Animal Care and Use Committee (IACUC) of Arizona State University (ASU) (#20‐1761R). Animals were housed in barrier conditions at the ASU Animal Care Services vivarium and bred under specific pathogen‐free conditions. Equal numbers of male and female mice were used for all studies. Mice were weaned at 3 weeks, maintained on a 12‐h light–dark cycle, and fed water and standard rodent chow *ad libitum*. Mouse cohorts were transferred from the ABSL1 colony to a separate ABSL2 colony until beginning work in the ABL3 facility. C57BL/6J mice were purchased from the Jackson Laboratory and BALB/c mice were bred in‐house. Mice were infected at 8–10 weeks of age. For prophylactic treatment, C57BL/6 or BALB/c mice were treated on the day of SARS‐CoV‐2 MA30 or MA10 infection and treated daily with intraperitoneal injection (IP) of either saline control or PEGSerp‐1, 100 ng/gm body weight with treatment continued throughout the course of infection to follow‐up at 4 or 7 days post‐infection. For the delayed treatments, C57Bl/6 mice were infected with SARS‐CoV‐2 MA30 and treated with saline, 10 ng/gm, or 100 ng/gm PEGSerp‐1 starting 2 days after infection and daily to follow‐up at 7 days post‐infection (initial starting doses were continued for the study duration). Mice were weighed daily and assessed for clinical signs using a clinical score (adapted from Moreau *et al*, 2020). For each MA30 study, BSL3 treatments and the histopathology were blinded to treatment. For the MA10 study, the histology analysis was blinded. For euthanasia, mice were deeply anesthetized with an overdose of 80 mg sodium pentobarbital/kg body weight. Lung and heart were harvested after euthanizing. Tissues for pathology analyses were post‐fixed in formalin and tissues for qPCR were frozen for Trizol.

For MA10 infection 2 × 10^5^ pfu was inoculated by intranasal route (IN) in BALB/cAnNCrl (Leist *et al*, 2020). For MA30 infection, 1.3 × 10^3^ pfuvirus was inoculated IN in C57Bl/6 mice as previously described (Wong *et al*, 2022). For IN instillation, mice were sedated prior to infection with 50 mg/kg ketamine and 7.5 mg/kg xylazine and infected IN with virus in 5 μl DMEM, as previously described (Wong *et al*, 2022). Mice were placed in home cages, which were placed on heating pads, and monitored until ambulatory. Mice that fell below 85% of original weight were checked and weighed twice daily. Animals are monitored for activity, posturing, respiratory distress, decreased appetite, and weight loss in comparison to control animals as warning markers for potential need to euthanize the test animals earlier than the projected dates. The weights are scored as a “1” for 90–99%, “2” for 80–89%, and “3” for under 80%. The weight score is added to scores for hunching, ruffling/sneezing, lethargy, and labored breathing. Mice reaching a score that indicated an endpoint were humanely euthanized.

### Viruses and cells

- For SARS‐CoV‐2 MA10 infection**—**mouse‐adapted SARS‐CoV‐2 MA10 was obtained from Dr. Ralph Baric at the University of North Carolina at Chapel Hill (Leist *et al*, 2020). MA10 virus stock was grown in Vero‐TMPRSS2 cells that stably express TMPRSS2 and were obtained from Dr. Stefan Pohlmann at the University Gottingen, Germany.
- For SARS‐CoV‐2 MA30 infection—Mouse‐adapted SARS‐CoV‐2 SARS2‐N501Y_MA30_ (LD 50–1.3 × 10^3^ pfu) (obtained from Dr. Stanley Perlman at the University of Iowa) was propagated in A549‐huACE2 cells (Li *et al*, 2021). Human A549 cells (verified by ATCC) stably express human ACE2. Cells were cultured in RPMI 1640 (Gibco catalog No. 11875) supplemented with 10% FBS, 100 U/ml of penicillin, and 100 μg/ml streptomycin.

### PEGSerp‐1—PEGylation and protein purification

Serp‐1 (m008.1L; NCBI Gene ID# 932146) was expressed in a Chinese hamster ovary (CHO) cell line (Lucas lab; Dai *et al*, 2006; Tardif *et al*, 2010; Chen *et al*, 2013; Guo *et al*, 2021). Serp‐1 protein used in this research is GMP‐compliant > 95% pure, as determined by Coomassie‐stained SDS–PAGE and reverse‐phase HPLC; endotoxin free by LAL (Limulus amebocyte lysate) assay. For PEGylation, Serp‐1 is incubated with mPEG‐NHS (5 K) (Nanocs Inc., #PG1‐SC‐5k‐1, NY) in PBS buffer (pH 7.8) at 4°C overnight according to standard PEGylation protocols (Guo *et al*, 2021). PEGSerp‐1 is purified by FPLC using an ÄKTA pure protein purification system with Superdex‐200. PEGSerp‐1 activity is analyzed by binding to active uPA and assessed on Western blot (Appendix Fig S1).

### Histological analysis

Tissues were analyzed by routine hematoxylin and eosin (H&E) staining as well as immunohistochemical (IHC) staining. Total inflammatory cell infiltrate area, hemorrhage when present, and consolidation areas were measured and normalized to the total lung area examined (Chen *et al*, 2013; Guo *et al*, 2021). Three high‐power fields were assessed for each specimen and the mean for positively stained cell counts per 40× field was calculated. IHC was performed for the detection of immune cell markers as well as uPAR and C5b‐9 MAC. iNOS and F4/80 for macrophage; CD3, CD4, and CD8 for T lymphocytes; and Ly6G for neutrophils. For IHC primary antibodies included C5b/9 antibody (Abcam, ab 55811, 1:200), uPAR (R&D Systems, AF534, 1:100), fibrinogen antibody (Abcam, ab34269, 1:200), factor X antibody (Abcam, ab196023, 1:200), and uPAR (R&D Systems, AF534, 1:100). Ly6G (Invitrogen, Carlsbad, CA, USA, #14‐5931‐82, 1:100), CD3 (Abcam, ab6590, 1:100), CD4 (Abcam, ab183685, 1:1,000), arginase‐1 (Cell Signaling, 93668, 1:200), iNOS (Abcam, ab15323, 1:100), and goat anti‐Rat IgG2a Hrp‐conjugated Cat: A110‐109P. HRP‐conjugated secondary antibodies against rabbit or goat IgG were applied at a dilution of 1:500 for 1 h at room temperature. HRP‐conjugated secondary antibody given alone without primary antibody was used as negative control for each stain. Antigens were revealed with ImmPACT DAB (Vector Labs, USA), counterstained with Gil's formula #3 hematoxylin, and mounted with Cytoseal XYL. Sections were examined using an Olympus BX51 microscope with 4×–100× objectives, a Prior ProScan II stage, and Olympus DP74 CMOS camera and cellSens software analysis system.

An independent pathology score was also performed blinded (SG), with score based on intra‐alveolar edema and chronic inflammatory cells (lymphocytes and macrophages). If the airspaces were clean and open without significant inflammation or edema, Score assigned was 1+. For airspaces partially open, with increased chronic inflammation with or without edema, score assigned is 2+. For sections with a majority of airspaces consolidated, with marked chronic inflammation with or without edema, score assigned is 3+.

### RNA isolation and qPCR

Samples were assayed for gene expression in infected lung and heart tissues, with and without PEGSerp‐1 treatment as well as in normal uninfected lung and heart isolates. Lung and heart samples from controls or SARS‐CoV‐2 MA30‐infected mice were collected 4 days post‐infection and frozen. Samples were also collected from formalin‐fixed, paraffin‐embedded (FFPE) lung and heart samples at 2 and 7 days follow‐up. For RNA extraction, tissues were harvested in RNA STAT‐60 (Tel‐Test) and disrupted by bead homogenization (Omni, INC). Isolated mouse lungs were transferred to Trizol (Thermo Fisher Scientific). RNA was extracted using the Qiagen RNAeasy Mini Kit (Qiagen, 74106), for frozen tissues and using the Qiagen RNAeasy kit (Qiagen 73504) for FFPE specimens, according to the manufacturer's instructions. Briefly, 0.2 ml chloroform was added to 1 ml of RNA STAT‐60, samples were mixed for 30 s, kept on ice for 5 min, and centrifuged 12,000 *g* for 15 min at 4°C. 0.5 ml of 100% ethanol was added to aqueous phase (RNA), samples were kept for 5 min at 4°C, and centrifuged as above. The final RNA pellet was resuspended in molecular biology‐grade water (Sigma). Total RNA was reverse transcribed with an oligo (dT) primer and a Moloney murine leukemia virus reverse transcriptase (MMLV RT, TakaraBio). cDNA was analyzed by quantitative PCR amplification using SYBR Green qPCR Master Mix (QuantaBio) on a Bio‐Rad CFX96 Real‐Time PCR Detection System. Primers were designed to amplify mRNA‐specific sequences, and analysis of the melt curve confirmed the amplification of single products. Uninfected samples were used as controls. Relative expression was normalized to GAPDH. Primer sequences used are provided in Appendix Table S2. Primers were designed using the IDT website, PrimerQuest Tool, for qPCR and confirmed in the literature.

Primers used for qPCR included SARSCoV‐2 E and N genes, Gapdh, Isg15, Fx, Tpa, Upa, Mmp2, Mmp‐9, Upar, Pai‐1, Nsp, Tnf, Il‐1, Il‐6, Il‐10, Tgfb, Vegf, C3, C5, and C1inh (Appendix Table S2). SARS‐Cov‐2 E and N gene primers were provided by Dr B Hogue and Gapdh standard by Dr M Rahman. Real‐time qPCR was analyzed on a BioradCFX96 Real Time PCR array with C1000 Touch Thermal cycler.

### Statistical analysis

Immunohistochemical analyses were read blinded to treatments and mouse strain. In the MA10 studies, the histology was evaluated by investigators blinded to treatments. In the MA30 studies, investigators infecting and treating the mice were blinded to the treatments, and tissue samples and histological analyses were also blinded. Significance in each parameter was assessed by StatView version 5.0.1 (SAS Institute, Inc.) or Graph Pad Prism using analysis of variance (ANOVA) with Fischer's LSD (least significant difference) comparison, or a Student's unpaired *t‐*test (*P* < 0.05 considered significant). Consolidation was measured as areas with inflammatory cell invasion and thrombosis causing loss of open spaces. The areas with greatest positive staining were used for IHC cell counts. The mean for three areas of consolidation and the mean for three positively stained cell counts in lung or heart microscopy sections at 20× or 40× high‐power field were calculated and used for statistical analysis. Kaplan–Meier estimates were used for survival analysis. Significance denoted in Figs 1, 2, 3, 4, 5, 6, 7 is represented as *P < 0.05, **P < 0.01, ***P < 0.001, and ****P < 0.0001.

## Author contributions

**Alexandra R Lucas:** Conceptualization; resources; data curation; formal analysis; supervision; funding acquisition; validation; methodology; writing – original draft; project administration. **Liqiang Zhang:** Conceptualization; data curation; formal analysis; supervision; validation; investigation; visualization; methodology; project administration; writing – review and editing. **Yize (Henry) Li:** Conceptualization; resources; data curation; supervision; funding acquisition; validation; investigation; methodology; project administration. **Karen Kibler:** Resources; data curation; supervision; investigation; visualization; methodology; project administration. **Simona Kraberger:** Resources; data curation; formal analysis; supervision; investigation; methodology. **Arvind Varsani:** Conceptualization; data curation; formal analysis; investigation; methodology. **Julie Turk:** Data curation; investigation; methodology. **Nora Elmadbouly:** Data curation; investigation; methodology. **Emily Aliskevich:** Data curation; investigation; methodology. **Laurel Spaccarelli:** Data curation; formal analysis; investigation; methodology. **Bereket Estifanos:** Data curation; formal analysis; investigation; methodology. **Junior Enow:** Data curation; investigation; methodology. **Isabela Rivabem Zanetti:** Data curation; formal analysis; investigation; methodology. **Nicholas Saldevar:** Data curation; investigation; methodology. **Efrem Lim:** Conceptualization; investigation; methodology. **Jessika Schlievert:** Data curation; investigation; methodology. **Kyle Browder:** Data curation; investigation; methodology. **Anjali Wilson:** Data curation; investigation; methodology. **Fernando Arcos Juan:** Data curation; investigation; methodology. **Aubrey Pinteric:** Data curation; investigation; methodology. **Aman Garg:** Data curation; investigation; methodology. **Savanah Gisriel:** Data curation; formal analysis; investigation. **Henna Monder:** Investigation; methodology. **Bertram Jacobs:** Conceptualization; funding acquisition; investigation; methodology. **Rohan Saju:** Investigation; methodology. **Timothy L Karr:** Conceptualization; data curation; formal analysis; investigation; methodology. **Esther Borges Florsheim:** Conceptualization; data curation; investigation; methodology. **Vivek Kumar:** Conceptualization; investigation. **John Wallen:** Conceptualization; investigation. **Masmudur Rahman:** Conceptualization; data curation; funding acquisition; investigation; methodology. **Grant McFadden:** Conceptualization; data curation; investigation; methodology; writing – review and editing. **Brenda G Hogue:** Conceptualization; resources; formal analysis; supervision; funding acquisition; investigation; methodology; project administration; writing – review and editing.

## Disclosure and competing interests statement

Serpass Biologics, Inc.—Serpass Biologics is a new start‐up company at ASU, Biodesign Institute, and has provided no funding toward this research or to Dr Lucas and Dr Zhang. Drs Lucas, Zhang, and Wallen are co‐inventors.

## For more information

For additional references on the serpin class of regulatory proteins and more specifically on virus‐derived immune‐modulating proteins, we would refer to the following references.

For viral infections, ARDS, specifically SARS‐CoV‐2, and immune and coagulation‐associated abnormalities:

Acanfora D, *et al* (2021) *Viruses* **13**, 1904. doi: 10.3390/v13101904
Arnold DT, *et al* (2021) *Emerg Med J* **38:** 543–548. doi: 10.1136/emermed‐2020‐210380

For serpins as immune‐modulating proteins:

Bouton MC, *et al* (2023) *EMBO Mol Med* e17144. DOI 10.15252/emmm.202217144

For virus derived immune and coagulation modulating proteins:

Chen H, *et al* (2013) *Antimicrob Agents Chemother* **57**: 4114–4127
Yaron JR, *et al* (2021) *Front Cardiovasc Med* **8**, 648947. doi: 10.3389/fcvm.2021.648947

## Supporting information



Appendix S1

Source Data for Figure 2

Source Data for Figure 3

Source Data for Figure 4

Source Data for Figure 5

Source Data for Figure 6

Source Data for Figure 7

Source Data for Figure 8

Source Data for Figure 9

## Data Availability

This study includes no data deposited in external repositories.
