# Supplementary material for: Viral anti‐inflammatory serpin reduces immuno‐coagulopathic pathology in SARS‐CoV‐2 mouse models of infection
Source: EMBO Mol Med. 2023 Aug 3;15(9):e17376. doi: 10.15252/emmm.202317376 (PMC10493584; doi:10.15252/emmm.202317376)
Supplement: Supplementary file 1 — Appendix S1 [file EMMM-15-e17376-s004.pdf]

Appendix – Supplementary Material

Table of Contents

Appendix Figure S1

Appendix Figure S2

Appendix Table S1

Appendix Table S2

Figure Legends

Appendix Figure S1 - Western blot illustrating PEGSerp-1 binding to His labelled urokinase type plasminogen activator (uPA).

Appendix Figure S2 - Body weight measured after SARS-CoV-2 MA30 infection – Four day follow up with prophylactic PEGSerp-1 treatments (C57BL/6 mouse model). A. mean body weights for SARS MA30 infected mice with and without PEGSerp-1 treatment demonstrates significantly improved weight gain on days 4 and 4 with PEGSerp-1 treatment (Red) given daily when compared to saline controls (Black).
B. Individual weights measured for each mouse over the 4 day follow up. N = 19 mice. Student's T test - * $p < 0.05$, ** $p < 0.01$

Appendix Table S1 - Immunoprecipitation of PEGserp-1 bound molecules as measured by mass spectrometry demonstrating pull down of thrombolytic and complement proteases. Immunoprecipitation of PEGSerp-1 bound proteins form a lupuds lung hemorrhage model (Guo 2021).

Appendix Table S2 - Primer sequences used for qPCR analyses of SARS-CoV-2 infected lung and heart extracts.

Appendix Figure S1

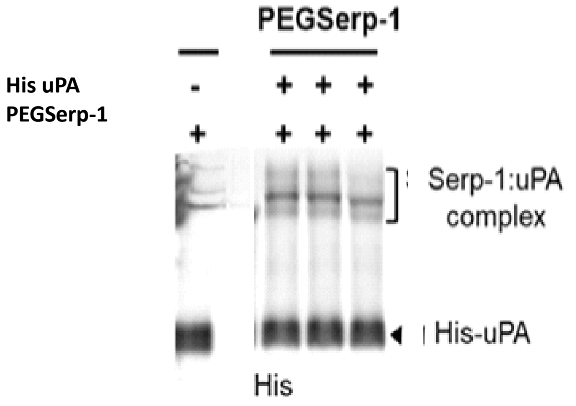

Appendix Figure S2. Prophylactic PEGSerp-1 treatments in SARS-CoV-2 MA30 Day 4 follow up

A. Mean body weight

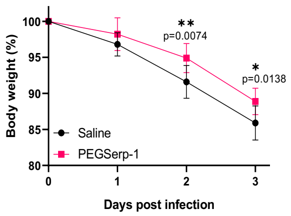

B. Individual Mean body weights

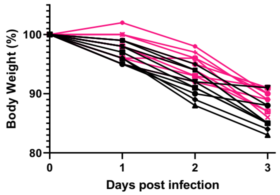

Appendix Table S1- MS analysis DAH PEG Serp-1 Immunopresipitation (IP)

Accession	Description	Symbol	# Peptides
P01027	Complement C3	C3 PE	34
B7FAV1	Filamin, alpha	Flna PE	23
P60710	Actin, cytoplasmic 1	Actb PE	17
E9PV24	Fibrinogen alpha chain	Fga PE	12
P20918	Plasminogen	Plg PE	11
P01029	Complement C4-B	C4b PE	9
P98086	Complement C1q subunit A	C1qa PE	4
P04186	Complement factor B	Cfb PE	4
Q8CG14	Complement C1s-A	C1sa PE	4
P14106	Complement C1q subunit B	C1qb PE	3
Q8CG16	Complement C1r-A	C1ra PE	3
Q02105	Complement C1q subunit C	C1qc PE	2

*Average Xcorr >4.0

Appendix Table S2 - Primer Sequences for qPCR

Gene	Primers
**MCP-1F**	CTCACCTGCTGCTACTCATTC
**MCP-1R**	ACTACAGCTTCTTTGGGACAC
**Fxa-F**	GGAACACAGAGAAGGAAGAAGG
**Fxa-R**	CTCAGCACGGCGATATCATAG
**GAPDH-F**	CATCACTGCCACCCAGAAGACTG
**GAPDH-R**	ATGCCAGTGAGCTTCCCGTTCAG
**GzmB-F**	GGCGCAATGTCAATGTGAAG
**GzmB-R**	CTGTCAGCTCAACCTCTTGTAG
**IL-10F**	ACAGCCGGGAAGACAATAAC
**IL-10R**	CAGCTGGTCCTTTGTTTGAAAG
**IL-1bF**	GCAACTGTTCCTGAACTCAACT
**IL-1bR**	ATCTTTTGGGGTCCGTCAACT
**IL-6F**	GATAAGCTGGAGTCACAGAAGG
**IL-6R**	TTGCCGAGTAGATCTCAAAGTG
**MMP2F**	GTTCAACGGTCGGGAATACA
**MMP2R**	GCCATACTTGCCATCCTTCT
**MMP9F**	TGCACTGGGCTTAGATCATTC
**MMP9R**	TGCCGTCTATGTCGTCTTTATTC
**Neuroserpin-F**	TTCCTCTCCAAAGCTGTTCAC
**Neuroserpin-R**	CAGCACAGCCATCCTACTAATC
**PAI-1F**	CTCCACAGCCTTTGTCATCT
**PAI-1R**	ATTGTCTCTGTCGGGTTGTG
**PAR2/F2rl1-F**	TTAGAAACCCAGCCTCCAATC
**PAR2/F2rl1-R**	AATGTAGACGACCGGAAGAAAG
**TGFb1-F**	GGTGGTATACTGAGACACCTTG
**TGFb1-R**	CCCAAGGAAAGGTAGGTGATAG
**TNF-F**	TTGTCTACTCCCAGGTTCTCT
**TNF-R**	GAGGTTGACTTTCTCCTGGTATG
**TPA-F**	GGGAATACATGGGAGGTTCAG
**TPA-R**	GACTGGTGCTGTTGGTAAGT
**UPA-F**	CGCACACTGCTTCATTCAAC
**UPA-R**	GCTCCACCTCAAACTTCATCT
**UPAR-F**	GAGCTTGAAGGATGAGGACTAC
**UPAR-R**	TTCAGGAAGTGAAAGGTCTGG
**VEGF-F**	CACTTCCAGAAACACGACAAAC
**VEGF-R**	TGGAACCGGCATCTTTATCTC
**C1INH-F**	GCTGAGAACACCAACCATAAGA
**C1INH-R**	CCACTTGGCACTCAAGTAGAC
