# Supplementary figures and images for: Viral anti‐inflammatory serpin reduces immuno‐coagulopathic pathology in SARS‐CoV‐2 mouse models of infection
Source: EMBO Mol Med. 2023 Aug 3;15(9):e17376. doi: 10.15252/emmm.202317376 (PMC10493584; doi:10.15252/emmm.202317376)

## Slide 1
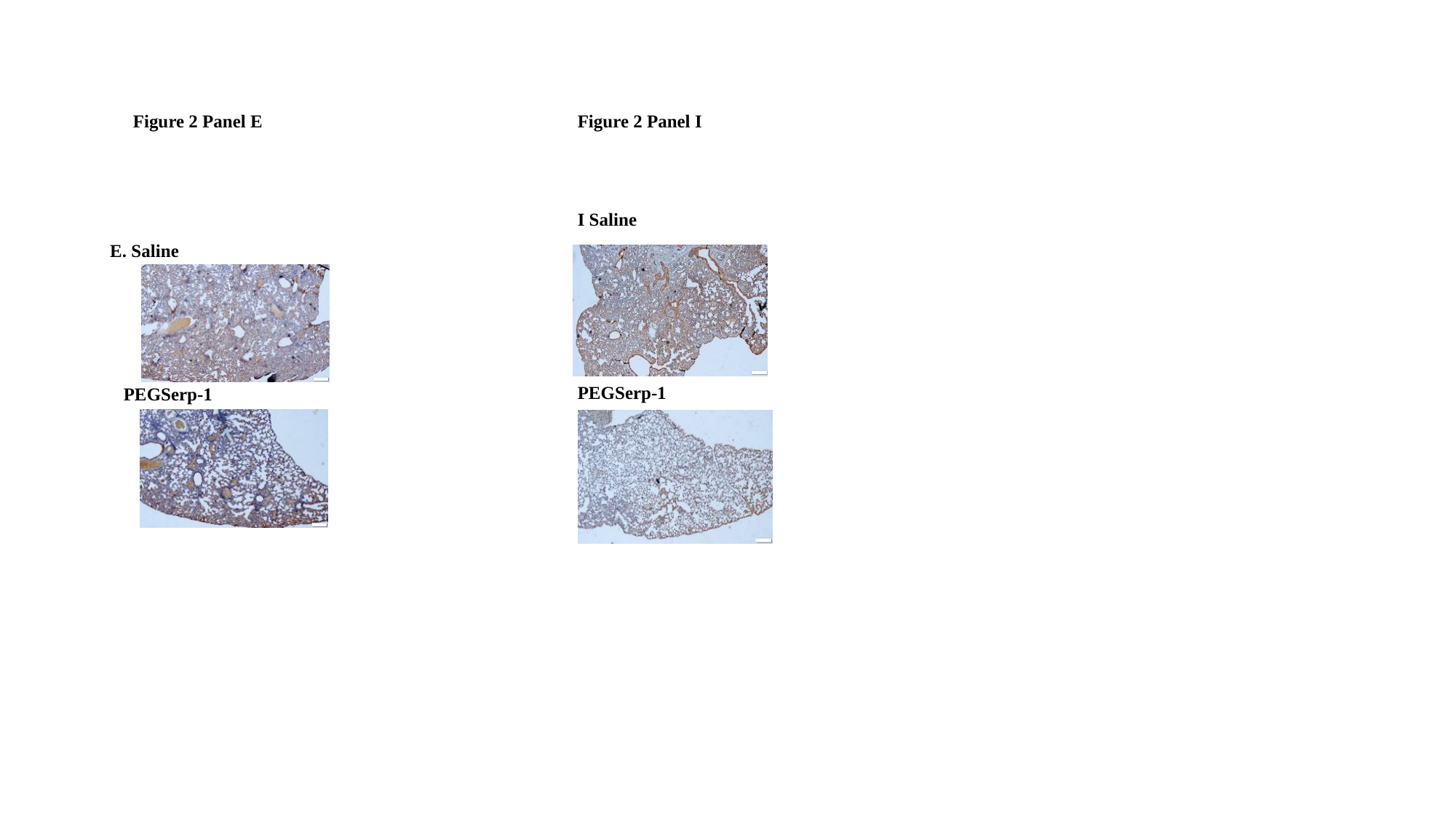

Figure 2 Panel I
Figure 2 Panel E
I Saline
E. Saline
PEGSerp-1
 PEGSerp-1

Supplement: Supplementary file 2 — Source Data for Figure 2 [file EMMM-15-e17376-s005.zip › Figure 2 Source data/Figure 2 Panels E and I Micrograph Source data.pptx]

## Slide 1
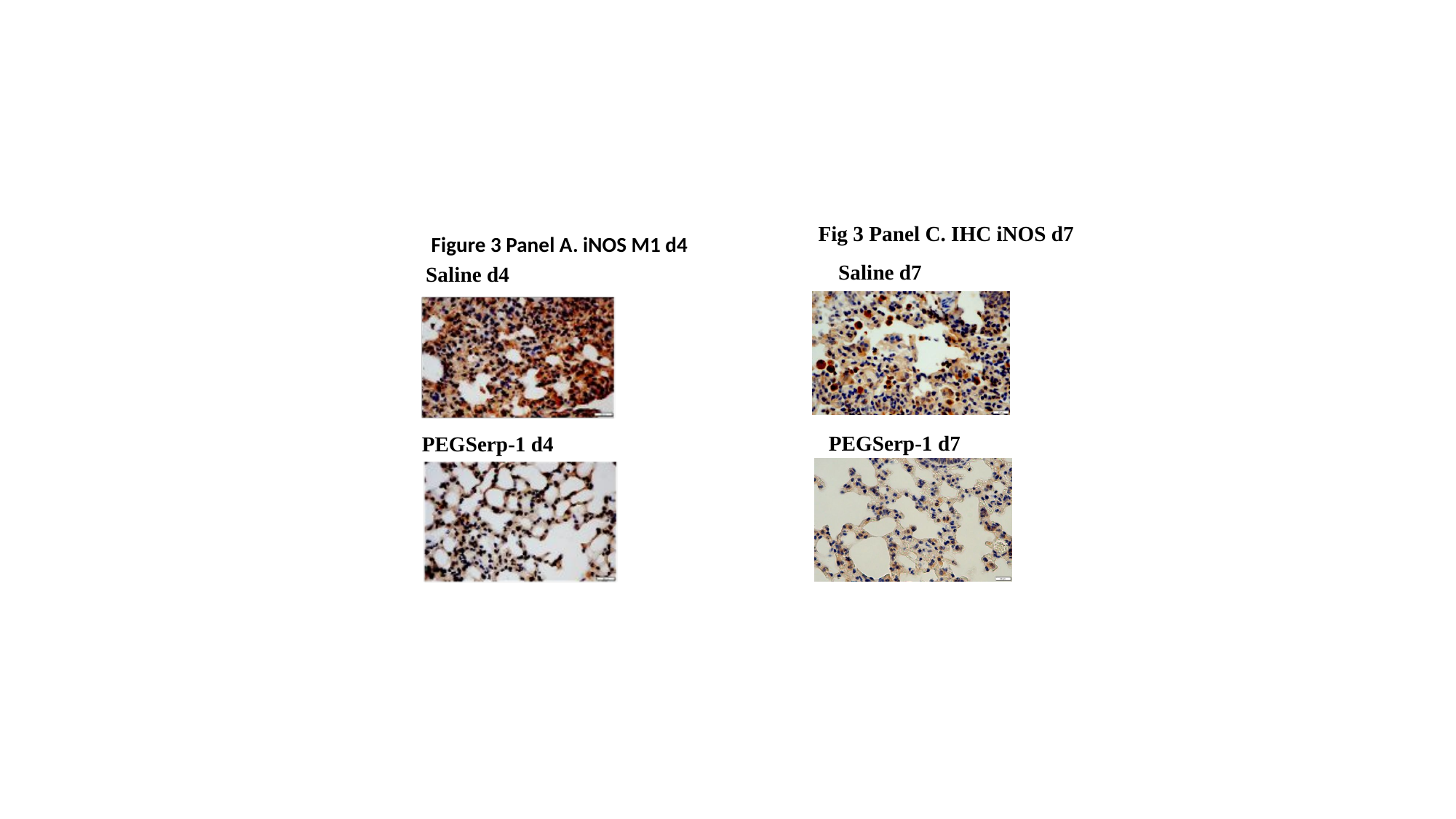

Fig 3 Panel C. IHC iNOS d7
Figure 3 Panel A. iNOS M1 d4
Saline d7
Saline d4
PEGSerp-1 d7
PEGSerp-1 d4

Supplement: Supplementary file 3 — Source Data for Figure 3 [file EMMM-15-e17376-s001.zip › Figure 3 source data/Figure 3 Panels A and C micrograph source data.pptx]

## Slide 1
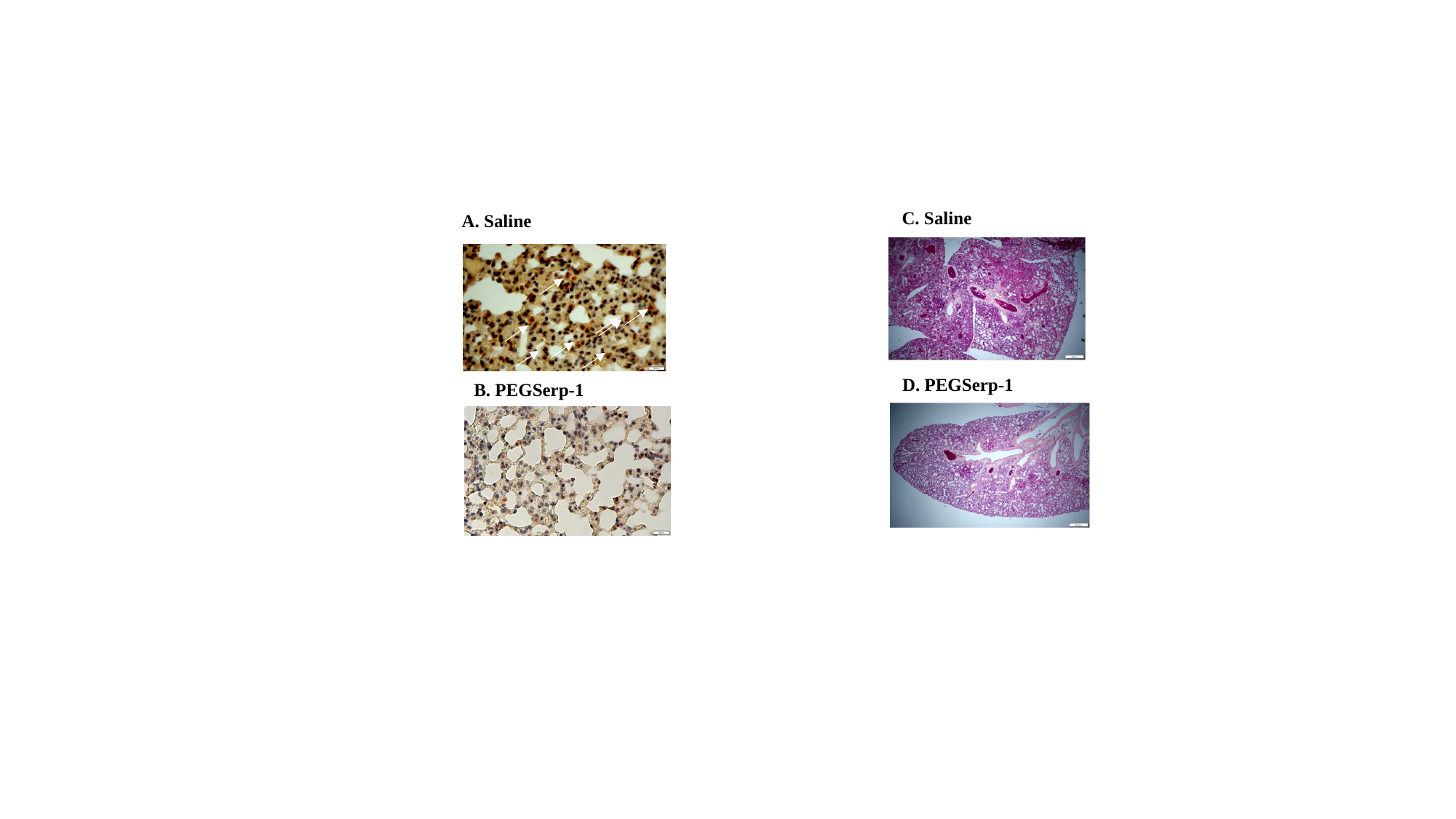

C. Saline
A. Saline
D. PEGSerp-1
B. PEGSerp-1

Supplement: Supplementary file 4 — Source Data for Figure 4 [file EMMM-15-e17376-s003.zip › Figure 4 Source data/Figure 4 Panels A,B,C,D micrograph source data.pptx]

## Slide 1
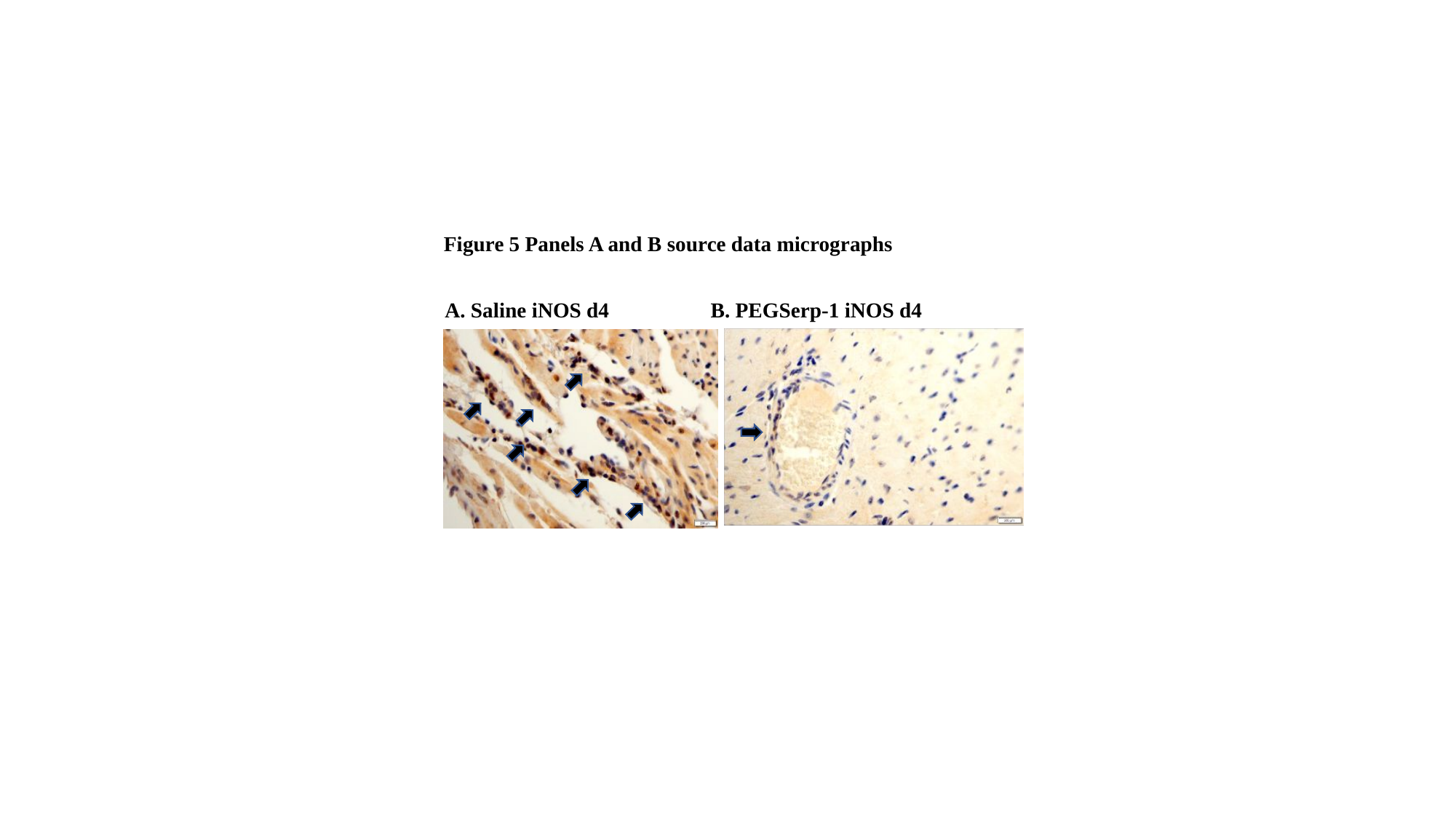

Figure 5 Panels A and B source data micrographs
A. Saline iNOS d4	 B. PEGSerp-1 iNOS d4

Supplement: Supplementary file 5 — Source Data for Figure 5 [file EMMM-15-e17376-s006.zip › Figure 5 source data/Figure 5 Panels A and B source data micrographs.pptx]

## Slide 1
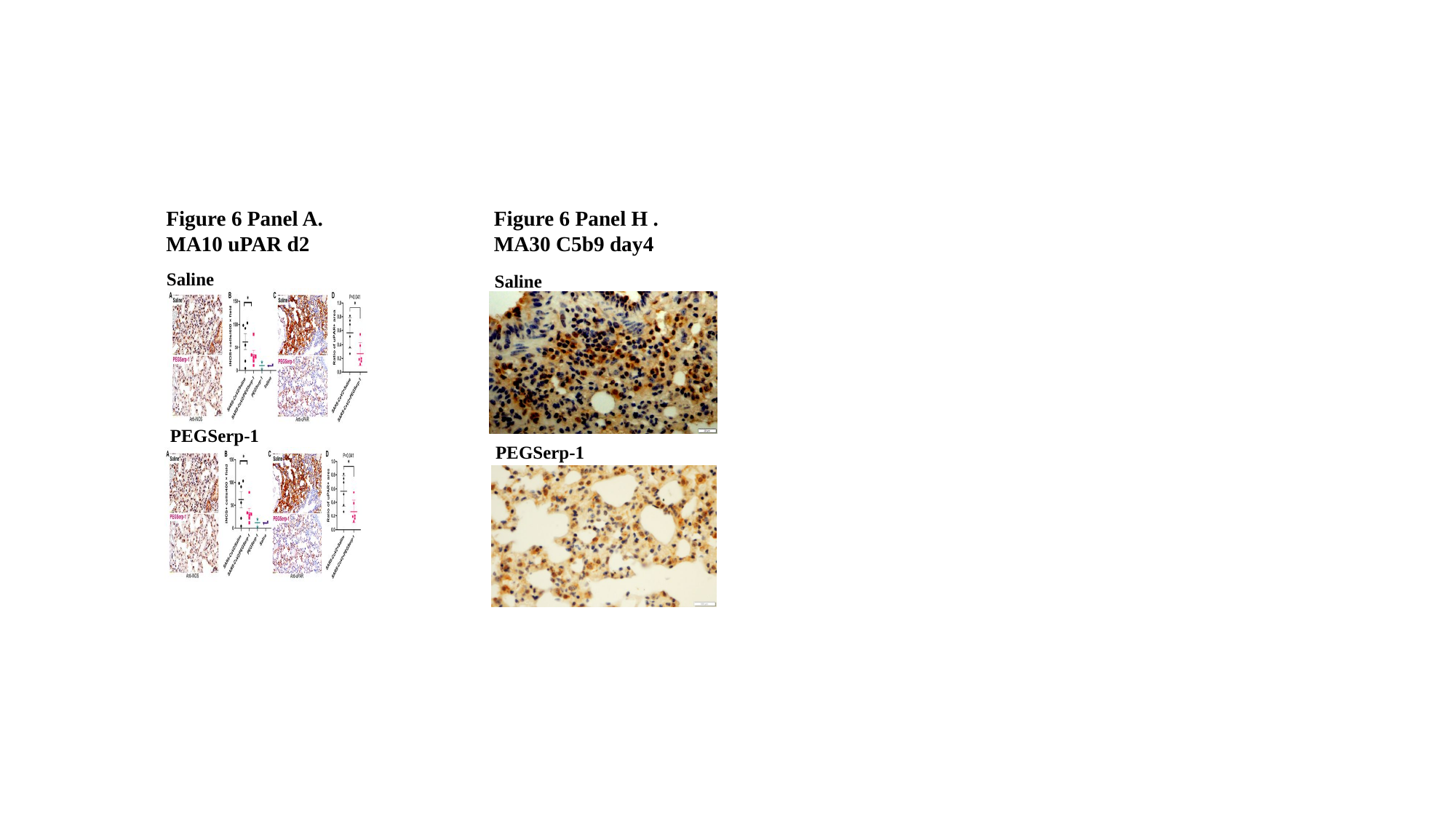

Figure 6 Panel A. MA10 uPAR d2
Figure 6 Panel H . MA30 C5b9 day4
Saline
Saline
PEGSerp-1
PEGSerp-1

Supplement: Supplementary file 6 — Source Data for Figure 6 [file EMMM-15-e17376-s010.zip › Figure 6 Source data/Figure 6 Panels A and H Source data Micrographs .pptx]

## Slide 1
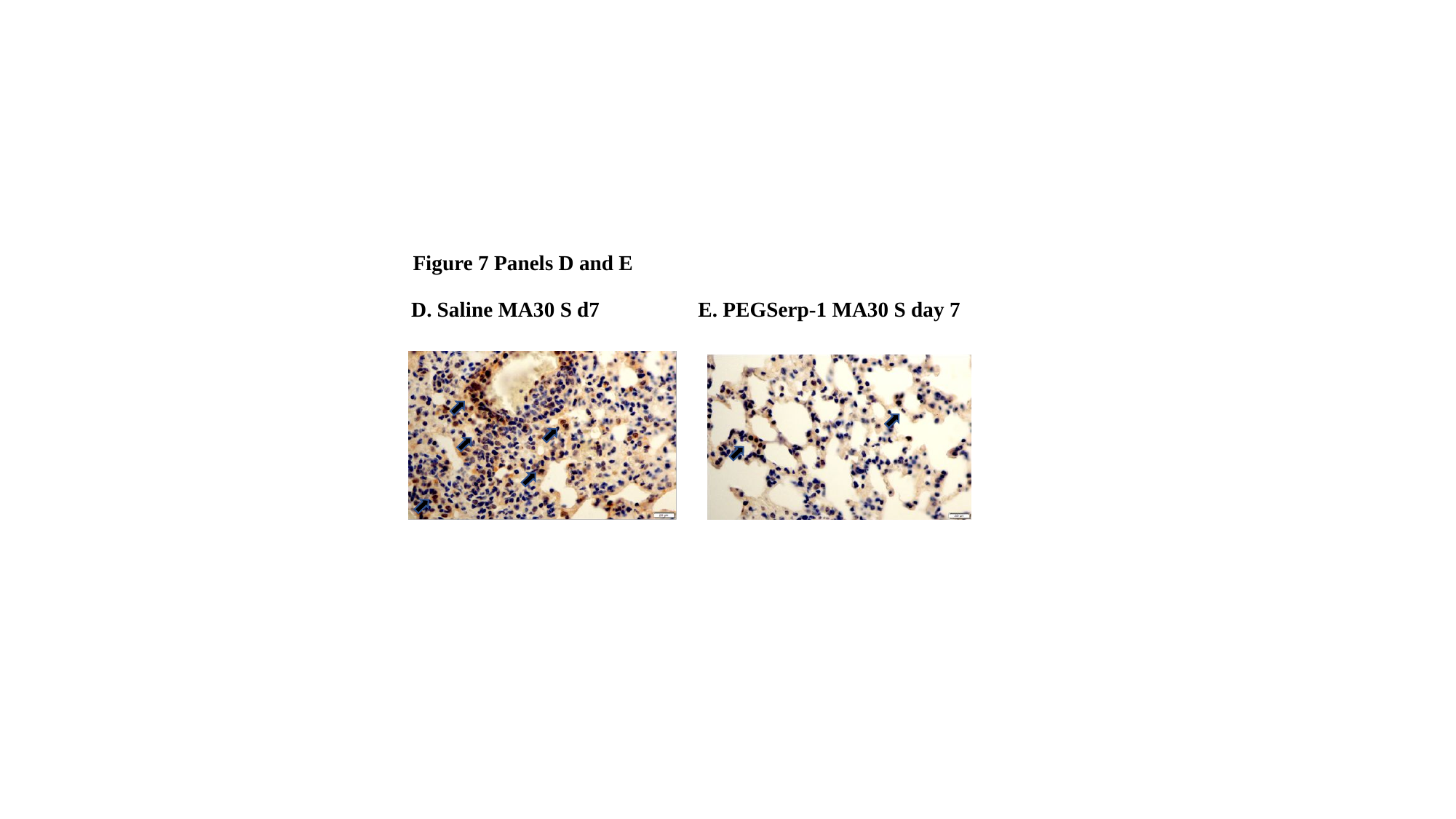

Figure 7 Panels D and E
D. Saline MA30 S d7 	 E. PEGSerp-1 MA30 S day 7

Supplement: Supplementary file 7 — Source Data for Figure 7 [file EMMM-15-e17376-s008.zip › Figure 7 Source data/Figure 7 Panels D and E source data micrographs.pptx]
