# Supplementary material for: Viral anti‐inflammatory serpin reduces immuno‐coagulopathic pathology in SARS‐CoV‐2 mouse models of infection
Source: EMBO Mol Med. 2023 Aug 3;15(9):e17376. doi: 10.15252/emmm.202317376 (PMC10493584; doi:10.15252/emmm.202317376)
Supplement: Supplementary file 10 [file EMMM-15-e17376-s009.pdf]

EMBO Molecular Medicine

Viral anti-inflammatory serpin reduces immuno-coagulopathic pathology in SARS-CoV-2 infection

Alexandra Lucas, Liqiang Zhang, Yize Li, Karen Kibler, Simona Kraberger, Arvind Varsani, Julie Turk, Nora Elmadbouly, Emily Aliskevich, Laurel Spaccarelli, Bereket Estifanos, Junior Enow, Isabela Zanetti, Nicholas Saldevar, Efrem Lim, Jessika Schlievert, Kyle Browder, Anjali Wilson, Fernando Juan, Aubrey Pinteric, Aman Garg, Savanah Gisriel, Henna Monder, Bertram Jacobs, Rohan Saju, Timothy Karr, Esther Florsheim, Vivek Kumar, John Wallen, Masmudur Rahman, Grant McFadden, and Brenda Hogue
DOI: 10.15252/emmm.202317376

Corresponding authors: Alexandra Lucas (alexluc1@asu.edu) , Brenda Hogue (Brenda.Hogue@asu.edu)

Review Timeline:

Submission Date:	2nd Jan 23
Editorial Decision:	1st Feb 23
Revision Received:	13th Jun 23
Editorial Decision:	3rd Jul 23
Revision Received:	13th Jul 23
Accepted:	14th Jul 23

Editor: Zeljko Durdevic

Transaction Report:

(Note: With the exception of the correction of typographical or spelling errors that could be a source of ambiguity, letters and reports are not edited. Depending on transfer agreements, referee reports obtained elsewhere may or may not be included in this compilation. Referee reports are anonymous unless the Referee chooses to sign their reports.)

1st Feb 2023

Dear Prof. Lucas,

Thank you for the submission of your manuscript to EMBO Molecular Medicine. We have now received feedback from the three reviewers who agreed to evaluate your manuscript. As you will see from the reports below, referees recognize potential interest of the study, but also raise serious and partially overlapping concerns that should be addressed in a major revision. Particular attention should be given to careful rewriting and restructuring of the manuscript to enhance its readability and clarity. Additional expiation and rational for the dosing used in the study should be provided. If you would like to discuss further the points raised by the referees, I am available to do so via email or video. Let me know if you are interested in this option.

Further consideration of a revision that addresses reviewers' concerns in full will entail a second round of review. EMBO Molecular Medicine encourages a single round of revision only and therefore, acceptance or rejection of the manuscript will depend on the completeness of your responses included in the next, final version of the manuscript. For this reason, and to save you from any frustrations in the end, I would strongly advise against returning an incomplete revision.

We would welcome the submission of a revised version within three months for further consideration. Please let us know if you require longer to complete the revision.

I look forward to receiving your revised manuscript.

Yours sincerely,

Zeljko Durdevic

Zeljko Durdevic
Editor
EMBO Molecular Medicine

*****

When submitting your revised manuscript, please carefully review the instructions that follow below. We perform an initial quality control of all revised manuscripts before re-review; failure to include requested items will delay the evaluation of your revision.

We require:

1) A .docx formatted version of the manuscript text (including legends for main figures, EV figures and tables). Please make sure that the changes are highlighted to be clearly visible.

2) Individual production quality figure files as .eps, .tif, .jpg (one file per figure). For guidance, download the 'Figure Guide PDF': (https://www.embopress.org/page/journal/17574684/authorguide#figureformat).

3) A .docx formatted letter INCLUDING the reviewers' reports and your detailed point-by-point responses to their comments. As part of the EMBO Press transparent editorial process, the point-by-point response is part of the Review Process File (RPF), which will be published alongside your paper.

4) A complete author checklist, which you can download from our author guidelines (https://www.embopress.org/page/journal/17574684/authorguide#submissionofrevisions). Please insert information in the checklist that is also reflected in the manuscript. The completed author checklist will also be part of the RPF.

5) Please note that all corresponding authors are required to supply an ORCID ID for their name upon submission of a revised manuscript.

6) It is mandatory to include a 'Data Availability' section after the Materials and Methods. Before submitting your revision, primary datasets produced in this study need to be deposited in an appropriate public database, and the accession numbers and database listed under 'Data Availability'. Please remember to provide a reviewer password if the datasets are not yet public (see https://www.embopress.org/page/journal/17574684/authorguide#dataavailability).

In case you have no data that requires deposition in a public database, please state so in this section. Note that the Data Availability Section is restricted to new primary data that are part of this study.

7) For data quantification: please specify the name of the statistical test used to generate error bars and P values, the number (n) of independent experiments (specify technical or biological replicates) underlying each data point and the test used to calculate p-values in each figure legend. The figure legends should contain a basic description of n, P and the test applied. Graphs must include a description of the bars and the error bars (s.d., s.e.m.). See also 'Figure Legend' guidelines: https://www.embopress.org/page/journal/17574684/authorguide#figureformat

8) At EMBO Press we ask authors to provide source data for the main manuscript figures. Our source data coordinator will contact you to discuss which figure panels we would need source data for and will also provide you with helpful tips on how to upload and organize the files.

9) Our journal encourages inclusion of *data citations in the reference list* to directly cite datasets that were re-used and obtained from public databases. Data citations in the article text are distinct from normal bibliographical citations and should directly link to the database records from which the data can be accessed. In the main text, data citations are formatted as follows: "Data ref: Smith et al, 2001" or "Data ref: NCBI Sequence Read Archive PRJNA342805, 2017". In the Reference list, data citations must be labeled with "[DATASET]". A data reference must provide the database name, accession number/identifiers and a resolvable link to the landing page from which the data can be accessed at the end of the reference. Further instructions are available at .

10) We replaced Supplementary Information with Expanded View (EV) Figures and Tables that are collapsible/expandable online. A maximum of 5 EV Figures can be typeset. EV Figures should be cited as 'Figure EV1, Figure EV2" etc... in the text and their respective legends should be included in the main text after the legends of regular figures.

- For the figures that you do NOT wish to display as Expanded View figures, they should be bundled together with their legends in a single PDF file called *Appendix*, which should start with a short Table of Content. Appendix figures should be referred to in the main text as: "Appendix Figure S1, Appendix Figure S2" etc.

- Additional Tables/Datasets should be labeled and referred to as Table EV1, Dataset EV1, etc. Legends have to be provided in a separate tab in case of .xls files. Alternatively, the legend can be supplied as a separate text file (README) and zipped together with the Table/Dataset file.

See detailed instructions here:

.

11) The paper explained: EMBO Molecular Medicine articles are accompanied by a summary of the articles to emphasize the major findings in the paper and their medical implications for the non-specialist reader. Please provide a draft summary of your article highlighting

- the medical issue you are addressing,

- the results obtained and

- their clinical impact.

This may be edited to ensure that readers understand the significance and context of the research. Please refer to any of our published articles for an example.

12) For more information: There is space at the end of each article to list relevant web links for further consultation by our readers. Could you identify some relevant ones and provide such information as well? Some examples are patient associations, relevant databases, OMIM/proteins/genes links, author's websites, etc...

13) Author contributions: You will be asked to provide CRediT (Contributor Role Taxonomy) terms in the submission system. These replace a narrative author contribution section in the manuscript.

14) A Conflict of Interest statement should be provided in the main text.

15) Every published paper now includes a 'Synopsis' to further enhance discoverability. Synopses are displayed on the journal webpage and are freely accessible to all readers. They include a short stand first (maximum of 300 characters, including space) as well as 2-5 one-sentences bullet points that summarizes the paper. Please write the bullet points to summarize the key NEW findings. They should be designed to be complementary to the abstract - i.e. not repeat the same text. We encourage inclusion of key acronyms and quantitative information (maximum of 30 words / bullet point). Please use the passive voice. Please attach these in a separate file or send them by email, we will incorporate them accordingly.

Please also suggest a striking image or visual abstract to illustrate your article as a PNG file 550 px wide x 300-600 px high.

EMBO Molecular Medicine has a "scooping protection" policy, whereby similar findings that are published by others during review or revision are not a criterion for rejection. Should you decide to submit a revised version, I do ask that you get in touch after three months if you have not completed it, to update us on the status.

Please note: When submitting your revision you will be prompted to enter your funding and payment information. This will allow Wiley to send you a quote for the article processing charge (APC) in case of acceptance. This quote takes into account any reduction or fee waivers that you may be eligible for. Authors do not need to pay any fees before their manuscript is accepted and transferred to the publisher.

EMBO Press participates in many Publish and Read agreements that allow authors to publish Open Access with reduced/no publication charges. Check your eligibility: https://authorservices.wiley.com/author-resources/Journal-Authors/open-access/affiliation-policies-payments/index.html

***** Reviewer's comments *****

Referee #1 (Remarks for Author):

The manuscript by Zhang and Li, et al, examines the effect of PEGSerp-1 on disease outcome and coagulation factors in two mouse-adapted SARS-CoV-2 models. The topic is of interest to the SARS-CoV-2 treatment field, as coagulopathies are known to significantly contribute to mortality in ARDS patients. However, the manuscript is hampered by including too much background information and is very challenging to read as currently written. The following are suggestions to improve the readability and interpretation of the information presented.

Major Points:
1. Overall, this manuscript contains too much detail and is very challenging to read and understand what all was performed as currently written. The manuscript includes several compound sentences that are too long and would benefit from being broken up into several shorter sentences. Additionally, the authors should carefully read through the manuscript and correct typographical errors. The Introduction is overly long and includes more detail than necessary, particularly regarding the different coagulation cascade proteins. Additionally, much of this information appears to be repeated in the Results sections. Reducing the length of the Introduction by half, focusing on only the information directly relevant to the presented research, would be much easier for the reader.

2, Was the effect of PEGSerp-1 treatment examined after infection with SARS-CoV-2 (i.e. treatment started upon commencement of clinical signs, 2 DPI)? This would be important in discerning whether PEGSerp-1 represent a potential treatment option for SARS-CoV-2 patients rather than just a proof-of-principle.

3. This reviewer finds it very surprising that the investigators did not observe any weight loss or clinical signs in the MA10-infected BALB/c mice, especially at such a high infection dose. Furthermore, the way the manuscript is written, it is confusing whether the data presented belongs to the MA10 or the MA30 model in each figure. The MA30 data appears to be more informative and more thoroughly examined of the two models. Therefore, this reviewer recommends the investigators consider moving the MA10 data to supplemental figures to help streamline the manuscript.

4. The inclusion of the Fig 9 data does not fit with the rest of the manuscript. Either the additional identified mammalian serpins should be examined in the MA30 model, or this data should be removed from the paper.

Minor Points:
1. It is unclear from the manuscript text when treatment with PEGSerp-1 began. An experimental design schematic at the beginning of Figs 1 and 3 would help illustrate the study setup and make it easier for the reader to understand what was done.

2. Most of the histopathological images need to be white-balanced throughout the manuscript.

3. The colors and shapes of the data points on the graphs should be standardized across the manuscript.

4. Fig 1: The clinical score graphs should be presented as a stacked bar graph for each treatment group so that the reader may see the full range of scores at each day post infection.

5. Fig 1: Presenting the 4 and 7 DPI body weights separately is not necessary since the results are the same. I recommend only the combined 4 and 7 DPI body weight be presented in the figure, with the individual animal weight and clinical score data moved to a supplemental figure.

6. Figs 1, 2, 4, 5, 6, 7, 8: Because the specific p values are described in the text, the figures would look less busy if only stars were included to denote significance as opposed to the specific p values. Please remove the specific p values from the figure legends as well, and include a general description of what each star represents (e.g. *p<0.05, **p<0.01, etc).

7. Figs 2, 4, and 5: The current presentation of the data between d4 and d7 is confusing, especially because the y-axes are not consistent. For parameters for which both d4 and d7 data are presented, both timepoints should be presented on the same graph, with a 2-way ANOVA and multiple comparisons post-test performed for statistical analysis. This will allow the reader to critically examine the effect of PEGSerp-1 treatment over time.

8. Figs 7 and 8: The gene expression data for the mock-treated and PEGSerp-1-treated mice should be normalized to the uninfected control mice (i.e. delta-delta CT method) rather than presenting the uninfected control mice data.

9. The Discussion section should include commentary on how well the MA10 and MA30 mouse models replicate coagulopathy features seen with severe SARS-CoV-2 infection.

10. To what BALB/c and C57BL/6 substrains did the mice used in the study belong? This is important, as different substrains show different levels of susceptibility to MA10 and MA30 infection.

Referee #2 (Comments on Novelty/Model System for Author):

This study is an animal model study and therefore I assessed medical impact as "N/A", using a narrow definition. The model is appropriate and there are no ethical issues.

Referee #2 (Remarks for Author):

In this study, Zhang and co-workers examine the effects of administering pegylated Serp-1 (PEGSerp-1) to mice infected with adapted strains of SARS-CoV-2. Serp-1 is a member of the serpin superfamily of protease inhibitors, a viral serpin encoded by the rabbit myxomatosis virus. Serp-1 has been demonstrated to have strong anti-inflammatory activity in a number of animal models of disease, an activity mediated by its interactions with urinary-type plasminogen activator (uPA) and its cellular receptor (uPAR) and possibly by its inhibition of multiple serine proteases regulating coagulation and fibrinolysis. Zhang and co-workers examined PEGSerp-1 effects when administered intraperitoneally in SARS-CoV-2-infected mice. They found that PEGSerp-1 treatment significantly improved wellness scores and weight in infected mice versus vehicle (saline) controls. These pathophysiological changes correlated with reduced iNOS expression in lung and heart, reduced c5b-9+ indicators of complement activity, and reduced markers of M1 inflammatory macrophages in SERP-1 treated mice.

Major points

1. This is an interesting and timely study congruent with the emerging picture of thrombo-inflammatory damage in severe SARS-CoV-2 infection. It is a substantial study suited for publication as a full paper and not a short report. The investigators are to be complimented on their diligence in using two strains of mice and two SARS-CoV-2 mouse-adapted models. Their data with respect to attenuation of weight loss and reduction in inducible nitric oxide synthase (iNOS) associated with PEGSerp-1 treatment is also clear, and some of their proposed mechanisms are reasonable given these data. In other cases, they looked for effects on many parameters and did not find them. These data are also important and add to the thoroughness of the report. However, the investigators unnecessarily complicate their findings by pointing out trends in the data. It would be simpler and more appropriate to class any finding with p > 0.05 as being not statistically significant. For one thing, there is no accepted definition of "trend" of which this reviewer is aware and Zhang et al apply it to data sets with p = 0.08 and even p = 0.20. Zhang et al have found many statistically significant results and do not need to address those that fail to reach statistical significance.
2. It was not clear to this reviewer if the "clinical score" used to assess the infected mice had been previously employed or if it was developed for this study. This point should be made more clearly. In either case, how has it been validated?
3. In the gene expression (qPCR) data, the investigators found a significant increase in uPAR and C1INH mRNA levels after PEGSerp-1 treatment in lung tissue and PAI-1 and C1INH levels in heart. However, while statistically significant, the increase in mean levels is on the order of 10%. How do they see this small change in mRNA levels being mechanistically linked to

PEGSerp1 action and the observed amelioration of inflammation in the infected mice? For secreted proteins like C1INH and PAI-1 are these tissues a significant site of synthesis? In addition, the investigators use y axis scales that differ for each parameter in Figures 7 and 8 and do not start at zero; they should at least alert readers to this fact by inserting a note into the relevant figure legends.
4. Most of the experimental design in the study is logical and clear. However, it is unclear to this reviewer why the results from a lupus diffuse alveolar hemorrhage model are presented at all (Figure 9) and these do not seem related to the current study.

Minor points

1. In Figure 7G the investigators perform an ANOVA comparing three data sets, one of which has n=1. Do they think this is appropriate? Even if some statisticians think it is mathematically defensible, is it best practice?
2. In the Discussion, there is a paragraph beginning "In this study..." and concluding with a citation of Tardif 2010 that is duplicated elsewhere in the paper and should be deleted.
3. "Antithrombin III" is an outdated term for antithrombin (Serpin C1) and should be replaced or expanded.
4. Why are only 6 days of data shown in some figures when the investigators followed mice for either 4 or 7 days?
5. What is lung consolidation? It would be helpful to a general readership to define this parameter.
6. At the beginning of the Results section "YL, BH" appears in parentheses but it is unclear what this means - perhaps a flag to two investigators on the team to comment on the section during its writing? If so this should be removed.
7. Is it appropriate to use a Student's unpaired t test if the data are not normally distributed? Wouldn't a Mann-Whitney or other non-parametric test be more appropriate?
8. In the first paragraph of the Introduction "can induce excess and damaging immune responses" should be "can induce excessive and damaging immune responses".

Referee #3 (Comments on Novelty/Model System for Author):

In this manuscript the authors present the effect of PEGylated myxoma virus-derived Serp-1 as a potential pharmaceutical agent for reduction of inflammatory and coagulation activity in SARS-CoV2 infection. While the subject of this manuscript is interesting and potentially relevant, there are several experimental shortcomings, e.g. regarding the dose of the agent (and the lack of proper dose-response experiments), the choice of saline as a control substance, the lack of any investigation in anti-coagulopathic effects although these are claimed by the authors, and some methodological issues. I think the authors should at least add experiments to address these issues.

Referee #3 (Remarks for Author):

The authors have investigated myxoma virus-derived Serp-1 as a potential pharmaceutical agent for reduction of inflammatory and coagulation activity in SARS-CoV2 infection. The following comments may be made:

1. I would suggest adding to the title of the manuscript that this was a study in mice.

2. Abstract ",,,as a self-defense strategy to combat clearance" is not entirely understandable. Why would an antifibrinolytic protease inhibitor combat viral clearance?

3. Introduction: "Excessive clotting" is an oversimplification. There is hardly any systemic coagulation activation in Covid19. There may be pulmonary hypercoagulability and enhanced fibrin turnover (see for example Lancet Haematol. 2020 Jun;7(6):e438-e440).

4. The clinical description of a SARS-CoV2 infection mostly relate tot he initial virus variants including alpha and delta. With the currently circulating virus (omicron variants) the described clinical features are rather rare.

5. Introduction: the concept of heparin resistance in Covid19 is highly debatable and not very well supported by sound data. Please delete from the introduction.

6. Please replace the outdated nomenclature antithrombin III by the current "antithrombin".

7. The authors should demonstrate any species-specific differences in binding characteristics between mice and humans before embarking on conclusions for human disease.

8. The dosing rationale of this study deserves a lot of explanation. In the human trial with Serp 1 (Tardif et al.) a single dose of 5 ug/kg =5 ng/gm was given. In the mice experiments reported in this manuscript the dose is 100 ng/gm per day for subsequent days, which is twentyfold higher. Also, the current agent is a PEGylated serpin which is supposedly more effective. The authors

should definitively include studies at a more relevant dosing range.

9. The clinical score is based on rather subjective observations, hence the study should have been performed in a blinded fashion. Were the observers aware of the treatment administered? Also, was treatment assignment randomized?

10. I am not sure saline is the correct control treatment. At least a PEGylated formulation should have been used as an appropriate control.

11. To substantiate a claim of an anticoagulopathic effect of Serp1 more experimental results are required. In fact, there authiors have not included an adequate analysis of coagulation and fibrinolysis nor pathology demonstrating fibrin deposition.

12. The discussion is rather long and deviates quite a bit from the results presented (e.g. sections on spike protein, lengthy discussions on uPA(r). I would uggest to focus a bit and condense the text considerably.

Dear Dr. Durdevic / Zeljko,

Re Manuscript re-submission to EMBO Translational Medicine

Title: **Viral anti-inflammatory serpin reduces immuno-coagulopathic pathology in SARS-CoV-2 mouse models of infection**

EMM-2023-17376

Thank you for your invitation to submit our revised manuscript. We have responded to the individual reviewers' comments and extensively modified the manuscript. With this work we are investigating treatment with a virus-derived serpin for the treatment of inflammation and coagulation mediated damage after severe viral ARDS with COVID-19 using mouse models for SARS-CoV-2, as presented at the Keystone Conference in Brussels. It is indeed a great pleasure to submit our revised manuscript to EMBO Molecular Medicine.

The reviewers' comments and point by point responses are provided below. The text, where modified in the manuscript, is noted as track changes. Longer section changes are also highlighted.

Referee #1

The manuscript by Zhang and Li, et al, examines the effect of PEGSerp-1 on disease outcome and coagulation factors in two mouse-adapted SARS-CoV-2 models. The topic is of interest to the SARS-CoV-2 treatment field, as coagulopathies are known to significantly contribute to mortality in ARDS patients. However, the manuscript is hampered by including too much background information and is very challenging to read as currently written. The following are suggestions to improve the readability and interpretation of the information presented.

Comments -

1. Overall, this manuscript contains too much detail and is very challenging to read and understand what all was performed as currently written. The manuscript includes several compound sentences that are too long and would benefit from being broken up into several shorter sentences. Additionally, the authors should carefully read through the manuscript and correct typographical errors. The Introduction is overly long and includes more detail than necessary, particularly regarding the different coagulation cascade proteins. Additionally, much of this information appears to be repeated in the Results sections. Reducing the length of the Introduction by half, focusing on only the information directly relevant to the presented research, would be much easier for the reader.

Response to Comment 1 – We thank the reviewer for these thoughtful comments. We have shortened the introduction and provided a more succinct revised Introduction. We have replaced compound sentences with separate individual sentences as suggested. Please see highlighted track changes.

2. Was the effect of PEGSerp-1 treatment examined after infection with SARS-CoV-2 (i.e. treatment started upon

commencement of clinical signs, 2 DPI)? This would be important in discerning whether PEGSerp-1 represent a potential treatment option for SARS-CoV-2 patients rather than just a proof-of-principle.

Response to Comment 2 – We thank the reviewer for this excellent suggestion and we have now performed a new third mouse study with the SARS-CoV-2 MA30 infection model, starting drug treatments at 2 days post-infection. As for Serp-1 treatment in the MHV68 infection model and PEGSerp-1 treatment in the pristane induced lupus lung hemorrhage models as previously published, we do observe improved outcomes (increased weight gain and reduced clinical scores) when PEGSerp-1 treatment (10ng/gm) is started two days post infection. This is now noted as follows on page 6 paragraphs 2 and 3 as follows and in the modified figure 2 that now incorporates data for all three SARS-CoV-2 MA30 models, as follows:

"In a third cohort of SARS-CoV-2 MA30 infected C57BL/6 mice, PEGSerp-1 treatment was given at doses of 0, 10 or 100ng/gm starting 2 days after initial virus infection (N = 24, 8 mice per treatment group; figure 2J-L). The 10ng/gm dose is comparable to the effective 15µg/kg doses given in the Phase 2 clinical trial of Serp-1 treatment in unstable coronary patients post stent implant. With this delayed PEGSerp-1 treatment, 10ng/gm doses demonstrated greater benefit with significantly improved weight and clinical scores at 4-6 days post infection (Fig 2K,L; $p < 0.0351$ to 0.0421). Higher dose PEGSerp-1 (100ng/gm) did not significantly affect weight loss although there was a trend towards increased weight gain the last 2 days ($p = 0.07$, ns). Area of lung consolidation was reduced on histologic analysis at the effective treatment dose with improved clinical score and weight loss (Fig 2J, $p < 0.0403$) Although the MA30 infection was initiated with an intended LD50 inoculum, infection related deaths were only observed in the third study with delayed treatment, 7/ 24 mice died with SARS infection in this group beginning at 4 days post infection. One mouse in the prophylaxis group died on day 0 when first inoculated with virus due to excess volume and respiratory distress (1/40). Overall, there was no significant change in mortality with treatment in either early or delayed PEGSerp-1 treatment (Kaplan-Meier $p = 0.28$, ns).

The results overall from the two cohorts of MA30 infected mice with PEGSerp-1 prophylactic treatment starting on the day of infection indicate consistent, significantly improved weight gain and clinical scores at days 2-5 and at days 4 to 6 post infection for delayed treatment. With delayed treatment in infected mice with established viremia, the lower dose PEGSerp-1 treatment significantly improved weight gain and reduced the clinical score. Lung consolidation area was reduced in mice where PEGSerp-1 improved weight gain and clinical score."

3. This reviewer finds it very surprising that the investigators did not observe any weight loss or clinical signs in the MA10-infected BALB/c mice, especially at such a high infection dose. Furthermore, the way the manuscript is written, it is confusing whether the data presented belongs to the MA10 or the MA30 model in each figure. The MA30 data appears to be more informative and more thoroughly examined of the two models. Therefore, this reviewer recommends the investigators consider moving the MA10 data to supplemental figures to help streamline the manuscript.

Response to 3 - We thank the reviewer for this comment, however, we would respectfully note that the MA10 model uses the BALB/c mouse background and thus provides a different immunoreactive model demonstrating reduced lung pathology in a strain of mice that have a differing T cell immune response (ie Th2-polarized) than in the C57BL/6 strain (Th1-polarized). The MA10 infection model is presented in a separate figure, Figure 4, to improve readability. We do agree that viral infection models can vary in differing institutions as to pathogenicity, but we must present the data as we recorded in our BSL3 facility. We would also note that a second reviewer supports the use of this second model and presentation of this data.

4. The inclusion of the Fig 9 data does not fit with the rest of the manuscript. Either the additional identified mammalian serpins should be examined in the MA30 model, or this data should be removed from the paper.

Response to 4 – We thank the reviewer for this comment. This data provides a potential insight into the mechanism of action of Serp-1 illustrating protease and serpin targets in a model of lung injury and hemorrhage, we do however agree that this data is derived from a differing lung injury model and we have therefore removed this figure.

Minor Points:

1. It is unclear from the manuscript text when treatment with PEGSerp-1 began. An experimental design schematic at the beginning of Figs 1 and 3 would help illustrate the study setup and make it easier for the reader to understand what was done.

Response to 1 – We thank the reviewer for this excellent suggestion and we have now provided a Flow chart as a separate initial figure – revised Figure 1.

2. Most of the histopathological images need to be white-balanced throughout the manuscript.

Response to 2 – we have now endeavored to white balance the histology images where possible. There is background pigment in the myocardium.

3. The colors and shapes of the data points on the graphs should be standardized across the manuscript.

Response to 3 – We thank the reviewer and the graphs are now standardized with shapes of points and colours.

4. Fig 1: The clinical score graphs should be presented as a stacked bar graph for each treatment group so that the reader may see the full range of scores at each day post infection.

Response to 4 – We thank the reviewer, however, we would note that the individual weights and clinical scores are provided in the now revised Figure 2, as both the individual scores as well as the mean + SD. The data points for individual mice do provide the full data range.

5. Fig 1: Presenting the 4 and 7 DPI body weights separately is not necessary since the results are the same. I recommend only the combined 4 and 7 DPI body weight be presented in the figure, with the individual animal weight and clinical score data moved to a supplemental figure.

Response to 5 – We thank the reviewer for this comment and we have now presented only the combined data for the 4 and 7 day follow up in the revised Figure 2 panel G. We have however retained the original data for the 7 day follow up as this illustrates the individual measurements and data range for this initial study. The individual data points for the day 4 follow up is now presented in a supplementary figure (Figure S2).

6. Figs 1, 2, 4, 5, 6, 7, 8: Because the specific p values are described in the text, the figures would look less busy if only stars were included to denote significance as opposed to the specific p values. Please remove the specific p values from the figure legends as well, and include a general description of what each star represents (e.g. *p<0.05, **p<0.01, etc).

Response to 6 - We have removed the specific p values in the revised figures, however we have retained some specific p values in the qPCR analyses in figures 8 and 9 figures, specifically where we have analyzed via ANOVA and post hoc analyses. Please see the revised figures.

7. Figs 2, 4, and 5: The current presentation of the data between d4 and d7 is confusing, especially because the y-axes are not consistent. For parameters for which both d4 and d7 data are presented, both timepoints should be presented on the same graph, with a 2-way ANOVA and multiple comparisons post-test performed for statistical analysis. This will allow the reader to critically examine the effect of PEGSerp-1 treatment over time.

Response to 7 - We again thank the reviewer for this excellent suggestion. We have retained the separate graphs for the iNOS IHC analyses in figure 3, Panels B and D to illustrate the effective changes seen with both day 4 and day 7 follow up cohorts (N = 40 mice). However, we have now provided combined day 4 and 7 follow up in the revised figures for the IHC analyses for CD4, CD8 and Ly6G, providing ANOVA and post hoc analyses between treated and untreated groups (Figure 3 H,I, J and Figure 6 C,E,F,K,L) to allow for comparison.

8. Figs 7 and 8: The gene expression data for the mock-treated and PEGSerp-1-treated mice should be normalized to the uninfected control mice (i.e. delta-delta CT method) rather than presenting the uninfected control mice data.

Response to 8 - We thank the reviewer for this suggestion, however, the uninfected control data is provided for a comparator. All qPCR data is normalized to the internal control GAPDH. These are now revised figures 8 and 9.

9. The Discussion section should include commentary on how well the MA10 and MA30 mouse models replicate coagulopathy features seen with severe SARS-CoV-2 infection.

Response to 9 – We thank the reviewer for this thoughtful and interesting comment. There has not been extensive work reported on coagulopathy in the MA10 and MA30 models. This work does demonstrate altered uPAR, fX and fibrinogen together with increased inflammation markers. Fibrinogen and fX levels have now been assessed on IHC analysis in the revised manuscript. Significant changes in detectable fibrinogen and fX levels were detected in lung sections with reductions produced by PEGSerp-1 treatment as noted on page 9, para 2 and illustrated in Figure 6 Panels E and F for days 4 and 7 follow up, as follows on page 6 last paragraph.

"Detectable changes in thrombotic pathway proteases were also assessed. No change in factor X (fX) staining (Figure 6E; ANOVA $p< 0.0008$) was seen at 4 days with prophylactic PEGSerp-1 treatments ($p = 0.5148$), but fX was significantly reduced at 7 days ($p < 0.0001$). A reduction in detectable fibrinogen was also seen at days 4 and 7 with PEGSerp-1 treatment, with significance at 7 days (Figure 6F; $p = 0.1653$ day 4; $p < 0.019$ day 7)."

10. To what BALB/c and C57BL/6 substrains did the mice used in the study belong? This is important, as different substrains show different levels of susceptibility to MA10 and MA30 infection.

Response to 10 - The C57BL/6 mice are supplied by JAX labs – substrain C57BL/6J. The BALB/c were also originally from JAXmice but were bred in house at ASU.

Referee #2 (Comments on Novelty/Model System for Author):

This study is an animal model study and therefore I assessed medical impact as "N/A", using a narrow definition. The model is appropriate and there are no ethical issues.

Referee #2 (Remarks for Author):

In this study, Zhang and co-workers examine the effects of administering pegylated Serp-1 (PEGSerp-1) to mice infected with adapted strains of SARS-CoV-2. Serp-1 is a member of the serpin superfamily of protease inhibitors, a viral serpin encoded by the rabbit myxomatosis virus. Serp-1 has been demonstrated to have strong anti-inflammatory activity in a number of animal models of disease, an activity mediated by its interactions with urinary-type plasminogen activator (uPA) and its cellular receptor (uPAR) and possibly by its inhibition of multiple serine proteases regulating coagulation and fibrinolysis. Zhang and co-workers examined PEGSerp-1 effects when administered intraperitoneally in SARS-CoV-2-infected mice. They found that PEGSerp-1 treatment significantly improved wellness scores and weight in infected mice versus vehicle (saline) controls. These pathophysiological changes correlated with reduced iNOS expression in lung and heart, reduced c5b-9+ indicators of complement activity, and reduced markers of M1 inflammatory macrophages in SERP-1 treated mice.

Major points

1. This is an interesting and timely study congruent with the emerging picture of thrombo-inflammatory damage in severe SARS-CoV-2 infection. It is a substantial study suited for publication as a full paper and not a short report.

The investigators are to be complimented on their diligence in using two strains of mice and two SARS-CoV-2 mouse-adapted models. Their data with respect to attenuation of weight loss and reduction in inducible nitric oxide synthase (iNOS) associated with PEGSerp-1 treatment is also clear, and some of their proposed mechanisms are reasonable given these data. In other cases, they looked for effects on many parameters and did not find them. These data are also important and add to the thoroughness of the report. However, the investigators unnecessarily complicate their findings by pointing out trends in the data. It would be simpler and more appropriate to class any finding with $p > 0.05$ as being not statistically significant. For one thing, there is no accepted definition of "trend" of which this reviewer is aware and Zhang et al apply it to data sets with $p = 0.08$ and even $p = 0.20$. Zhang et al have found many statistically significant results and do not need to address those that fail to reach statistical significance.

Response to 1 – We thank the reviewer for these supportive and very helpful comments. We have removed comments that refer to trends albeit we do find these trends of interest.

2. It was not clear to this reviewer if the "clinical score" used to assess the infected mice had been previously employed or if it was developed for this study. This point should be made more clearly. In either case, how has it been validated?

Response to 2 – We have adapted this clinical score from Moreau 2020 and this is now referenced in the manuscript as noted on page 18, para 1 and referenced as follows;

“Mice were weighed daily and assessed for clinical signs using a clinical score (adapted from Moreau GB, 2020)”

3. In the gene expression (qPCR) data, the investigators found a significant increase in uPAR and C1INH mRNA levels after PEGSerp-1 treatment in lung tissue and PAI-1 and C1INH levels in heart. However, while statistically significant, the increase in mean levels is on the order of 10%. How do they see this small change in mRNA levels being mechanistically linked to PEGSerp1 action and the observed amelioration of inflammation in the infected mice? For secreted proteins like C1INH and PAI-1 are these tissues a significant site of synthesis? In addition, the investigators use y axis scales that differ for each parameter in Figures 7 and 8 and do not start at zero; they should at least alert readers to this fact by inserting a note into the relevant figure legends.

Response to 3 – We thank the reviewer for these helpful comments. We have now commented where appropriate on the y axes. These serpins, PAI-1 and C1Inh, are widely expressed in inflammatory states and would be expected to be seen at elevated levels in the vasculature and lungs of mice with severe viral infections. The variability in the y axes is dependent upon the qPCR ratios as measured and is now noted in the legend for figure 8.

4. Most of the experimental design in the study is logical and clear. However, it is unclear to this reviewer why the results from a lupus diffuse alveolar hemorrhage model are presented at all (Figure 9) and these do not seem related to the current study.

Response to 4 – We thank the reviewer for this comment. The lupus model is a model of lung hemorrhage and coagulopathy. This lung damage model in our opinion provides some potential insight into the MOA. However, we do agree this is a differing lung injury model, and we have there removed Figure 9. Future studies are planned to directly assess protein to protein interactions in the SARS MA30 infection model.

Minor points

1. In Figure 7G the investigators perform an ANOVA comparing three data sets, one of which has n=1. Do they think this is appropriate? Even if some statisticians think it is mathematically defensible, is it best practice?

Response to 1 – We used ANOVA to confirm overall significance when comparing more than two groups. However, the major differences analyzed are for the treated and untreated samples used for analysis of changes in gene

expression. Thus, we have provided the ANOVA analyses together with the secondary Fishers PLSD or Student's te test analyses when comparing Saline and PEGSerp-1 treated mice with SARS-CoV-2 infection. We have also assessed for significance where two groups are compared using an unpaired Student's t test achieving similar results.

2. In the Discussion, there is a paragraph beginning "In this study..." and concluding with a citation of Tardif 2010 that is duplicated elsewhere in the paper and should be deleted.

Response to 2 - We agree. We have removed the redundant comment.

3. "Antithrombin III" is an outdated term for antithrombin (Serpin C1) and should be replaced or expanded.

Response to 3 – We have replaced ATII with AT.

4. Why are only 6 days of data shown in some figures when the investigators followed mice for either 4 or 7 days?

Response to 4 – Mice were sacrificed on day 7 and the investigators performing the studies in BSL3 were unable to complete full clinical scores due to the length of time necessary to complete the euthanizing of the animals and collection of specimens under the increased containment strictures. We agree that having day 7 data as well would be more satisfying but the trends are still quite clear by day 7.

.

5. What is lung consolidation? It would be helpful to a general readership to define this parameter.

Response to 5 – Lung consolidation is produced by the loss of open airspaces, eg loss of open alveoli. This is visible on histology as completely filled in alveoli – usually filled with inflammatory cells, coagulation factors, RBCs etc. These features are evident on the illustrated histology sections demonstrating consolidation and consolidation is defined as follows on page 5, para 1 when first used;

"Lung consolidation was defined as loss of open alveolar spaces due to leukocyte infiltrate with associated clotting and/or bleeding."

6. At the beginning of the Results section "YL, BH" appears in parentheses but it is unclear what this means - perhaps a flag to two investigators on the team to comment on the section during its writing? If so this should be removed.

Response to 6 – YL and BH established the models but we agree however that these author initials can be removed and this has now been done.

7. Is it appropriate to use a Student's unpaired t test if the data are not normally distributed? Wouldn't a Mann-Whitney or other non-parametric test be more appropriate?

Response to 7 - We thank the reviewer, however, the majority of statistical analyses were performed using ANOVA for comparison of data from more than two groups with post hoc analyses.
Our statisticians have advised the use of these analyses and we understand that the Mann Whitney is limited to comparison of two groups. We have however tested our analyses with ANOVA and for subgroup analysis or comparison of two treatment groups we have also used Student's t test as well as Mann Whitney as suggested. We see similar levels of significance using these alternative statistical analyses. Our statistical consultants indicate the approaches we have used are acceptable, and in some cases more stringent. Per our statistician "the Mann Whitney test may have been suggested owing to the fact that it is a nonparametric test, and thus has less stringent assumptions than an ANOVA. It certainly wouldn't be wrong to use it instead of ANOVA. If you are using ANOVA because you have more than two groups, then the equivalent of ANOVA would be the Kruskal-Wallis test."

8. In the first paragraph of the Introduction "can induce excess and damaging immune responses" should be "can induce excessive and damaging immune responses".

Response to 8 – This is now corrected,

Referee #3 (Comments on Novelty/Model System for Author):

In this manuscript the authors present the effect of PEGylated myxoma virus-derived Serp-1 as a potential pharmaceutical agent for reduction of inflammatory and coagulation activity in SARS-CoV2 infection. While the subject of this manuscript is interesting and potentially relevant, there are several experimental shortcomings, e.g. regarding the dose of the agent (and the lack of proper dose-response experiments), the choice of saline as a control substance, the lack of any investigation in anti-coagulopathic effects although these are claimed by the authors, and some methodological issues. I think the authors should at least add experiments to address these issues.

Response – We have now included an additional SARSCoV-2 MA30 infection study incorporating a dose response and a low dose comparable to the dose range used in the prior clinical study with the unmodified wild type Serp-1. Please see revised Results and revised figure 2 as follows;

""In a third cohort of SARS-CoV-2 MA30 infected C57BL/6 mice, PEGSerp-1 treatment was given at doses of 0, 10 or 100ng/gm starting 2 days after initial virus infection (N = 24, 8 mice per treatment group; figure 2J-L). The 10ng/gm dose is comparable to the effective 15µg/kg doses given in the Phase 2 clinical trial of Serp-1 treatment in unstable coronary patients post stent implant. With this delayed PEGSerp-1 treatment, 10ng/gm doses demonstrated greater benefit with significantly improved weight and clinical scores at 4-6 days post infection (Fig 2K,L; $p < 0.0351$ to 0.0421). Higher dose PEGSerp-1 (100ng/gm) did not significantly affect weight loss although there was a trend towards increased weight gain the last 2 days ($p = 0.07$, ns). Area of lung consolidation was reduced on histologic analysis at the effective treatment dose with improved clinical score and weight loss (Fig 2J, $p < 0.0403$) Although the MA30 infection was initiated with an intended LD50 inoculum, infection related deaths were only observed in the third study with delayed treatment, 7/ 24 mice died with SARS infection in this group beginning at 4 days post infection. One mouse in the prophylaxis group died on day 0 when first inoculated with virus due to excess volume and respiratory distress (1/40). Overall, there was no significant change in mortality with treatment in either early or delayed PEGSerp-1 treatment (Kaplan-Meier $p = 0.28$, ns).

The results overall from the two cohorts of MA30 infected mice with PEGSerp-1 prophylactic treatment starting on the day of infection indicate consistent, significantly improved weight gain and clinical scores at days 2-5 and at days 4 to 6 post infection for delayed treatment. With delayed treatment in infected mice with established viremia, the lower dose PEGSerp-1 treatment significantly improved weight gain and reduced the clinical score. Lung consolidation area was reduced in mice where PEGSerp-1 improved weight gain and clinical score."

Referee #3 (Remarks for Author):

The authors have investigated myxoma virus-derived Serp-1 as a potential pharmaceutical agent for reduction of inflammatory and coagulation activity in SARS-CoV2 infection. The following comments may be made:

1. I would suggest adding to the title of the manuscript that this was a study in mice.

Response to 1 – we have now added mice in the revised title as follows

"**Viral anti-inflammatory serpin reduces immuno-coagulopathic pathology in SARS-CoV-2 mouse models of infection"**

2. Abstract ",,,as a self-defense strategy to combat clearance" is not entirely understandable. Why would an antifibrinolytic protease inhibitor combat viral clearance?

Response to 2 - The protease inhibitors target a range of serine proteases and have proven immune modulating activity in myxoma viral infections. Additionally, the uPA and uPAR pathway is predominantly immune modulating. This is noted in the Introduction page 4 para 1 as follows

"Serp-1 is a myxoma virus-derived secreted 55kDa serpin glycoprotein that operates in virus-infected tissues by protecting the virus against activated myeloid cells (Nash 1997)."

And as follows in the discussion on page 16, para 1 " Of interest, the serpins are inhibitors that target active proteases, honing to sites of protease activation with postulated targeting to sites of immune and coagulation dysfunction. Thus, the poxvirus-derived Serp-1 protein evolved in myxoma virus as a secreted inhibitor of activated myeloid cells. Indeed, activated macrophages more effectively recognize and clear myxoma virus from infected tissues when the viral Serp-1 gene is deleted (Bouton 2023, Lucas 2004, Macen 1993)."

Serp-1 as noted also binds and inhibits thrombotic proteases and complement, thus providing a range of potential immune modulating actions.

3. Introduction: "Excessive clotting" is an oversimplification. There is hardly any systemic coagulation activation in Covid19. There may be pulmonary hypercoagulability and enhanced fibrin turnover (see for example Lancet Haematol. 2020 Jun;7(6):e438-e440).

Response to 3 - We have now referred to other studies indicating potential diffuse widespread coagulopathies that have been noted as potential contributing factors to systemic adverse effects with DVT and increased risk of cardiovascular damage in severe COVID19. We have now also included the reference to Levi et al in the Lancet where again abnormal coagulopathy is noted in severe illness.
Our understanding of the studies referenced is that there is some indication of excessive thrombosis and coagulopathy albeit not as dramatic as in some viral and bacterial infections (Arnold 2021, deBruina 2021, PHOSP-COVID Working Group 2022, Keskinidou 2021, Bradley 2019, Perico 2021, Kurtovic 2021, Jordan 2021)" Referenced in para 1 page 2. Several clinical studies have evaluated severe SARS with ICU admission and associated changes in inflammatory and coagulation together with uPAR level increases.

4. The clinical description of a SARS-CoV2 infection mostly relate tot he initial virus variants including alpha and delta. With the currently circulating virus (omicron variants) the described clinical features are rather rare.

Response to 4 - We agree with the reviewer, however, the initial severe SARS-2 infections as well as MERS and other viral infections with associated coagulopathy and higher mortality would suggest that systemic immune and coagulation disorders induced by severe viral infections deserve further careful study.

5. Introduction: the concept of heparin resistance in Covid19 is highly debatable and not very well supported by sound data. Please delete from the introduction.

Response to 5 - we thank the reviewer and we have removed this comment in the revision.

6. Please replace the outdated nomenclature antithrombin III by the current "antithrombin".

Response to 6 - ATIII is now referred to as AT.

7. The authors should demonstrate any species-specific differences in binding characteristics between mice and humans before embarking on conclusions for human disease.

Response to 7– We thank the reviewer and we will indeed consider these differences. However, at least for uPA and thrombin, Serp-1 has been assessed and found to bind to mouse, rabbit and human proteases as well as uPAR in human monocytes (see reference Viswanathan et al 2009). We do however agree that for eventual applications in man one

cannot predict final proven efficacy based on rodent models alone. Serp-1 has been tested in a small clinical trial for unstable plaque with successful outcomes in cardiovascular patients. We have commented on this now in the revised Discussion.

8. The dosing rationale of this study deserves a lot of explanation. In the human trial with Serp 1 (Tardif et al.) a single dose of 5 ug/kg =5 ng/gm was given. In the mice experiments reported in this manuscript the dose is 100 ng/gm per day for subsequent days, which is twentyfold higher. Also, the current agent is a PEGylated serpin which is supposedly more effective. The authors should definitively include studies at a more relevant dosing range.

Response to 8- We thank the reviewer for this comment. We have used the current dose in multiple models demonstrating efficacy (Guo 2021, Dai 2006, Yaron 2020 and 2021). The effective dose in the clinical trial was 15μg/kg daily for 3 days. However, we have also now included a dose titration study including the dose equivalent used in the prior clinical trial with wild type Serp-1. Please see the revised figure 2, panels J-L. We have found efficacy at this dose of PEGSerp-1 (10μg/kg, 10ng/gm) a dose in a comparable range to the dose used in the prior clinical trial for cardiovascular patients with the Serp-1 (nonPEGylated) (15 microgram/ kg).

9. The clinical score is based on rather subjective observations, hence the study should have been performed in a blinded fashion. Were the observers aware of the treatment administered? Also, was treatment assignment randomized?

Response – These studies were performed in a blinded fashion as noted in the text on page 18, para 1 as follows "For each MA30 study BSL3 treatments and the histopathology were blinded to treatment. For the MA10 study the histology analysis was blinded."

10. I am not sure saline is the correct control treatment. At least a PEGylated formulation should have been used as an appropriate control.

Response to 10 – The BSL3 work does restrict the number of animals that can be studied due to the complexity of work. Thus, we have focused on this first series of studies of PEGylated Serp-1 treatment when compared to saline. However, we have published prior work with several Serp-1 RCL mutants that lack normal serpin activity and as referenced. We have also previously compared the mammalian serpin, neuroserpin, with Serp-1 in a MHV68 mouse infection model. In future work, we will of course be very interested in comparing EGSerp-1 with other mammalian serpins such as C1Inh or A1AT or the inactive Serp-1 SAA RCL protein.

11. To substantiate a claim of an anticoagulopathic effect of Serp1 more experimental results are required. In fact, there authiors have not included an adequate analysis of coagulation and fibrinolysis nor pathology demonstrating fibrin deposition.

Response to 11 – We thank the reviewer for this comment and we agree with this suggestion. We have now included a histological analysis of fX and fibrinogen in the revised MS. This data is now provided in Figure 6 panels E and F and now discussed as follows on page 9, para 2 as follows. A tail vein bleeding time would indeed be helpful but is not allowed in our ABSL3 facility.

"Detectable changes in thrombotic pathway proteases were also assessed. No change in factor X (fX) staining (Figure 6E; ANOVA $p< 0.0008$) was seen with prophylactic PEGSerp-1 treatments at 4 days ($p = 0.5148$), but fX was significantly reduced at 7 days ($p < 0.0001$). A reduction in detectable fibrinogen was also seen at days 4 and 7 with PEGSerp-1 treatment, with significance at 7 days (Figure 6F; $p = 0.1653$ day 4; $p < 0.019$ day 7)."

12. The discussion is rather long and deviates quite a bit from the results presented (e.g. sections on spike protein, lengthy discussions on uPA(r). I would suggest to focus a bit and condense the text consid

Response to 12– we thank the reviewer and we have condensed the Discussion and focused on the findings in the revised MS. We would note that in prior work, Serp-1 wild type activity in mouse models was dependent upon the uPAR as evident in uPAR deficient mouse models as well as in THPS monocyte transmigration assays (Dai 2006; Viswanathan 2009, Yaron 2020 and 2021)

We thank you for consideration of our revised manuscript now resubmitted for review.

Sincerely,

Alexandra Lucas, MD, FRCP(C)

Professor
Biodesign Institute, Centers for Personalized Diagnostics
and Immunotherapy, Vaccines and Virotherapy
Arizona State University
Cell- 352-672-2301
Email - alexluc1@asu.edu

3rd Jul 2023

Dear Prof. Lucas,

Thank you for the submission of your revised manuscript to EMBO Molecular Medicine. I am pleased to inform you that we will be able to accept your manuscript pending the following final amendments:

1) Please address the referee #1 minor suggestions.
2) Figures: Please upload all the figures in PDF format.
3) Author: Please make sure that names of the authors are displayed in the same way in the manuscript file and our submission system. Currently, Bertram Jacobs is in the manuscript and Bert Jacobs in our system. Please resolve the name discrepancy.
4) In the main manuscript file, please do the following:
- Correct/answer all track changes suggested by our data editors by working from the attached document. Information about the nature and the number of replicates is missing.
- Add callouts for Fig. 5G. All figures and panels should be called out in a sequential order. Currently Fig. 9 panels are not in a sequential order and Fig 4E is called out before Fig. 4C-D. Please correct.
- Please place data availability section before "Acknowledgements" and if no data are deposited in a public repository, please add the sentence: This study includes no data deposited in external repositories.
5) Tables: Please remove separately uploaded files for Appendix figures and tables and only leave the Appendix file. Also, update all their callouts in the main manuscript text to Appendix Figure S1 etc. And Appendix Table S1 etc.
6) The Paper Explained: Please provide "The Paper Explained" and add it to the main manuscript text. Please refer to any of our published primary research articles for an example. Check "Author Guidelines" for more information.
https://www.embopress.org/page/journal/17574684/authorguide#researcharticleguide
7) Synopsis: Every published paper now includes a 'Synopsis' to further enhance discoverability. Synopses are displayed on the journal webpage and are freely accessible to all readers. They include separate synopsis image and synopsis text.
- Synopsis image: Please provide a striking image or visual abstract as a high-resolution jpeg file 550 px-wide x (250-400)-px high to illustrate your article.
- Synopsis text: Please provide a short standfirst (maximum of 300 characters, including space) as well as 2-5 one sentence bullet points that summarise the paper as a .doc file. Please write the bullet points to summarise the key NEW findings. They should be designed to be complementary to the abstract - i.e. not repeat the same text. We encourage inclusion of key acronyms and quantitative information (maximum of 30 words / bullet point). Please use the passive voice.
- Please check your synopsis text and image before submission with your revised manuscript. Please be aware that in the proof stage minor corrections only are allowed (e.g., typos).
8) For more information: This space should be used to list relevant web links for further consultation by our readers. Could you identify some relevant ones and provide such information as well? Some examples are patient associations, relevant databases, OMIM/proteins/genes links, author's websites, etc...
9) As part of the EMBO Publications transparent editorial process initiative (see our Editorial at http://embomolmed.embopress.org/content/2/9/329), EMBO Molecular Medicine will publish online a Review Process File (RPF) to accompany accepted manuscripts. This file will be published in conjunction with your paper and will include the anonymous referee reports, your point-by-point response and all pertinent correspondence relating to the manuscript. Let us know whether you agree with the publication of the RPF and as here, if you want to remove or not any figures from it prior to publication. Please note that the Authors checklist will be published at the end of the RPF.
10) Please provide a point-by-point letter INCLUDING my comments as well as the reviewer's reports and your detailed responses (as Word file).

I look forward to reading a new revised version of your manuscript as soon as possible.

Yours sincerely,

Zeljko Durdevic

Zeljko Durdevic
Editor
EMBO Molecular Medicine

*** Instructions to submit your revised manuscript ***

*** PLEASE NOTE *** As part of the EMBO Publications transparent editorial process initiative (see our Editorial at https://www.embopress.org/doi/pdf/10.1002/emmm.201000094), EMBO Molecular Medicine will publish online a Review Process File to accompany accepted manuscripts.

In the event of acceptance, this file will be published in conjunction with your paper and will include the anonymous referee reports, your point-by-point response and all pertinent correspondence relating to the manuscript. If you do NOT want this file to be published, please inform the editorial office at contact@embomolmed.org.

When submitting your revised manuscript, please include:

1) a .docx formatted version of the manuscript text (including Figure legends and tables)

2) Separate figure files*

3) supplemental information as Expanded View and/or Appendix. Please carefully check the authors guidelines for formatting Expanded view and Appendix figures and tables at https://www.embopress.org/page/journal/17574684/authorguide#expandedview

4) a letter INCLUDING the reviewer's reports and your detailed responses to their comments (as Word file).

5) The paper explained: EMBO Molecular Medicine articles are accompanied by a summary of the articles to emphasize the major findings in the paper and their medical implications for the non-specialist reader. Please provide a draft summary of your article highlighting
- the medical issue you are addressing,
- the results obtained and
- their clinical impact.
This may be edited to ensure that readers understand the significance and context of the research.
Please refer to any of our published articles for an example.

6) For more information: There is space at the end of each article to list relevant web links for further consultation by our readers. Could you identify some relevant ones and provide such information as well? Some examples are patient associations, relevant databases, OMIM/proteins/genes links, author's websites, etc...

7) Author contributions: the contribution of every author must be detailed in a separate section.

8) EMBO Molecular Medicine now requires a complete author checklist (https://www.embopress.org/page/journal/17574684/authorguide) to be submitted with all revised manuscripts. Please use the checklist as guideline for the sort of information we need WITHIN the manuscript. The checklist should only be filled with page numbers were the information can be found. This is particularly important for animal reporting, antibody dilutions (missing) and exact values and n that should be indicted instead of a range.

9) Every published paper now includes a 'Synopsis' to further enhance discoverability. Synopses are displayed on the journal webpage and are freely accessible to all readers. They include a short stand first (maximum of 300 characters, including space) as well as 2-5 one sentence bullet points that summarise the paper. Please write the bullet points to summarise the key NEW findings. They should be designed to be complementary to the abstract - i.e. not repeat the same text. We encourage inclusion of key acronyms and quantitative information (maximum of 30 words / bullet point). Please use the passive voice. Please attach these in a separate file or send them by email, we will incorporate them accordingly.

You are also welcome to suggest a striking image or visual abstract to illustrate your article. If you do please provide a jpeg file 550 px-wide x 400-px high.

10) A Conflict of Interest statement should be provided in the main text

11) Please note that we now mandate that all corresponding authors list an ORCID digital identifier. This takes <90 seconds to complete. We encourage all authors to supply an ORCID identifier, which will be linked to their name for unambiguous name identification.

Currently, our records indicate that the ORCID for your account is 0000-0002-5195-1442.

Please click the link below to modify this ORCID:
Link Not Available

12) The system will prompt you to fill in your funding and payment information. This will allow Wiley to send you a quote for the article processing charge (APC) in case of acceptance. This quote takes into account any reduction or fee waivers that you may be eligible for. Authors do not need to pay any fees before their manuscript is accepted and transferred to our publisher.

*Additional important information regarding Figures

Each figure should be given in a separate file and should have the following resolution:
Graphs 800-1,200 DPI
Photos 400-800 DPI
Colour (only CMYK) 300-400 DPI"

Figures are not edited by the production team. All lettering should be the same size and style; figure panels should be indicated by capital letters (A, B, C etc). Gridlines are not allowed except for log plots. Figures should be numbered in the order of their appearance in the text with Arabic numerals. Each Figure must have a separate legend and a caption is needed for each panel.

*Additional important information regarding figures and illustrations can be found at https://bit.ly/EMBOPressFigurePreparationGuideline. See also figure legend preparation guidelines: https://www.embopress.org/page/journal/17574684/authorguide#figureformat

The system will prompt you to fill in your funding and payment information. This will allow Wiley to send you a quote for the article processing charge (APC) in case of acceptance. This quote takes into account any reduction or fee waivers that you may be eligible for. Authors do not need to pay any fees before their manuscript is accepted and transferred to our publisher.

***** Reviewer's comments *****

Referee #1 (Remarks for Author):

The revised manuscript submitted by Zhang and colleagues is much improved, and overall my comments were adequately addressed. Only a few minor suggestions remain:

1. The white balance is still not sufficient in the majority of histopathological/IHC figures. The images in Figure 6A are perfectly balanced, and the rest (particularly Figs 2E, 2I, etc) should be similarly balanced. A quick consultation with a pathologist should help if the authors are uncertain how to perform white balancing.

2. Please add the specific BALB/c substrain to the Materials and Methods (e.g. BALB/cJ, BALB/cByJ), as several BALB/c substrains are available from Jackson labs, and they show very different responses to MA10 infection.

Referee #2 (Remarks for Author):

The authors have made substantial modifications to their revised manuscript and have addressed all points raised in the review process satisfactorily.

Referee #3 (Remarks for Author):

The authors have responded to my questions and concerns satisfactorily and have added additional experiments. I have no further comments.

July 9, 2023

Dear Dr. Durdevic / Zeljko,

Re Manuscript re-submission to EMBO Translational Medicine

Title: **Viral anti-inflammatory serpin reduces immuno-coagulopathic pathology in SARS-CoV-2 mouse models of infection**

EMM-2023-17376

Thank you for your letter of acceptance of our revised manuscript. We have responded to the additional corrections as well as incorporating the prior revisions as suggested by the reviewers. We have addressed the two remaining minor comments from Reviewer # 1 and have incorporated the suggested changes from the editorial staff.

The additional comments and corrections on the revised manuscript together with the prior reviewers' comments and the point by point responses to the initial reviews are provided below. The text, where modified in the manuscript, is noted with highlighting. Longer section changes are also highlighted.

Responses to the review of the revised manuscript.

1) Please address the referee #1 minor suggestions.

Response to Comment 1 - These minor comments are now addressed -

- The exact BALB/c mouse strain is now provided in the Methods section as requested, as follows "For MA10 infection 2x10^5 *pfu was inoculated by intranasal route (IN) in BALB/cAnNCrl (Leist 2020)."*

- The histology images have been modified to improve the white balance Extensive modification of color reduces the differentiation of positively stained cells, thus we have improved the contrast and balance but cannot change the images extensively without losing the differentiation. These modified histology images are now attached with the re-revised manuscript.

14th Jul 2023

Dear Prof. Lucas,

We are pleased to inform you that your manuscript is accepted for publication and is now being sent to our publisher to be included in the next available issue of EMBO Molecular Medicine.

Please read below for additional IMPORTANT information regarding your article, its publication and the production process.

Congratulations on your interesting work,

Zeljko Durdevic

Zeljko Durdevic
Editor
EMBO Molecular Medicine

Follow us on Twitter @EmboMolMed
Sign up for eTOCs at embopress.org/alertsfeeds

*** *** *** IMPORTANT INFORMATION *** *** ***

SPEED OF PUBLICATION
The journal aims for rapid publication of papers, using using the advance online publication "Early View" to expedite the process: A properly copy-edited and formatted version will be published as "Early View" after the proofs have been corrected. Please help the Editors and publisher avoid delays by providing e-mail address(es), telephone and fax numbers at which author(s) can be contacted.

Should you be planning a Press Release on your article, please get in contact with embomolmed@wiley.com as early as possible, in order to coordinate publication and release dates.

LICENSE AND PAYMENT:

All articles published in EMBO Molecular Medicine are fully open access: immediately and freely available to read, download and share.

EMBO Molecular Medicine charges an article processing charge (APC) to cover the publication costs. You, as the corresponding author for this manuscript, should have already received a quote with the article processing fee separately. Please let us know in case this quote has not been received.

Once your article is at Wiley for editorial production you will receive an email from Wiley's Author Services system, which will ask you to log in and will present you with the publication license form for completion. Within the same system the publication fee can be paid by credit card, an invoice, pro forma invoice or purchase order can be requested.

Payment of the publication charge and the signed Open Access Agreement form must be received before the article can be published online.

PROOFS

You will receive the proofs by e-mail approximately 2 weeks after all relevant files have been sent o our Production Office. Please return them within 48 hours and if there should be any problems, please contact the production office at embopressproduction@wiley.com.

Please inform us if there is likely to be any difficulty in reaching you at the above address at that time. Failure to meet our

deadlines may result in a delay of publication.

All further communications concerning your paper proofs should quote reference number EMM-2023-17376-V3 and be directed to the production office at embopressproduction@wiley.com.

Thank you,

Zeljko Durdevic
Editor
EMBO Molecular Medicine

EMBO Press Author Checklist

Corresponding Author Name: Alexandra Lucas, MD and Brenda G Hogue, PhD	
Journal Submitted to: EMBO Molecular Mediicine	
Manuscript Number: EMM-2023-17376-V2	

Reporting Checklist for Life Science Articles (updated January 2022)

This checklist is adapted from Materials Design Analysis Reporting (MDAR) Checklist for Authors. MDAR establishes a minimum set of requirements in transparent reporting in the life sciences (see Statement of Task: 10.31222/osf.io/9sm4x). Please follow the

Please note that a copy of this checklist will be published alongside your article.

Abridged guidelines for figures

1. Data

The data shown in figures should satisfy the following conditions:

- the data were obtained and processed according to the field's best practice and are presented to reflect the results of the experiments in an accurate and unbiased manner.
- ideally, figure panels should include only measurements that are directly comparable to each other and obtained with the same assay.
- plots include clearly labeled error bars for independent experiments and sample sizes. Unless justified, error bars should not be shown for technical replicates.
- if n<5, the individual data points from each experiment should be plotted. Any statistical test employed should be justified.
- Source Data should be included to report the data underlying figures according to the guidelines set out in the authorship guidelines on Data Presentation.

To the best of our understanding we have conformed to all data requirements for each figure - Source data is also supplied

2. Captions

Each figure caption should contain the following information, for each panel where they are relevant:

- a specification of the experimental system investigated (eg cell line, species name).
- the assay(s) and method(s) used to carry out the reported observations and measurements.
- an explicit mention of the biological and chemical entity(ies) that are being measured.
- an explicit mention of the biological and chemical entity(ies) that are altered/varied/perturbed in a controlled manner.
- the exact sample size (n) for each experimental group/condition, given as a number, not a range;
- a description of the sample collection allowing the reader to understand whether the samples represent technical or biological replicates (including how many animals, litters, cultures, etc.).
- a statement of how many times the experiment shown was independently replicated in the laboratory.
- definitions of statistical methods and measures:
  - common tests, such as t-test (please specify whether paired vs. unpaired), simple χ2 tests, Wilcoxon and Mann-Whitney tests, can be unambiguously identified by name only, but more
  - are tests one-sided or two-sided?
  - are there adjustments for multiple comparisons?
  - exact statistical test results, e.g., P values = x but not P values < x;
  - definition of 'center values' as median or average;
  - definition of error bars as s.d. or s.e.m.

All figures contain the experimental system, the assays, the measured chemical entities, the N number for sample sizes, the statistical methods and p values, the SD and SEM are defined, the mean values

Please complete ALL of the questions below.

Materials

Newly Created Materials	Information included in the manuscript?\\	In which section is the information available? (Reagents and Tools Table, Materials and Methods, Figures, Data Availability Section)
New materials and reagents need to be available; do any restrictions apply?	Not Applicable	New materials are available for other labs if sufficint quantities are available

Antibodies	Information included in the manuscript?	In which section is the information available? (Reagents and Tools Table, Materials and Methods, Figures, Data Availability Section)
For **antibodies** provide the following information:<br>- Commercial antibodies: RRID (if possible) or supplier name, catalogue number and or/clone number<br>- Non-commercial: RRID or citation	Yes	Materials and Methods

DNA and RNA sequences	yes	In which section is the information available? (Reagents and Tools Table, Materials and Methods, Figures, Data Availability Section)
**Short novel DNA or RNA including primers, probes**: provide the sequences.	Yes	Materials and Methods

Cell materials	yes	In which section is the information available? (Reagents and Tools Table, Materials and Methods, Figures, Data Availability Section)
**Cell lines:** Provide species information, strain. Provide accession number in repository **OR** supplier name, catalog number, clone number, and/**OR** RRID.	Yes	Materials and Methods
**Primary cultures:** Provide species, strain, sex of origin, genetic modification status.	Not Applicable	
Report if the cell lines were recently **authenticated** (e.g., by STR profiling) and tested for mycoplasma contamination.	Yes	

Experimental animals	JAXLabs mouse strains	Materials nd Methods
**Laboratory animals or Model organisms:** Provide species, strain, sex, age, genetic modification status. Provide accession number in repository **OR** supplier name, catalog number, clone number, **OR** RRID.	Yes	Materials and Methods
**Animal observed in or captured from the field:** Provide species, sex, and age where possible.	Not Applicable	
Please detail **housing and husbandry conditions**.	Not Applicable	

Plants and microbes	Virus isolates	Materials and Methods
**Plants:** provide species and strain, ecotype and cultivar where relevant, unique accession number if available, and source (including location for collected wild	Not Applicable	
**Microbes:** provide species and strain, unique accession number if available, and source.	Not Applicable	

Human research participants	Information included in the manuscript?	In which section is the information available? (Reagents and Tools Table, Materials and Methods, Figures, Data Availability Section)
If collected and within the bounds of privacy constraints report on age, sex and gender or ethnicity for all study participants.	Not Applicable	

Core facilities	Information included in the manuscript?	In which section is the information available? (Reagents and Tools Table, Materials and Methods, Figures, Data Availability Section)
If your work benefited from core facilities, was their service mentioned in the acknowledgments section?	Yes	Materials and Methods

Design

Study protocol	Information included in the manuscript?	In which section is the information available? (Reagents and Tools Table, Materials and Methods, Figures, Data Availability Section)
If study protocol has been **pre-registered, provide DOI in the manuscript**. For clinical trials, provide the trial registration number **OR** cite DOI.	Not Applicable	
Report the **clinical trial registration number** (at ClinicalTrials.gov or equivalent), where applicable.	Not Applicable	

Laboratory protocol	Information included in the manuscript?	In which section is the information available? (Reagents and Tools Table, Materials and Methods, Figures, Data Availability Section)
Provide DOI OR other citation details if **external detailed step-by-step protocols** are available.	Not Applicable	

Experimental study design and statistics	Information included in the manuscript?	Materials and Methods
Include a statement about **sample size** estimate even if no statistical methods were used.	Yes	
Were any steps taken to minimize the effects of subjective bias when allocating animals/samples to treatment (e.g. **randomization procedure**)? If yes, have they been described?	Yes	All BSL3 animal studies were blinded
Include a statement about **blinding** even if no blinding was done.	Yes	Statement included
Describe **inclusion/exclusion criteria** if samples or animals were excluded from the analysis. Were the criteria pre-established?<br><br>If sample or data points were omitted from analysis, report if this was due to attrition or intentional exclusion and provide justification.	Not Applicable	No data was excluded

For every figure, are **statistical tests** justified as appropriate? Do the data meet the assumptions of the tests (e.g., normal distribution)? Describe any methods used to assess it. Is there an estimate of variation within each group of data? Is the variance similar between the groups that are being statistically compared?	Not Applicable	

Sample definition and in-laboratory replication	Information included in the manuscript?	In which section is the information available? (Reagents and Tools Table, Materials and Methods, Figures, Data Availability Section)
In the figure legends: state number of times the experiment was **replicated** in laboratory.	Yes	Replication numbers described in the Materials and Methods and in the Results
In the figure legends: define whether data describe **technical or biological replicates**.	Yes	

Ethics

Ethics	Information included in the manuscript?	In which section is the information available? (Reagents and Tools Table, Materials and Methods, Figures, Data Availability Section)
Studies involving **human participants**: State details of **authority granting ethics approval** (IRB or equivalent committee(s), provide reference number for approval.	Not Applicable	
Studies involving **human participants**: Include a statement confirming that **informed consent** was obtained from all subjects and that the experiments conformed to the principles set out in the WMA Declaration of Helsinki and the Department of Health and Human Services Belmont Report.	Not Applicable	
Studies involving **human participants:** For publication of **patient photos**, include a statement confirming that consent to publish was obtained.	Not Applicable	
Studies involving experimental **animals**: State details of **authority granting ethics approval** (IRB or equivalent committee(s), provide reference number for approval. Include a statement of compliance with ethical regulations.	Yes	IACUC approval provided in Materials and Methods
Studies involving **specimen and field samples:** State if relevant **permits** obtained, provide details of authority approving study; if none were required, explain why.	Not Applicable	

Dual Use Research of Concern (DURC)	Information included in the manuscript?	Materials and Methods
Could your study fall under dual use research restrictions? Please check biosecurity documents and list of **select agents and toxins** (CDC): https://www.selectagents.gov/sat/list.htm	Not Applicable	
If you used a select agent, is the security level of the lab appropriate and reported in the manuscript?	Not Applicable	
If a study is subject to dual use research of concern regulations, is the name of the **authority granting approval and reference number** for the regulatory approval provided in the manuscript?	Not Applicable	

Reporting

The MDAR framework recommends adoption of discipline-specific guidelines, established and endorsed through community initiatives. Journals have their own policy about requiring specific guidelines and recommendations to complement MDAR.

Adherence to community standards	Information included in the manuscript?	In which section is the information available? (Reagents and Tools Table, Materials and Methods, Figures, Data Availability Section)
State if relevant guidelines or checklists (e.g., **ICMJE, MIBBI, ARRIVE, PRISMA**) have been followed or provided.	Not Applicable	
For **tumor marker prognostic studies,** we recommend that you follow the **REMARK** reporting guidelines (see link list at top right). See author guidelines, under 'Reporting Guidelines'. Please confirm you have followed these guidelines.	Not Applicable	
For **phase II and III randomized controlled trials**, please refer to the **CONSORT** flow diagram (see link list at top right) and submit the CONSORT checklist (see link list at top right) with your submission. See author guidelines, under 'Reporting Guidelines'. Please confirm you have submitted this list.	Not Applicable	

Data Availability

Data availability	Information included in the manuscript?	In which section is the information available? (Reagents and Tools Table, Materials and Methods, Figures, Data Availability Section)
Have **primary datasets** been deposited according to the journal's guidelines (see 'Data Deposition' section) and the respective accession numbers provided in the Data Availability Section?	Yes	
Were **human clinical and genomic datasets** deposited in a public access-controlled repository in accordance to ethical obligations to the patients and to the applicable consent agreement?	Not Applicable	
Are **computational models** that are central and integral to a study available without restrictions in a machine-readable form? Were the relevant accession numbers or links provided?	Not Applicable	
If publicly available data were reused, provide the respective **data citations in the reference list**.	Not Applicable	
